# Piezo‐Coupling Effects in Smart Polymers: Toward Ultrasensitive Sensing and Biomedical Applications

**DOI:** 10.1002/advs.77113

**Published:** 2026-08-17

**Authors:** Duc Khanh Tran, Tuan Sang Tran, Thanh‐An Truong, Trung‐Hieu Vu, That Buu Ton, Dzung Viet Dao, Van Thanh Dau

**Affiliations:** ^1^ School of Engineering and Built Environment Griffith University Brisbane Queensland Australia; ^2^ Queensland Quantum and Advanced Technologies Research Institute Griffith University Brisbane Queensland Australia

**Keywords:** biomedical devices, piezo‐coupling effects, piezoelectricity, self‐powered sensors, smart polymers

## Abstract

The integration of piezoelectricity with other physical effects—such as pyroelectric, triboelectric, and photovoltaic effects—has enabled the development of multifunctional, self‐powered sensors with enhanced sensitivity and application versatility. Central to this advancement is the use of stimuli‐responsive smart polymers, which offer mechanical flexibility, biocompatibility, and tuneable properties essential for wearable and implantable biomedical applications. By harnessing piezo‐coupling effects in stimuli‐responsive polymers, these systems overcome the current limitations of monofunctional rigid sensors, offering reduced structural complexity while improving sensitivity and energy efficiency. This review outlines the fundamental principles of these piezo‐coupling effects, highlights key stimuli‐responsive polymeric materials with fabrication strategies, and discusses recent progress in device architectures tailored for ultrasensitive sensing and biomedical applications. Finally, it addresses current challenges and future directions for scalable, ultrasensitive, and multifunctional polymer‐based sensing platforms.

## Graphical abstract

Piezo‐coupling effects are the foundational factors for the development of multifunctional and self‐powered platforms. Central to this advancement is the use of stimuli‐responsive smart polymers, which offer mechanical flexibility, biocompatibility, and simplified structural complexity with improved sensitivity and energy efficiency. This review article outlines the fundamental principles of these piezo‐coupling effects, emphasizes key stimuli‐responsive polymeric materials with fabrication strategies, and highlights recent progress. Finally, it addresses current challenges and future perspectives for scalable, ultrasensitive, and multifunctional polymer‐based sensing platforms.

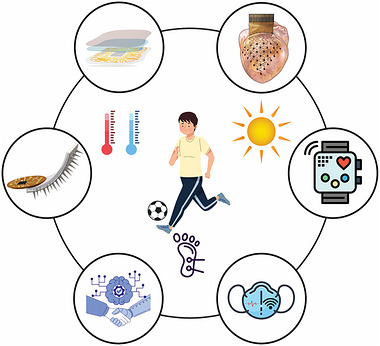

## 1 Introduction

The rapid advancement of wearable electronics and biomedical technologies has intensified the demand for ultrasensitive sensors capable of detecting, distinguishing, and responding to diverse stimuli, including mechanical, thermal, optical, and biochemical [1, 2, 3, 4]. Traditional monofunctional sensors, which rely on a single transduction mechanism (e.g., piezoelectric, pyroelectric, triboelectric, or photovoltaic effect), are limited in versatility and often require complex sensing arrays to achieve high sensitivity and multi‐stimuli detection [5, 6, 7, 8]. However, single‐purpose sensors have restricted detection input; even when combined, the sensor arrays have complicated structures, signal interference, crosstalk, and noise accumulation [9, 10, 11]. To overcome these limitations, multi‐effect coupling has emerged as a promising approach for ultrasensitive sensing. By integrating multiple transduction mechanisms within a single device, these sensors harness synergistic effects to enhance sensitivity, reduce noise, and simplify design [12, 13, 14, 15]. Among the various coupling strategies, the piezo‐coupling effects, where the piezoelectric effect is combined with other physical effects such as the pyroelectric, triboelectric, and photovoltaic effects, have shown exceptional potential for developing ultrasensitive, flexible, and multifunctional sensing platforms. Piezoelectric materials generate electrical signals in response to mechanical deformation, making them ideal for detecting pressure, strain, and vibration [16]. When coupled with pyroelectric, which arise from temperature‐induced changes in spontaneous polarization, the piezo‐pyroelectric effect can simultaneously respond to mechanical and thermal stimuli. The synergistic dual‐coupling mechanism enhanced sensor performance under simultaneous impetuses, thus widening their applications in fire detection and health monitoring [17, 18, 19]. Similarly, piezo‐coupling with the triboelectric phenomenon, which relies on contact electrification and electrostatic induction, enables piezo‐triboelectric effects with high‐output, self‐powered sensing of dynamic mechanical interactions. Notably, this synergistic interaction could be readily achieved since most materials possess electron affinities, facilitating efficient charge generation and transfer [20, 21, 22, 23]. Piezo‐coupling with photovoltaic effects, which is based on light‐induced charge carrier generation and separation, facilitates the piezo‐phototronic effect to expand the sensing capabilities toward optical stimuli and energy harvesting from ambient light. Under strain, the piezoelectric potential increases the separation and transportation of photovoltaic charge carriers, leading to higher output [24, 25, 26]. Recently, researchers have put efforts in combining piezoelectric with multiple effects at once, such as piezo‐pyro‐phototronic or piezo‐tribo‐photovoltaic, where piezoelectric and pyroelectric are responsible for charge carriers while triboelectricity and photoexcitation generate the charges.

**FIGURE 1:**
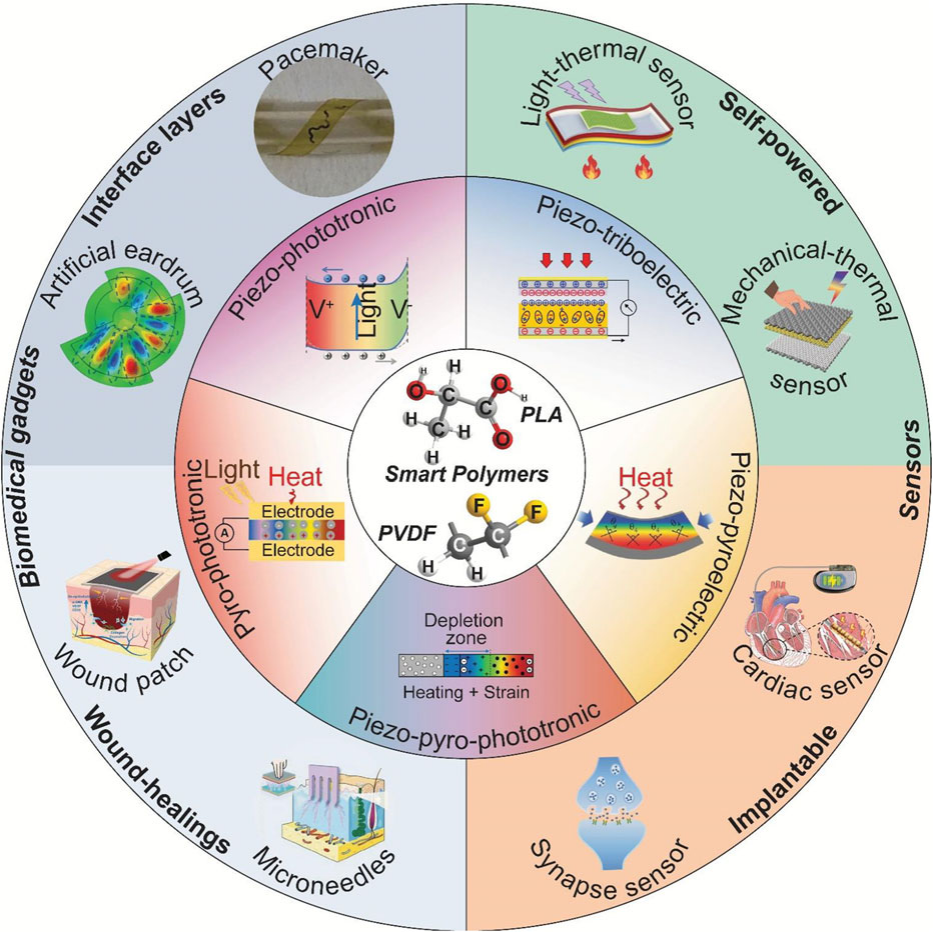
Inner circle: Different multi‐physics coupling effects: Piezo‐phototronic. Reproduced with permission [27]. Copyright 2020, John Wiley and Sons; Piezo‐triboelectric. Reproduced with permission [28]. Copyright 2024, Elsevier; Piezo‐pyroelectric. Reproduced with permission [29]. Copyright 2014, John Wiley and Sons; Piezo‐pyro‐phototronic. Reproduced with permission [30]. Copyright 2023, Elsevier; Pyro‐phototronic. Reproduced with Creative Commons CC BY‐NC‐ND license [31]. Copyright 2023, Elsevier. Outer circle: various applications of the polymers with piezo‐coupling effects (clockwise): Self‐powered piezo‐pyro‐phototronic transparent mechanoreceptor and self‐powered piezo‐phototronic photodetector. Reproduced with permission [32]. Copyright 2022, Elsevier; Implantable sensors into different body parts, such as piezo‐triboelectric cardiac and piezo‐phototronic synapse sensors. Reproduced with permission [33]. Copyright 2021, John Wiley and Sons; Reproduced with permission [34]. Copyright 2021, Elsevier; Biomedical gadgets, for example, polymer‐based microneedles for drug delivery and photothermal wound dressing. Reproduced with permission [35]. Copyright 2024, John Wiley and Sons []; Reproduced with permission [36]. Copyright 2024, Elsevier; Layers to optimize the sensitivity of artificial eardrum or to improve the flexibility of the implantable heart pacemaker. Reproduced with Creative Commons CC BY license [37]. Copyright 2025, American Association for the Advancement of Science. Reproduced with permission [38]. Copyright 2025, American Chemical Society. Smart stimuli‐responsive polymers with inherent piezo‐coupling effects, flexibility, and biocompatibility are one of the most promising candidates for self‐powered sensors and biomedical gadgets.

Stimuli‐responsive smart polymers play a central role in enabling these piezo‐coupling effects, owing to their ability to dynamically respond to external stimuli and mediate charge generation and transfer across diverse transduction mechanisms. Polymers such as polyvinylidene fluoride (PVDF), poly‐L‐lactic acid (PLLA), and their composites exhibit multiple transduction behaviors, including piezoelectric, pyroelectric, and triboelectric effects. In this review, five main coupling effects were studied, which were piezo‐phototronic, piezo‐triboelectric, piezo‐pyroelectric, piezo‐pyro‐phototronic, and pyro‐phototronic [27, 28, 29, 30, 31]. Moreover, the polymers possess inherent flexibility, permeability, multi‐role, performance, sensitivity, and biocompatibility make them ideal candidates for wearable and implantable devices. Therefore, these materials are used in many gadgets, such as wearable self‐powered sensors [15, 32], implantable sensors for different organs [33, 34], biocompatible drug delivery systems and wound dressing patches [35, 36], and supporting layers to maximize the wearability for artificial parts/heart pacemakers [37, 38](Figure 1). However, their pristine output is inferior compared to their bulk semiconductor counterparts. Therefore, through strategic molecular design and structural engineering, the crystalline‐amorphous interface within polymers can be tailored to enhance the piezoelectric and pyroelectric outputs [19, 39, 40]. Additionally, post‐processing techniques such as annealing and uniaxial stretching can improve the dipole mobility, thereby improving the piezoelectric and pyroelectric responses [39, 41]. Another effective strategy for enhancing stimuli‐responsive mechanisms is the hybridization of functional nanofillers within a polymer matrix, such as liquid metals, transition metal dichalcogenides (TMDs), and ferroelectric materials [42, 43]. Furthermore, diverse device morphologies (e.g., fibrous vs. film) not only improve sensing performance due to increased surface area and porosity but also offer enhanced permeability for better user comfort [44, 45]. The biocompatibility of some polymers is also noteworthy, as it enables prolonged and safe wear with minimum toxicity to the skin or internal organs [46, 47, 48]. Other characteristics, such as self‐healing ability, ultra‐stretchability, and temperature/humidity resistance, have also been incorporated to enhance the durability and reliability of these multifunctional polymers [20, 49, 50, 51, 52, 53, 54, 55].

The integration of piezo‐coupling effects with smart polymers enables the development of ultrasensitive, self‐powered sensors for various applications. These include real‐time physiological monitoring, where enhanced sensitivity and wearability allow sensors to be comfortably attached to various body parts for precise movement tracking. The location of the sensor on the body is a critical factor in selecting the appropriate piezo‐coupling mechanism. On the other hand, substantial research focus has been devoted to the development of piezo‐coupling effects for applications such as wound healing, drug delivery, and human‐machine interfaces. Owing to their inherent flexibility, biocompatibility, and high detection range, these devices can be implanted into internal organs to detect subtle biochemical signals, monitor recovery processes, or release drugs in response to specific stimuli [15, 27, 28, 29, 33, 34, 35, 36]. Importantly, the electrical signals generated by these piezo‐coupling effects can serve both as sensing outputs and as intrinsic power sources, eliminating the need for external batteries and enabling long‐term autonomous operation. Additionally, the “self‐powered” devices could be categorized into two distinct paradigms, which are the completely autonomous sensing devices and the ones that could only provide signals. The first type could provide sufficient energy for every step, from signal acquisition then processing to data transmission. Conversely, the second type only has the capability to convert and collect signals, whilst data processing and transmission are undertaken by external circuits. Most piezo‐coupling devices belong to the second class as piezo‐coupling effects are often highly sensitive but have poor energy‐harvesting output. Nevertheless, there is research about fully closed‐loop self‐powered devices which operate based on piezo‐triboelectric effect [28, 56, 57, 58].

This review highlights recent advances in piezo‐coupling effects in smart polymers, enabling the development of flexible, self‐powered sensors with enhanced sensitivity for biomedical applications. The review begins by outlining the fundamental principles of some sensing mechanisms, followed by an in‐depth discussion of functional stimuli‐responsive polymeric materials, their performance characteristics, and fabrication strategies. It then explores the design and implementation of ultrasensitive, self‐powered sensors (both wearable and implantable) and their integration into biomedical applications. Reflecting current technologies, this report emphasizes that most of these devices are partial self‐powered, rather than the fully closed‐loop counterparts. Applications such as physiological monitoring, wound healing, drug delivery, and human–machine interfaces receive special attention. Finally, the review addresses current challenges and future directions for scalable, high‐performance, and multifunctional polymer‐based piezo‐coupling sensing technologies.

## 2 Fundamentals of Physical Sensing Effects

In response to external stimuli, smart polymers integrated within appropriately engineered device architectures can generate electrical signals suitable for sensing applications. At the atomic level, any external stimuli applied to the materials would change their atomic lattice and electrical structure, producing detectable signals [59]. On the one hand, core effects like piezo‐, tribo‐, pyroelectric, and photovoltaic effects could convert mechanical, temperature gradients, and light into electricity. On the other hand, by combining piezoelectricity with other effects, the generated output will come from different mechanisms, resulting in higher sensitivity and versatility for wearable or implantable sensing devices.

### 2.1 Fundamental Effects

#### 2.1.1 Piezoelectric Effect

When a force is applied to a piezoelectric material, it will make the material's crystal lattice become asymmetric, displacing the charge centres, hence polarizing the material and creating electricity. This is known as the direct piezoelectric effect [60, 61]. Conversely, the inverse piezoelectric effect arises when an applied electric potential induces structural disorientation in anisotropic materials [62, 63, 64, 65]. The direct and inverse piezoelectric effects are quantitatively described by Equations (1) and (2), respectively.

(1) $$S=s^{E}T+dE\;$$

(2) $$D=dT+\varepsilon^{T}E$$

where *S* is strain, *s^E^* is the mechanical compliance at a constant electric field (*E*), *T* is the applied mechanical pressure, and *d* is the piezoelectric constant. For the inverse effect's equation, *D* stands for the electric displacement, while ε^*T*^ is the permittivity at constant mechanical force.

From these equations, it could be seen that the outputs of both the direct and inverse piezoelectric effects are dependent on the applied strain's magnitude. Furthermore, the ion distribution inside the crystal structure facilitates piezoelectricity. In the pristine state, there is an equilibrium between the positive and negative charges, and this stable condition makes the piezoelectric material remain electrically neutral. When a compressive pressure is applied, this equilibrium is disrupted, distributing asymmetric ions. This asymmetry generates electrical dipoles, which in turn produce a piezoelectric signal [66, 67, 68].

**FIGURE 2:**
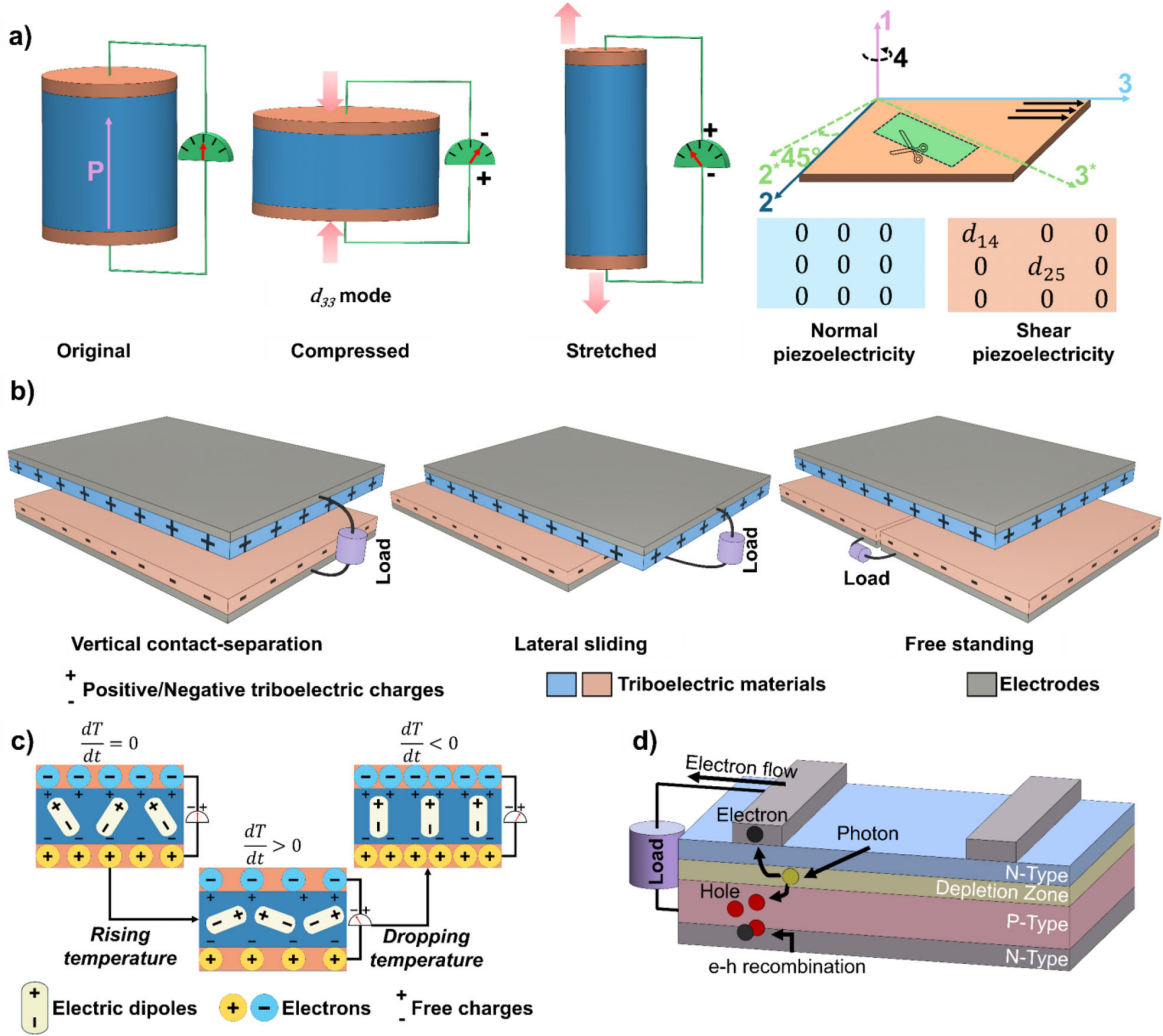
Working principles of different sensing effects. (a) Piezoelectric effect in compressive‐strain mode (*d_33_*) and shear stress mode (*d_14_*); (b) three operation modes of the triboelectric nanogenerators, from left to right: contact‐separation, lateral sliding, free‐standing; Operation principle of (c) pyroelectricity and (d) photovoltaic. The principles of physical sensing effects are the core foundations to understand how they could be combined, and electrical charges are generated inside the polymeric materials.

This theoretical framework has enormously influenced the application of piezoelectric materials for energy harvesting applications. By integrating electrodes on opposite faces of the piezoelectric material, the output signal could create an alternating current [69, 70, 71]. The electric generation principle of piezoelectric in *d_33_* (compressed‐strained) and *d_14_* (shear) is illustrated in Figure 2a.

#### 2.1.2 Triboelectric Effect

Like piezoelectric, the triboelectric effect also enables the conversion of mechanical into electrical energy. Due to the widespread ability of materials to undergo triboelectrification, triboelectricity is more accessible [72, 73, 74]. A basic triboelectric device consists of two materials with different electron affinities. There are three main working modes, which are contact‐separation, lateral sliding, and freestanding modes. Additionally, each mode has a corresponding single‐electrode variation that facilitates charge transfer and signal generation (Figure 2b) [75, 76, 77, 78, 79]. The first contact‐separation triboelectric nanogenerator (TENG) was invented by Fan et al., and this classic model will be used to explain the working principle [80]. When two materials with different electron affinities make contact under an external force, one will become positively charged, and the other will have negative charges. Once the force is released, the created charges will be divided, creating a potential difference between two electrodes connecting to each triboelectric layer, forming an electron flow between the electrodes throughout an external circuit. If this contact‐separation continues, an AC will result. Many materials tend to receive electrons, while others prefer to donate. Numerous studies led to the construction of a triboelectric series, which rates the electron affinities of various materials [81, 82]. The output of a TENG is often determined by open‐circuit voltage (*V*_OC_), which is calculated by the equations below.

(3) $$V_{\mathrm{OC}}=\frac{\sigma_{\mathrm{SC}}}{\varepsilon_{0}}x\left(t\right)\;$$

With σ_SC_ and *x*(*t*) as the surface charge density and distance between two triboelectric surfaces (this parameter varies with time). Meanwhile, the number of generated charges (*Q*_SC_) is calculated by

(4) $$Q_{\mathrm{SC}}=\sigma_{\mathrm{SC}\;}A\;$$

where *A* is the contact area of the triboelectric layers. Moreover, the equation for the short‐circuit current (*I*_SC_) is the time derivative of charges flowing across two electrodes via the external circuit.

(5) $$I_{\mathrm{SC}}=\frac{dQ_{\mathrm{SC}}}{dt}\;$$

Besides choosing the materials, the TENG's performance could be increased by varying the applying force, working distance, and contact‐separation frequency. Normally, the output is proportional to these parameters until the number of charges reaches their maximum value, from this threshold, increasing pressure, working distance/speed will not enhance the triboelectric output [83, 84, 85].

#### 2.1.3 Pyroelectric Effect

The pyroelectric effect relies on the temperature fluctuation in certain materials that possess spontaneous polarization (*P_s_*). *P_s_* produces surface charges on opposite faces of the pyroelectric material, with each surface bearing charges of opposite polarity. These polarized planes can attract free charges from the surrounding environment, thereby facilitating the generation of an electric signal in response to thermal variations. When the material is heated (*dT/dt*>0), electric dipoles vibrate more strongly than usual because of thermal vibration, decreasing not only *P*_s_ but also the number of free charges tied to the material. With a short circuit condition, there will be an electric current flowing between two polarized surfaces. For open circuit condition, an electric potential is yielded in the material because now the free charges stay on the electrode. When the material is cooled down (*dT/dt*<0), the electric dipoles’ orientation is stable; therefore, *P_s_* rises and the electric current flow is reversed under short circuit conditions since the polarized planes could attract free charges [6, 86, 87] (Figure 2c).

#### 2.1.4 Photovoltaic Effect

Directly converting sunlight into electricity has been a foundational strategy in the pursuit of renewable energy since the discovery of the photovoltaic (PV) effect in 1839 [26, 88, 89]. Photovoltaic technology operates on principles analogous to those of p–n junctions. Upon absorption of photons from sunlight, electrons in the photovoltaic material are excited from the valence band to the conduction band, resulting in the generation of electron‐hole pairs. These charge carriers are subsequently separated by the internal electric field present at the p–n junction, with electrons migrating toward the n‐type region and holes toward the p‐type region. In a closed circuit, this separation facilitates the flow of current, thereby enabling the generation of electricity (Figure 2d) [90, 91, 92]. To evaluate the current‐voltage parameters from a solar cell, the single diode equation was used:

(6) $$I=I_{\mathrm{ph}}-I_{0}\left(e^{\frac{q\left(V+IR_{s}\right)}{nkT}}-1\right)-\frac{V+IR_{s}}{R_{\mathrm{sh}}}$$

where:

*I*, *I*_ph_, and *I_0_*: output, photogenerated, and saturation current values
*q* = 1.602 × 10^−19^ C (elementary charge); *k* = 1.381 × 10^−23^ J/K (Boltzmann constant)
*V*: Terminal voltage across the cell
*R_s_* and *R_sh_*: series and shunt resistance
*n*: ideality factor
*T*: Kelvin temperature

### 2.2 Piezo‐Coupling Sensing Effects

Piezo‐coupling effects are an advanced step of fundamental effects, where the strain‐induced polarization from piezoelectricity is combined with other sensing mechanisms. This multimodal approach allows for the simultaneous acquisition of signals from diverse sources, which is particularly advantageous for detecting low‐intensity inputs and increasing performance for self‐powered sensors [93, 94, 95, 96]. In this section, synergistic piezo‐coupling mechanisms are introduced and discussed.

#### 2.2.1 Piezo‐Phototronic Effect

**FIGURE 3:**
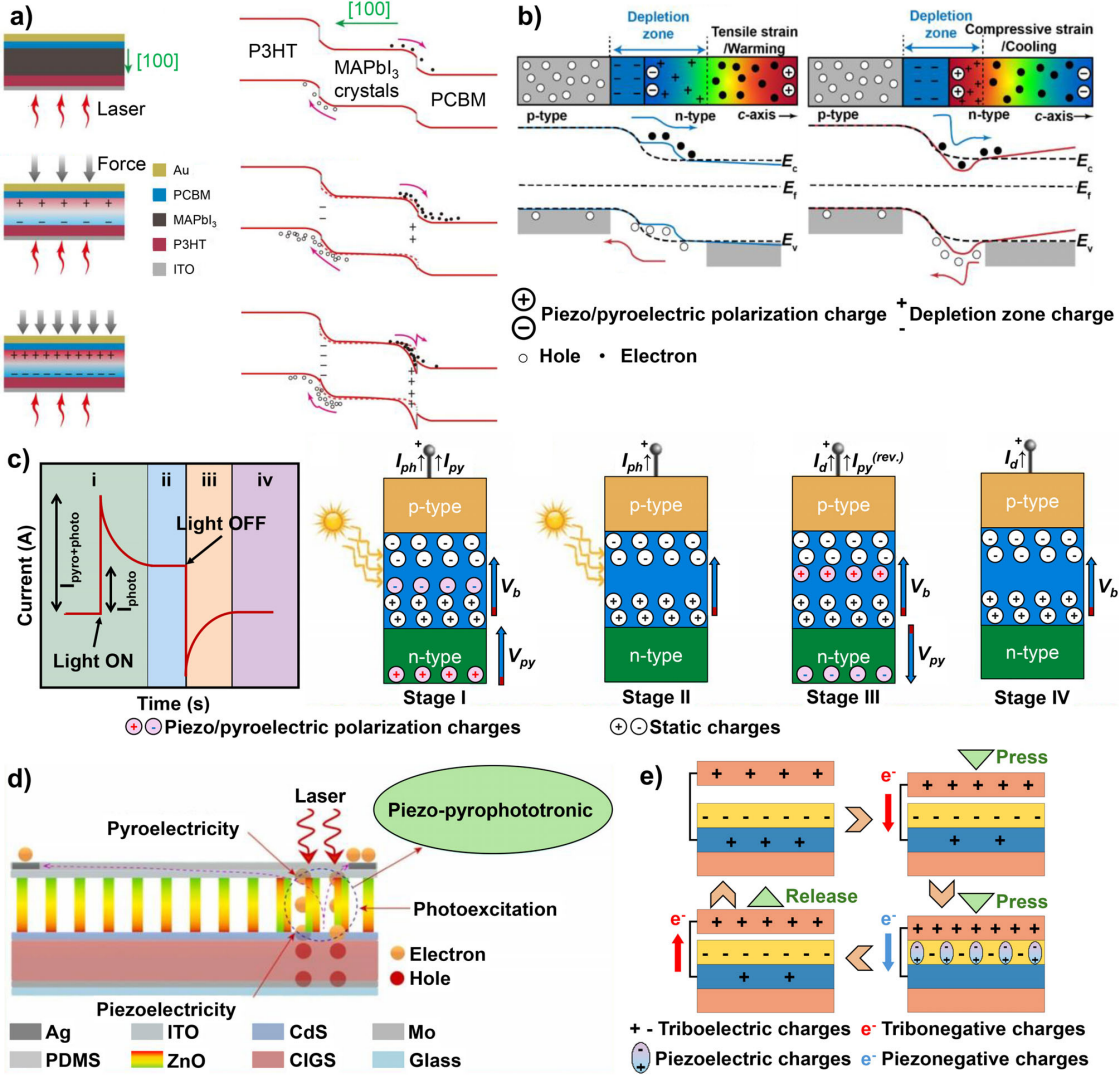
Charges generation and migration of common piezo‐coupling effects. (a) Illustration of the piezo‐phototronic effect in MAPbI_3_‐P3HT:PCBM composite. Reproduced with permission [99]. Copyright 2018, American Chemical Society; (b) Schematic diagram of how electrons, holes, and charges move in piezo‐pyroelectric effect. Reproduced with permission [30]. Copyright 2023, Elsevier; (c) Current signal obtained from the pyro‐phototronic effect, divided into four stages. Reproduced with Creative Commons CC BY license [98]. Copyright 2025, Elsevier; (d) Schematic illustration of the piezo‐pyro‐phototronic sensor based on the monolithic CIGS heterojunction. Reproduced with permission [102]. Copyright 2024, Elsevier; (e) Working principle of the piezo‐triboelectric effect. Reproduced with permission [107]. Copyright 2022, John Wiley and Sons. The piezo‐coupling effects could alter the band structures and charge flows, raising the sensitivity and versatility.

The piezo‐phototronic effect is the coupling between the piezoelectric effect and photoexcitation in non‐centrosymmetric semiconductors. Basically, when tensile or compressive strain is applied, a piezoelectric potential is generated, leading to the formation of piezoelectric charges on the surface and corresponding potentials at the interfaces within the semiconductor. These changes modify the energy band structure of the junction to regulate the charge carrier creation, separation/recombination, and transportation of optoelectronic processes, thereby enhancing the performance of optoelectronic and sensing devices. For instance, assuming a junction is formed between a metal and an n‐type semiconductor, with tensile strain, the charges are positively polarized to bring more free electrons at the junction, lowering the barrier height and promoting electron transfer. In contrast, under compressive strain, the negatively polarized charges repel electrons, thus raising the barrier and decreasing the electron migration. Similar principles could be observed for p‐n junction. When the n‐type semiconductor has a piezoelectric characteristic while the p‐type does not, with tensile strain applied to the n‐type material, a positive piezopotential is formed and attracts some photoinduced electrons, which decrease the energy band and prevent carrier movement. Conversely, the compressive strain repels electrons due to the negative polarization but favors the photoinduced charges by increasing the band edge (Figure 3a) [27, 97, 98, 99].

#### 2.2.2 Piezo‐Pyroelectric Effect

Piezo‐pyroelectric coupling effect arises in non‐centrosymmetric crystals that exhibit spontaneous polarization. This interaction occurs when temperature‐induced variations in polarization (pyroelectric effect) influence the piezoelectric response of the material, and vice versa, resulting in a synergistic enhancement of both effects. Upon temperature change, positive and negative electric charges are displaced, resulting in spontaneous polarization. Consequently, a potential drop is created to form an electron flow from the bottom to the top electrode. With the open circuit, this pyroelectric potential is measurable. Meanwhile, if an external pressure is applied on the electrode/pyroelectric material/electrode structure, spontaneous polarization fluctuations will occur due to the noncentrosymmetric crystalline configuration. Therefore, under simultaneous application of heat and pressure, piezo‐pyroelectric phenomenon interacts to augment charge flow and increase multiple detections, creating intensive multisignals with enhanced precision and magnitude (Figure 3b) [30, 100, 101].

#### 2.2.3 Pyro‐Phototronic Effect

Similar to the piezo‐phototronic, the pyro‐phototronic effect represents the coupling between pyroelectricity and photoexcitation, wherein the efficiency of the pyroelectric response is enhanced by incident light. The mechanism behind pyro‐phototronicity could be explained using a current‐time graph from the pyro‐phototronic behavior of a p‐n junction material. At Stage I, under rapid light illumination, the charge carriers are generated and drift quickly, forming an extremely sharp current increase. A rapid temperature increase of the n‐type material due to abrupt irradiation causes a light‐induced pyroelectric field (*V_py_*) by sequentially creating electrons (*e^−^*) and holes (*h^+^*) near the p‐type and n‐type junctions, respectively. The polarized charges further split and drift the photogenerated electron‐hole pairs, resulting in a transitory pyroelectric current (*I_py_*). When the light source is steady, the temperature fluctuations are negligible, thus stabilizing *dT/dt*, during this period, the output current is solely from light excitation (*I_ph_*) (Stage II). Turning off the light causes a temperature drop (*dT/dt* < 0), the electrons (*e^−^*) and holes (*h^+^*) are situated at n‐ and p‐junctions, respectively, resulting in an opposing dark current (*I_py’_*) (Stage III). Subsequently, *I_py’_* diminishes when the temperature remains unchanged (*dT/dt = 0*) and the current is also zero since the light had already been off (Stage IV) (Figure 3c).

#### 2.2.4 Piezo‐Pyro‐Phototronic Effect

The piezo‐pyro‐phototronic effect emerges from the interplay between piezoelectric, pyroelectric, and photoexcitation, all of which are governed by the crystal lattice's response to external stimuli. In this integrated mechanism, piezoelectric and pyroelectric phenomena contribute to the generation of electric charges, while photoexcitation introduces additional charge carriers. The synergistic combination of these effects significantly enhances the sensitivity and performance of optoelectronic and sensing devices [98]. Liu et al. described this tri‐coupling effect in their research; when a laser is irradiated onto the CIGS/CdS heterojunction, charge carriers appear due to photoexcitation. A lateral photovoltage (LPV) is also created from a photosensitive gradient due to the laser non‐uniformity. If an external pressure and a temperature gradient are applied at the same time, piezo/pyroelectric polarization charges and fields are induced at the ZnO/CdS interface. Consequently, the LPV is more sensitive and accurate toward mechanic forces and the lateral photovoltaic effect (LPE) becomes optimal (Figure 3d) [102].

#### 2.2.5 Piezo‐Triboelectric Effect

The coupling of piezoelectric and triboelectric effects offers a promising strategy to further enhance energy output, despite both effects being fundamentally driven by repetitive mechanical pressure. When triboelectricity is generated by the electron affinities between the triboelectric materials, the piezoelectric effect arises from the inherent characteristic of specific non‐centrosymmetric materials to produce an electric charge when subjected to mechanical stress. Once united, a synergistic enhancement of the total electrical output will be formed [103, 104, 105, 106]. Interestingly, theoretical calculations and research indicate that the coupling is not solely additive; the interaction between the two effects might yield a total output that surpasses the sum of the separate contributions. The asymmetric triboelectric response dominates the electric output at low acceleration and low frequency when the materials are in the contact‐release state (Figure 3e) [107]. Conversely, the symmetric intrinsic piezoelectric response prevails under high acceleration and high frequency, where substantial deformation occurs in the solid fibers. To further enhance the coupling output, porous structures and crystals are employed [103, 108, 109, 110, 111].

## 3 Polymeric Devices Fabrication Strategies

To fabricate an effective sensing platform, the bulk polymers (powder, pellets) are often dissolved into solutions before undergoing different manufacturing techniques, such as spin/dip coating, electrohydrodynamic, or inkjet/screen printing [112]. Collectively referred to as solution‐processing technologies, these methods enable the rapid, uniform, and scalable transformation of polymeric solutions into functional solid films. These methods are widely applied to produce sensing components with optimal performance, high flexibility, and durability.

**FIGURE 4:**
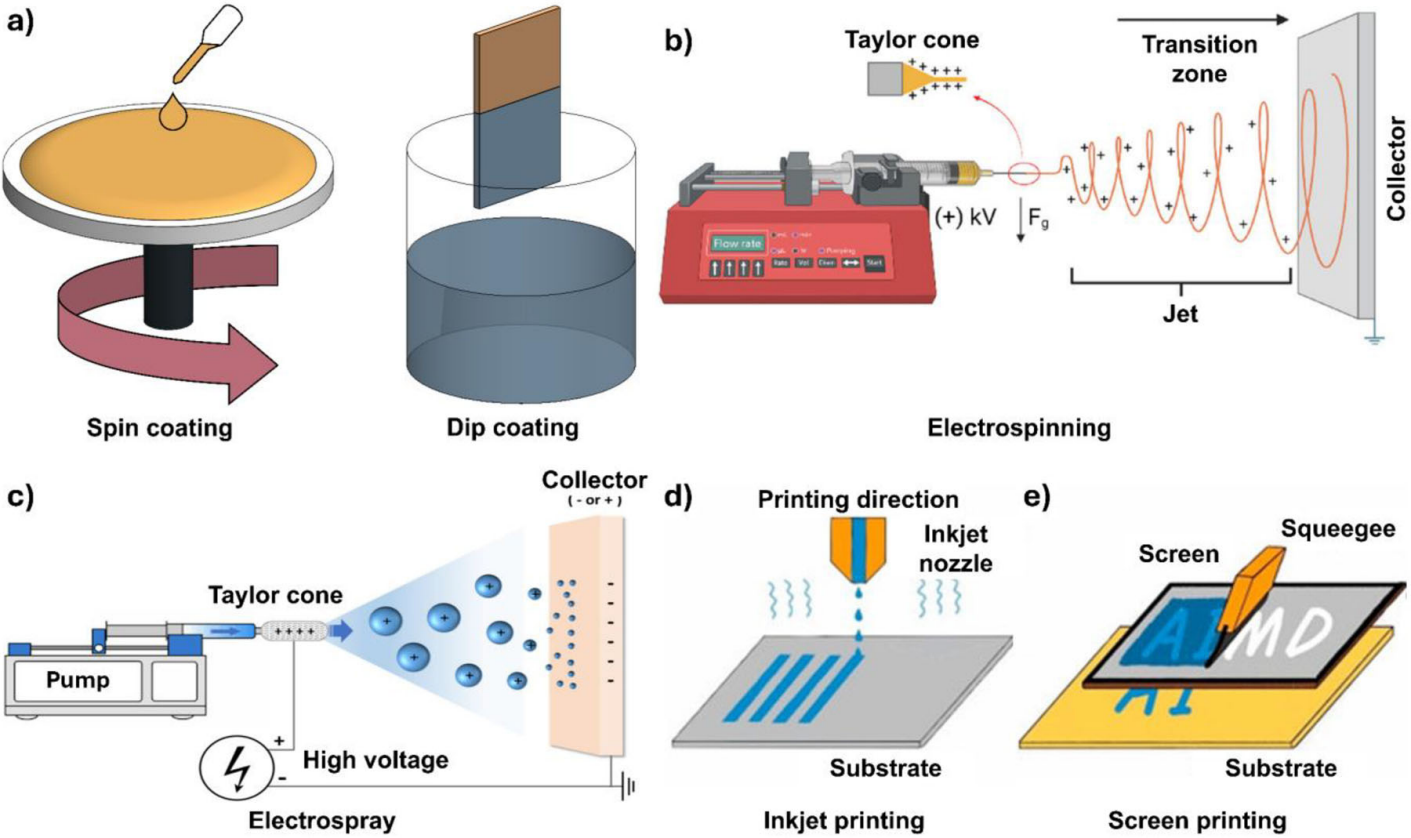
Some common fabrication techniques for the polymeric, stimuli‐responsive layers for piezo‐coupling sensors: (a) Spin coating and dip coating; (b) Electrospinning. Reproduced with Creative Commons CC BY license [113]. Copyright 2023, John Wiley and Sons; (c) Electrospray. Reproduced with permission [114]. Copyright 2024, American Chemical Society; (d) Inkjet printing and (e) screen printing. Reproduced with permission [115]. Copyright 2020, American Chemical Society. Solution‐processing techniques, including coating, electrohydrodynamic, and printings, play a major role in defining the morphology, crystalline phase, and dipole orientation of the piezo‐coupling polymers, which hugely contribute to the overall sensing capability.

Coating is one of the most effective methods to fabricate a morphologically uniform and homogeneous thin film. In spin coating, a substrate is initially secured by centrifugal force, then a solution droplet is applied onto the substrate before being accelerated to a designated rotational speed. Due to the angular velocity, the droplet is spread evenly across the substrate surface. Parameters such as rotational speed, solution viscosity, solvent choice, and polymer molecular weight can be adjusted to control film thickness. This technique is versatile and affordable, as it could produce thin films with ultra‐thin, uniform thickness and high mechanical strength, which are favourable for wearable sensors. This method is hugely beneficial for many polymer types; for instance, it could improve the transformation from amorphous α‐phase into piezoelectric β‐phase of PVDF and its derivatives. PLLA with inherent shear piezoelectricity also takes advantages from the shear rates and centrifugal force from spin coating [116]. Additionally, the performance of thin‐film transistors made from conjugated semiconducting polymers (P3HT, PTAA) relies on spin coating parameters since they might affect these polymers’ solid‐state order and morphology [117, 118, 119]. Dip‐coating, in contrast, involves immersing the substrate in a polymer solution, resulting in the formation of a solid film upon drying [120, 121]. For dip‐coating, it entails immersing the substrate in a polymer solution, resulting in the formation of a solid film upon drying. Factors that significantly influence the coatings include the viscosity and the processing temperature. For optimal uniformity, the immersion and withdrawal processes should be conducted at a carefully regulated speed (Figure 4a) [122, 123]. Dip‐coating was applied in the fabrication of conductive polymer hydrogels, especially PEDOT:PSS hydrogels, since this technique could either create interlocked percolating networks or electrical pathways within the hydrogels [124, 125, 126]. Furthermore, dip‐coating was also helpful in forming a functional coating layer outside commercial textiles, thus equipping them with sensing capabilities. For example, MXene or carbon nanotubes (CNTs) could establish a percolating network on fabrics via hydrogen bonds, converting them into wearable sensors [127, 128].

Electrohydrodynamic techniques encompass both electrospinning and electrospray, each utilizing a high‐voltage electric field to transform polymeric solutions into micro/nano fibers or particles [129, 130]. On the one hand, electrospinning fabricates nonwoven fibers with high surface area‐to‐volume ratios and porous structure. Parameters such as viscosity, electric field amplitude, flow rate, and nozzle sizes are the key elements in determining the fiber diameter, while solvent evaporation, temperature, and humidity might impact the fiber morphology [131, 132]. Since many polymeric material classes could be electrospun, it is most widely applied for fluoropolymers, because the mechanical stretching and the high voltage field encourage the formation of β‐phase and dipole alignment. Besides, most polymers have their own triboelectricity; many commercial polymers (polyurethane‐PU, nylon 66, etc.) were electrospun and produced extraordinary sensing performance, as electrospinning fabricated multilayered, porous configurations [133, 134]. However, conductive polymers like PEDOT:PSS or polypyrrole (PPy) require careful electrospinning optimization due to the electrical interaction between the polymer solution and the high voltage field [135, 136]. On the other hand, electrospray deposits the solution in the particle form, and the decisive elements to modify the particle characteristics are similar to electrospinning (Figure 4b,c) [137, 138]. This approach is more feasible for polymers that solidify quickly after leaving the spraying nozzle, such as poly(vinyl alcohol) (PVA), poly(vinylpyrrolidone) (PVP), or gelatin. Nevertheless, electrospray often plays the role of depositing extra coating onto the main sensing layer; the electrosprayed nanoparticles could reach and attach uniformly to complicated surfaces.

Printing techniques offer a rapid and scalable approach for fabricating large‐area thin films, with enhanced controllability enabled by programmable printing patterns and customizable masks. Inkjet printing employs thermal or piezoelectric techniques to selectively spray and deposit electronic ink onto a substrate. It does not require masking but still provides high‐resolution thin films. Aerosol jet printing involves the generation of aerosols, consisting of droplets with diameters ranging from 2 to 5 µm, via pneumatic or ultrasonic atomization methods. Subsequently, those aerosol droplets are transported by inert gas and deposited onto the substrate to form high‐resolution patterns. The thin film quality is significantly influenced by ink composition, drop size, resolution, printing speed, and layering (Figure 4d) [139, 140]. The suitable polymeric materials for this technique should have the Ohnesorge value (Z) between 1 and 10, which represents the ink's viscosity and surface, the materials should have sufficient viscosity to flow and not drip. Conjugated polymers (PEDOT:PSS, P3HT, electrochromic polymers) are well‐known candidates; they are chosen due to their tuneable rheology, water dispersibility, and printing stability. Moreover, crosslinking polymers (acrylates, methacrylates, SU‐8, PDMS) are also appropriate for inkjet printing, since they will remain viscous enough for printing until being cured [117, 141]. Meanwhile, screen printing plays a vital role in the industry due to its cost‐effectiveness, mass production capacity, and facile post‐treatment process. Ink is applied to the substrate by pushing it through a patterned screen plate using a squeegee (Figure 4e). The quality/complexity of the screen mask, mesh fineness, ink viscosity, substrate wetting properties, squeegee pressure, and printing speed are the essential factors to fabricate functional, uniform, and defect‐free sensing layers [142, 143]. In comparison to inkjet, the screen‐printing polymers could be much more viscous, composed of larger particles; elastomeric polymers (urethanes, polyesters) are employed as the protective matrix in stretchable composites. Due to high stretchability and resistance, the screen‐printed polymers can protect the conductive fillers against environmental conditions [144, 145]. 3D printing enables the fabrication of complex geometries through a layer‐by‐layer additive manufacturing process. A key advantage of this technique is the ability to pre‐program intricate designs prior to fabrication, distinguishing it from conventional manufacturing methods. Additionally, 3D printing eliminates the need for molds or jigs, thereby significantly reducing production time. Common 3D printing techniques include material extrusion, material jetting, and stereolithography, each of which requires specific parameters and material conditions, such as physical state, viscosity, and processing temperature [146, 147]. Shape memory polymers like TPU and PLA are commercially available, and they could act as strain and temperature sensors, respectively. Some conductive composites/hydrogels could also be 3D printed for light and temperature sensors [148]. Besides the main fabrication techniques, other methods are exploited to improve the performance/wearability of the main functional layers. Plasma treatment allows adhesion between layers, therefore increasing the contact area for maximum charge transportation from the sensing element to the external circuit. Gas selection/flow rate and plasma power are adjusted to get the desirable adhesion. Moreover, surface engineering is used to improve the contact area of the thin films [149, 150]. Corona discharge occurs when the air is ionized by a high electric field. The free electrons are accelerated, and ions are created by the electric field; interestingly, the ions flow (corona current) creates a space charge region. The electrode geometry, applied voltage, humidity, and surface conditions affect hugely on the corona discharge efficiency [151, 152, 153].

## 4 Piezo‐Coupling Polymeric Materials

Polymers exhibiting piezo‐coupling effects hold significant promise for developing ultrasensitive sensors, owing to their inherent flexibility, high performance, and fabrication simplicity. The most common piezoelectric polymer is PVDF, followed by its derivations because they also possess pyroelectric and triboelectric properties. The elastic nature of responsive polymers is particularly advantageous for wearable electronic applications. Furthermore, their composition is tuneable, enhancing their versatile and suitability across a broad range of applications. The ability to modify chemical concentrations also enables diverse synthesis techniques and the incorporation of various functional additives to improve performance. This section provides an overview of fabrication methods and a comprehensive summary of polymers exhibiting piezoelectric coupling effects.

### 4.1 Piezoelectric Polymers

#### 4.1.1 PVDF and Derivations

**FIGURE 5:**
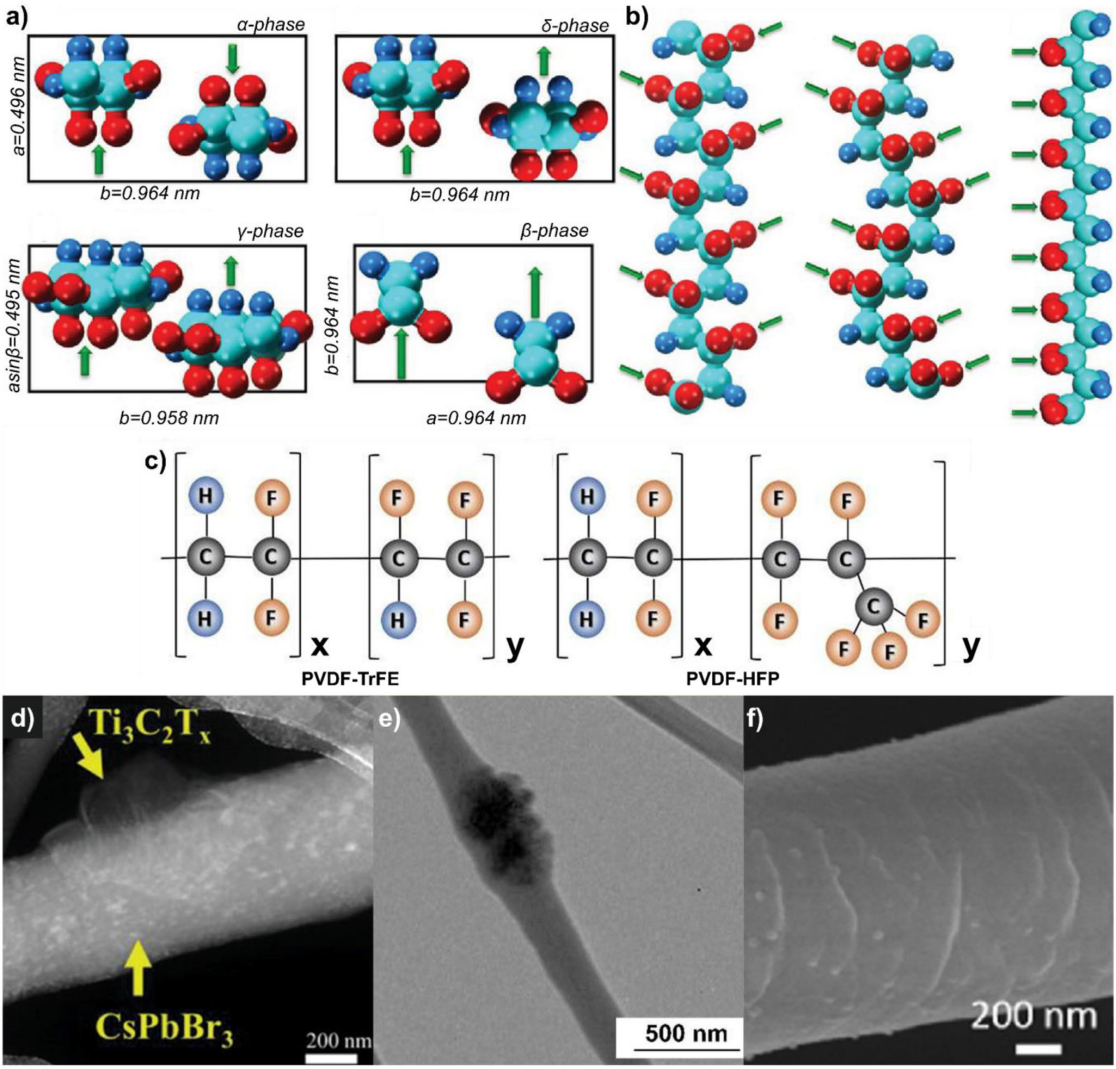
(a) The individual unit cell of PVDF in α‐, δ‐, γ‐, and β‐phases; (b) Schematic illustrations of PVDF chain conformations: TGTG’, TTTG^+^TTTG^−^, TTTT (fluorine, carbon, and hydrogen atoms are represented by red, cyan, and blue orbs). Reproduced with permission [155]. Copyright 2012, American Chemical Society; (c) 2D chemical structure of PVDF‐TrFE and PVDF‐HFP. Reproduced with Creative Commons CC BY license [161]. Copyright 2023, American Chemical Society; (d) TEM images of PVDF/CsPbBr_3_/Ti_3_C_2_T*_x_* fiber in the dark field. Reproduced with Creative Commons CC BY license [163]. Copyright 2023, John Wiley and Sons; (e) TEM image of a nanofiber mat showing the agglomerated BF33BT nanoparticles within the PVDF matrix. Reproduced with permission [164]. Copyright 2022, American Chemical Society; (f) SEM image of the KNN‐ZS/PVDF nanocomposite electrospun web. Reproduced with permission [165]. Copyright 2022, Elsevier. PVDF and its co‐polymers, known for their inherent electroactive β‐phase and flexibility, are the most popular choice for flexible piezo‐coupling sensors.

Polyvinylidene fluoride (PVDF) is a widely known piezoelectric polymer due to its remarkable elasticity, resilience to mechanical/chemical resistance, and thermal stability. It is a semicrystalline polymer that forms a radial spherulitic structure, with the backbone oriented approximately perpendicular to the plane. It comprises repeating units of (CH_2_–CF_2_) with 2.6 Å. The dipole moment in PVDF arises from the electronegative fluorine (δ−) and the electropositive hydrogen (δ+), and only in the β‐phase, this dipole moment is perpendicular to the polymer chains, while the dipole moment in other phases is not [154, 155]. PVDF can crystallize into various phases, namely α, β, γ, δ, and ε, depending on the processing conditions (Figure 5a) [61, 155, 156]. Among them, the first two are the most common for piezoelectric applications, with the α‐phase is the most thermodynamically stable while the β‐phase exhibits the highest electroactive properties, including piezoelectricity, pyroelectricity, and ferroelectricity. It is structured in orthorhombic unit cells (similar to α‐phase) with *Cm2m* space group. Here, the polymer chains adopt a planar zigzag (all‐trans) conformation, with dipolar moments aligned parallel to the crystallographic b‐axis. The δ‐phase is a polar variant of the α‐phase, achieved by applying a high electric field, which inverts the dipole moments along the field direction. Its chain conformation resembles that of the α‐phase. Moreover, the γ‐phase of PVDF exhibits an intermediate conformation between the β‐ and α‐phases (TTTG^+^TTTG^–^). The β‐phase repeat unit has a dipolar moment and polarization of 7 × 10^−28^ cm C and 131 mC/m^2^, respectively, this phase has a rational, all‐trans conformation (TTTT) (Figure 5b). The ε‐phase, which is challenging to obtain, has a similar geometry to the γ‐phase. Consequently, the α‐ and ε‐phases are nonpolar, while the β‐, γ‐, and δ‐phases are polar. The PVDF's glass transition temperature (*T_g_*) is −35°C, while the melting temperature is within the range from 160°C to 190°C, and the crystallinity varies between 35% aand 60%. About the electrical properties, due to the existence of fluoride in the chemical structure, PVDF has a high electronegativity. As mentioned above, the β‐phase has the highest dielectric constant because of its better polarity. Besides, another important factor influencing the dielectric properties of PVDF is the interface between its amorphous and crystalline regions. Additionally, the oriented amorphous fraction (OAF), which links the mobile amorphous fraction to the lamellar crystal, plays a role in ferroelectric switching and enhances the β‐phase, dielectric, and ferroelectric properties of PVDF. OAF has a similar chain orientation to a liquid crystalline polymer, but it does not have a defined crystalline structure. Moreover, the piezoelectric performance could be improved when the VDF dipoles reorient in the OAF, creating the electrostriction effect [157].

However, PVDF has some limitations, which are low dielectric and piezoelectric constant (*d_33_*). Hence, other PVDF‐based copolymers were introduced to enhance the piezoelectric performance. They facilitate the direct formation of the polymer in its electroactive phase when processed from the melting solution. Additionally, these copolymers enhance and tailor the degree of crystallinity and electroactive response to meet specific requirements [158, 159, 160]. Trifluoroethylene (TrFE) possesses a chemically resemble structure to VDF, whereas one hydrogen atom is replaced by a fluorine atom (Figure 5c, left) [154, 161]. If the TrFE content is under 20%, the resulting PVDF‐TrFE will have multiple phases. In contrast, when the TrFE concentration is above 20%, the molecular chains transition to the all‐trans (TTTT) arrangement from the TGTG′ conformation, characteristic of the β phase, regardless of the processing conditions, favouring the piezoelectric output [162]. Another highly regarded derivative is poly(vinylidene fluoride‐co‐hexafluoropropylene) (PVDF‐HFP), which is PVDF merging with an amorphous HFP (Figure 5c, right) [161]. Similar to PVDF‐TrFE, it also has a high β‐phase conformation, and among the PVDF copolymers, PVDF‐HFP owns the most remarkable *d*_31_ coefficient of about 21 pC/N [161].

To maximize the inherent piezoelectricity of PVDF, many fillers were added inside this polymer. For instance, Xue et al. employed CsPbBr_3_–Ti_3_C_2_T*_x_* heterojunctions in PVDF fibers, both CsPbBr_3_ and Ti_3_C_2_T_x_ were uniformly dispersed along the fibers. The PVDF/CsPbBr_3_/Ti_3_C_2_T_x_ composite provided a power density value of 3.8 µA/cm^2^ (Figure 5d) [163]. However, CsPbBr_3_ contains toxic lead; therefore, other fillers were investigated. Muduli et al. introduced PVDF‐BF33BT composite nanofiber mat with a high power density value of 142 mW/m^2^ (Figure 5e) [164]. Zn‐ and Sn‐doped potassium sodium niobate (KNN‐ZS) is another promising candidate since it is lead‐free and possesses a synergistic piezo‐triboelectric effect. The PVDF/KNN‐ZS nanofibrous web reported by Banerjee et al. could generate up to 25 V (Figure 5f) [165].

**FIGURE 6:**
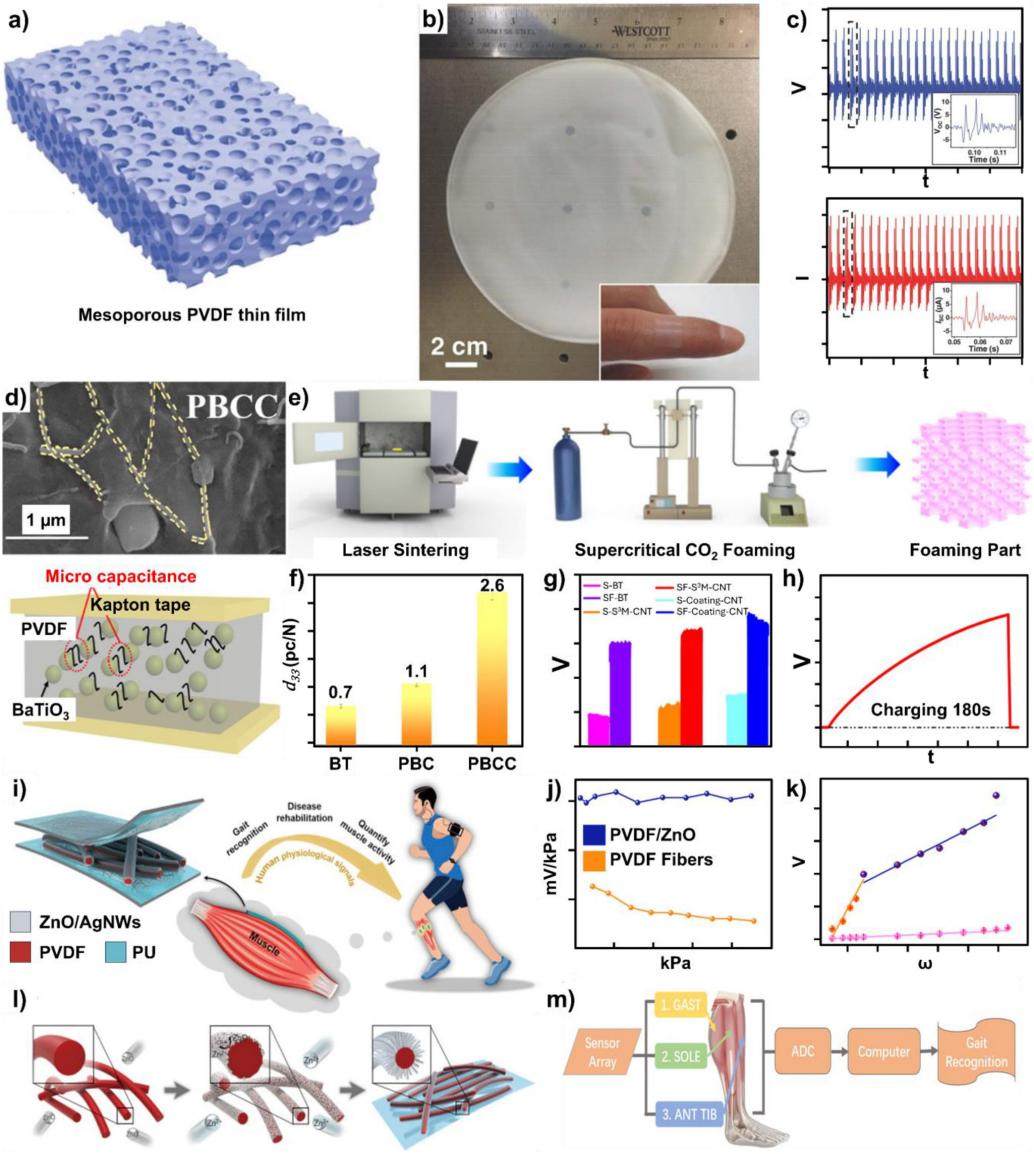
(a) Schematic illustration of the mesoporous PVDF thin film; (b) Digital image of the wafer‐sized mesoporous PVDF thin film (inset: attachment of a small mesoporous PVDF piece to the finger); (c) The voltage (blue) and current (red) performance of the PVDF nanogenerator under continuous oscillation (inset: output signal of an individual oscillation cycle). Reproduced with permission [166]. Copyright 2014, John Wiley and Sons; (d) Top row: SEM images of the CNT‐coated PVDF/BaTiO_3_ powders (PBCC), bottom row: Illustration of BaTiO_3_ and CNT inside printed PBCC device; (e) Schematic process for the fabrication of PBCC with porous structure via Sc‐CO_2_ foaming method; (f) *d_33_* coefficient of PBCC; (g) Output voltage of different composites; (h) The relationship between potential and time when the SF‐Coating‐CNT charged a 1 µF capacitor. Reproduced with permission [167]. Copyright 2021, American Chemical Society; (i) The schematic diagram of the 3D interlocked PVDF/ZnO fibers‐based PME adhering to the calf muscle to detect muscle movement; (j) The sensitivity comparison between pristine PVDF and PVDF/ZnO fibers; (k) Dependence of the *V*_OC_ on varying curvature (ω) from 0.0242 mm^−1^ to 0.2962 mm^−1^ (blue, orange: PVDF/ZnO; pink: PVDF fibers); (l) Synthesizing process of the core–shell PVDF/ZnO nanofibers; (m) Schematic diagram of the gait recognition system by gathering analog piezoelectric signals from the sensor array and converting into digital signal for storage and analysis. Reproduced with permission [168]. Copyright 2020, Elsevier. Modifying the PVDF structures and integrating nanofillers successfully enhance the β‐phase for better piezoelectric output.

In 2014, Mao et al. introduced a mesoporous PVDF film with high β‐phase concentration; by adjusting the film's porosity, this parameter is proportional to the flexibility and softness, enabling direct adhesion to human skin or electrical devices while efficiently converting ambient mechanical vibrations into energy (Figure 6a,b). A standard piezoelectric nanogenerator (PENG) sized 20 mm × 1 mm could deliver average peak voltage and current values of up to 11 V and 9.8 µA, respectively (Figure 6c). Moreover, many NGs can be seamlessly incorporated into a single system to augment its output without the need for rectifying or synchronizing separate signals [166]. To further increase the piezoelectric output, ferroelectric polycrystalline ceramics, such as barium titanate (BaTiO_3_), were widely used because they could act as a nucleating agent to enhance the ratio of β‐phase PVDF. Yang et al. invented PVDF/BaTiO_3_ powders with carbon nanotube (CNT) coating for selective laser sintering (SLS) 3D printing. Meanwhile, the macroscopic array configuration is an exceptional 3D structure for enhancing piezoelectric output capacity (Figure 6d). Furthermore, supercritical CO_2_ (Sc‐CO_2_) foaming postprocessing yielded bionic balsa wood structure foams, demonstrating an elevated voltage output of 19.3 V, capable of charging a 1 µF capacitor to 5.03 V within 180 s (Figure 6e,g,h). The PVDF/BaTiO_3_/CNT featuring a microscopic 3D isolation network resulted in an increase in the composite material's *d_33_* from 0.7 to 2.6 pc/N (Figure 6f) [167]. Conversely, to enhance the wearability of PENG, electrospinning PVDF fibers was developed rapidly, as it also facilitates the phase transition from α to β. Additionally, this technique can straight away produce a piezoelectric fibrous mat without the necessity for supplementary poling to align dipoles and ferroelectric domains [103]. Yang et al. developed a three‐dimensional hierarchically interlocked piezoelectric sensor from PVDF/ZnO composite by epitaxially growing ZnO nanorods on the surface of electrospun PVDF nanofibers, providing flexible and highly permeable fiber‐based physiological monitoring electronics (PME; Figure 6i). The synergistic piezoelectric effect of the interlocked ZnO nanorods and the oriented PVDF nanofibers with a high electroactive phase has significantly enhanced the sensitivities of the piezoelectric materials in both pressing and bending modes, increasing them by 6 times and 41 times, respectively, compared to pristine PVDF fibers (Figure 6j,k). Figure 6l shows how the PVDF/ZnO core–shell fibers were fabricated. Consequently, the engineered PME can accurately identify the intricately nuanced physiological signals of respiration, wrist pulse, and muscular activity. Furthermore, a sensitive gait identification system was effectively constructed utilizing PME arrays (Figure 6m). This device offers an alternate method for monitoring subtle human physiological signals and shows significant promise for broader applications in healthcare and clinical diagnosis [168].

**FIGURE 7:**
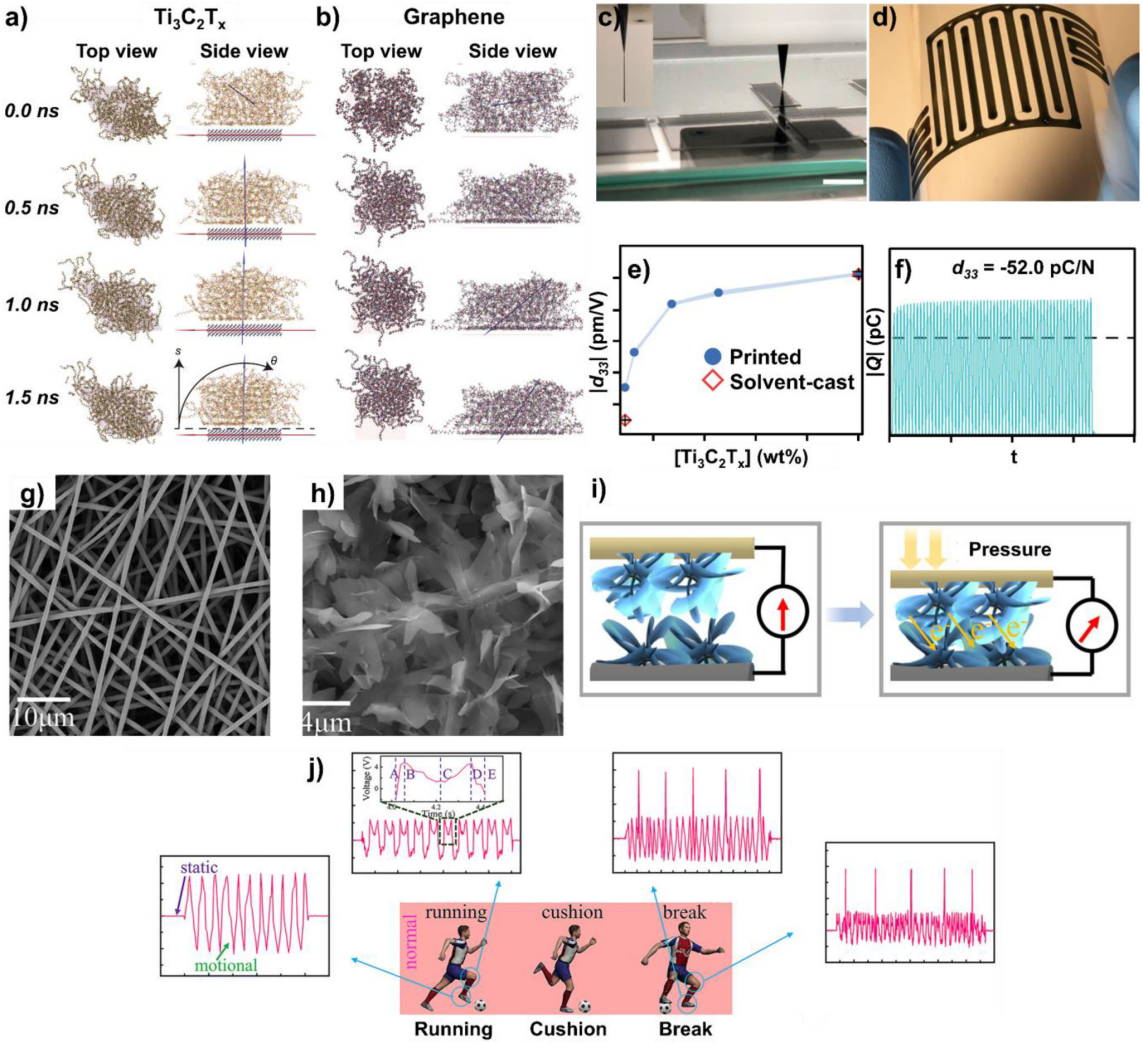
(a) The top view and side view of PVDF‐TrFE with Ti_3_C_2_T_x_. In the side view snapshots, the polarization vectors of the polymers and the substrates are blue and red arrows, respectively; (b) Identical analysis with graphene chosen as the substrate; (c) Digital photographs capturing the SEA extrusion printing process with the Ti_3_C_2_T_x_/PVDF‐TrFE ink (0.50 wt%) (scale bar 10 mm). The inset displays a self‐sustaining single filament; (d) Image of the Ti_3_C_2_T_x_/PVDF‐TrFE (0.50 wt%) printed a digitalized pattern onto the poly(ethylene terephthalate) (PET) substrate; (e) The effective piezoelectric charge coefficient (*d_33_*) was calculated from the piezoelectric force microscopy (PFM) data, which includes solvent‐cast PVDF‐TrFE and Ti_3_C_2_T_x_/PVDF‐TrFE (0.50 wt%) films used as controls. (f) The generated surface charge of Ti_3_C_2_T_x_/PVDF‐TrFE (0.50 wt%) was measured over a period of 60 compression cycles at 2 Hz. The dashed line indicates the charge produced by a fully polarized PVDF‐TrFE co‐polymer. Reproduced with Creative Commons CC BY license [170]. Copyright 2021, Springer Nature; SEM images of (g) PVDF‐HFP electrospun fibers (Magnification: ×2000) and (h) PVDF‐HFP/ZnO composite membrane (Magnification: ×5000); (i) Piezoelectric mechanism of the PVDF‐HFP/ZnO fibers; (j) Motion tracking tests on a soccer player's knee and ankle. Reproduced with permission [171]. Copyright 2022, Springer Nature. Many functional additives (Mxene, ZnO, etc.) were added to the PVDF‐based polymers to further increase the electrostatic interaction and dipole alignment, bringing efficient flexible PEGs for motion recognition and limb rehabilitation.

Recently, efforts have been made to research PVDF‐TrFE with the TrFE concentration varying from 20% to 50% since their Curie temperature (*T_C_*) ranges between 52°C and 78°C, while the *T_m_* is about 152°C, leading to the tremendous improvement of crystallinity by annealing the copolymer between the *T_C_* and *T_m_* [169]. Shepelin et al. revealed that Ti_3_C_2_T_x_ (Mxene) nanosheets exhibit a robust electrostatic interaction with the PVDF‐TrFE, leading to the development of a fixed polarization in the fluoropolymer, oriented perpendicular to the basal plane of the Mxene nanosheet after only 0.5 ns. This effect is not evident when employing graphene nanosheets (Figure 7a,b). The distinctive 2D geometry of Mxene nanosheets allows for the translation of induced local net polarization into macroscale polarization through either solvent‐evaporation‐assisted (SEA) extrusion printing or solvent casting. The SEA extrusion technique could produce monolayer thin films using a nozzle that has an inner diameter of 200 µm (Figure 7c). Additionally, this method could also fabricate complicated patterns on flexible substrates, as displayed in Figure 7d. About the piezoelectric evaluation, the *d_33_* of the Ti_3_C_2_T*_x_*/PVDF‐TrFE (0.50 wt.%) SEA extrusion‐printed film increased up to a maximum of −5.11 pm/V, an ultimate improvement of 926% over the solvent‐cast PVDF‐TrFE co‐polymer film. Moreover, the above‐mentioned composite achieved an outstanding *d_33_* of −52.0 pC/N at macroscale measurements without electrical poling (Figure 7e,f). The powerful electrostatic interactions between the nanofiller and fluoropolymer produced a physically resilient and flexible piezoelectric generator (PEG) without performance deterioration [170]. In 2022, hierarchical PVDF‐HFP/ZnO electrospun fibers were developed by Li et al., whereas the enhanced output came from the interaction between the electric dipole within the PVDF‐HFP fibers and the asymmetric lattice of ZnO. About the morphology, PVDF‐HFP fibers have a smooth surface with an average diameter of 800 nm, whilst ZnO nanosheets grew densely on PVDF‐HFP fibers, and they have a petal‐like shape (Figure 7g,h). This composite also has a similar working principle to other PENGs, initially, electrospun PVDF‐HFP fibers will experience extrusion deformation upon application of an external force. Prior to deformation, the electric dipoles within the fiber are systematically aligned, exhibiting a stable polarization trend, which does not produce any electric signal. Upon compression and deformation, the configuration of the electric dipoles alters, leading to a reduction in the internal polarization intensity and consequently generating an electrical signal. ZnO nanosheets influence the piezoelectric characteristics of the sensor. They also envelop the surface of the PVDF‐HFP fiber; hence, when the sensor is subjected to impact, significant deformation and a robust piezoelectric potential arise between the ZnO nanosheets due to mutual extrusion (Figure 7i). With its high sensitivity and flexibility, this sensor was employed to precisely detect the imperceptible changes in a soccer player's motions to minimize ankle and knee injuries. The athlete's movement in those body parts created piezoelectric signals, which primarily rely on the variation in the joint's bending angle when the player performs different motions. Varied bending angles result in distinct forces on the pressure sensor, causing it to generate disparate piezoelectric signals (Figure 7j). Moreover, a Bluetooth Low Energy device monitors the soccer player's workout and wirelessly transfers the data to a smartphone application. The study provides a practical strategy for high‐precision detecting and safety monitoring in the sectors of medical, rehabilitation medicine, and workout security [171].

#### 4.1.2 Poly(L‐Lactic Acid)

**FIGURE 8:**
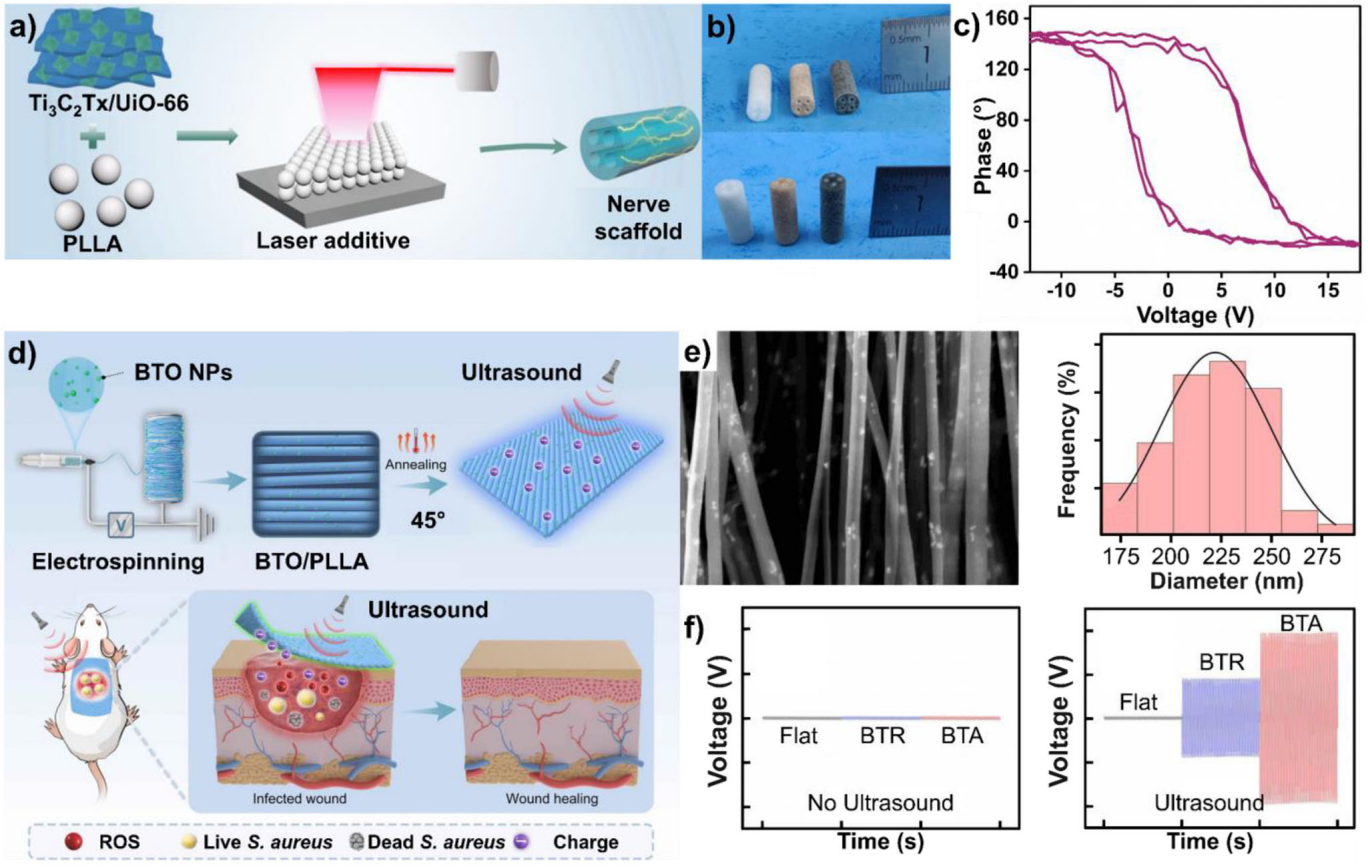
(a) Visualization of the nerve scaffold fabrication with PLLA via laser additive manufacturing; (b) Digital images of the nerve scaffolds made from PLLA, UIO‐66‐PLLA, and Ti_3_C_2_T*_x_*/UIO‐66‐PLLA (from left to right); (c) The piezorespone phase‐voltage curves of the nerve scaffold. Reproduced with permission [179]. Copyright 2025, American Chemical Society; (d) Following annealing and cutting at 45°, the aligned EFDs composed of BaTiO_3_‐doped PLLA can exhibit a superior piezoelectric effect when exposed to ultrasonic radiation. And upon application to a full‐thickness infected murine skin wound model in conjunction with ultrasound therapy, the EFD successfully facilitated the healing of open wounds infected with *S. aureus* by releasing ROS in reaction to ultrasonic stimulation; (e) SEM images of aligned BaTiO_3_/PLLA EFD with average diameter quantification; (f) Piezoelectric output of the BaTiO_3_/PLLA EFDs in flat film, random fibers (BTR) and aligned fibers (BTA) without and with ultrasound. Reproduced with permission [180]. Copyright 2024, American Chemical Society. PLLA is an up‐and‐coming polymer containing piezo‐coupling effect, and due to its biocompatibility and biodegradability, it can be further applied for biomedical gadgets.

Due to natural biodegradability and biocompatibility, PLLA has been attracting interest as a potential candidate for wearable sensors. Moreover, PLLA exhibits decent piezoelectricity because of the C═O dipoles present in the crystalline region and the alignment of the molecular chains. This biodegradable polymer has three phases, which are α, β, and γ. Among them, the α‐phase is obtained by melting and solution‐phase crystallization, it is the most stable with the 10_3_ helical chain configuration and pseudo‐orthorhombic cell. However, this phase has weak piezoelectricity because the C═O dipoles are arranged in every direction; therefore, their polarizations autonomously cancel each other and hardly create a dipole moment [172]. On the other hand, monitoring the drawing and annealing parameters will crystallize β‐phase PLLA that has C═O dipoles aligning along the backbone chain, which increases the polarization and produces more piezoelectric potential [173]. About the conformation, the β‐phase is a 3_1_ helical chain with a triclinic/orthorhombic unit cell. The γ‐phase could be acquired by growing PLLA epitaxially on a hexamethylbenzene substrate [174, 175]. Instead of *d_33_* like PVDF, PLLA possesses shear piezoelectricity (*d_14_*); under shear stress, the dipoles are rotated and polarized, generating electric charges. In the film structure, *d_14_* ≈ 10 pC/N, which is far lower than *d_33_* (20‐30 pC/N); this could be due to the high α‐phase composition, low crystallinity, high amorphous content, and intrinsic molecular structure [172, 176]. However, there are several methods to improve PLLA's piezoelectric output. One common strategy is adding fillers into the PLLA solution during synthesis; for example, BaTiO_3_ can boost the PLLA's charge generation, formation of β‐phase, and electrical polarization pathway facilitation [176, 177]. On the other hand, varying the PLLA fabrication methods would arrange the crystalline phase in order, such as melt spinning, casting, electrospinning, and post‐processing. Besides improving β‐phase and regulating the crystalline/amorphous ratio, these techniques are capable of aligning PLLA's backbone and C═O dipoles [172, 174, 176, 178]. Recently, Ti_3_C_2_T_x_ Nanosheet/UIO‐66 MOF was mentioned as another piezoelectric additive for PLLA, with the work of Ding et al., a self‐powered nerve scaffold is created using piezoelectric filler Ti_3_C_2_T_x_/UiO‐66 with PLLA. Ti_3_C_2_T_x_ nanosheets speed up the charge transfer, while UiO‐66 nanoparticles can produce localized piezoelectric potential when stimulated by ultrasound, and this composite could be simply attained by self‐assembling. The Ti_3_C_2_T_x_/UIO‐66‐PLLA piezoelectric multichannel neural scaffold was fabricated with laser additive manufacturing technology (Figure 8a). The scaffold's multilayer architecture was critical for allowing the proximal neurite to develop toward the distal end. The scaffold's diameter was approximately 0.5 mm (Figure 8b). The piezoelectric phase of the scaffolds showed both low and high regions, as depicted in Figure 8c [179]. On the other hand, BaTiO_3_ is also capable of boosting the piezoelectricity of PLLA; for example, Wu et al. employed this ceramic and aligned electrospinning simultaneously to create a piezoelectric fibrous dressing utilizing controlled ultrasound stimulation to enhance healing in an infected wound model. By the aligned electrospinning method, BaTiO_3_/PLLA electrospun fibrous dressings (EFDs) were made with an oriented order, which significantly benefited negative charges and electrets for enhanced charge storage stability (Figure 8d‐first row). After fabrication, the sample was attached to a fully infected rat skin; the EFDs exhibited bactericidal effectiveness through ultrasound‐mechanical‐electric conversion, leading to the generation of reactive oxygen species (ROS) to eradicate *S*. *aureus* inside the wound (Figure 8d, second row). In Figure 8e, SEM photos pointed out that the EFDs fibers had high smoothness with uniform diameters and homogeneous dispersion of BaTiO_3_ nanoparticles (NPs). From this morphological record, the calculated mean diameters of the aligned BaTiO_3_‐doped electrospun fibrous dressing (BTA) were 225.81 ± 36.96 nm. Regarding how ultrasonication affected the piezoelectric output of the composite, voltage comparisons between EFDs in flat film, random BaTiO_3_‐doped electrospun fibrous dressing (BTR), and BTA were evaluated. In general, all samples exhibited superior performance under ultrasonication compared to their efficiency without it (Figure 8f) [180].

#### 4.1.3 Natural Polymers

**FIGURE 9:**
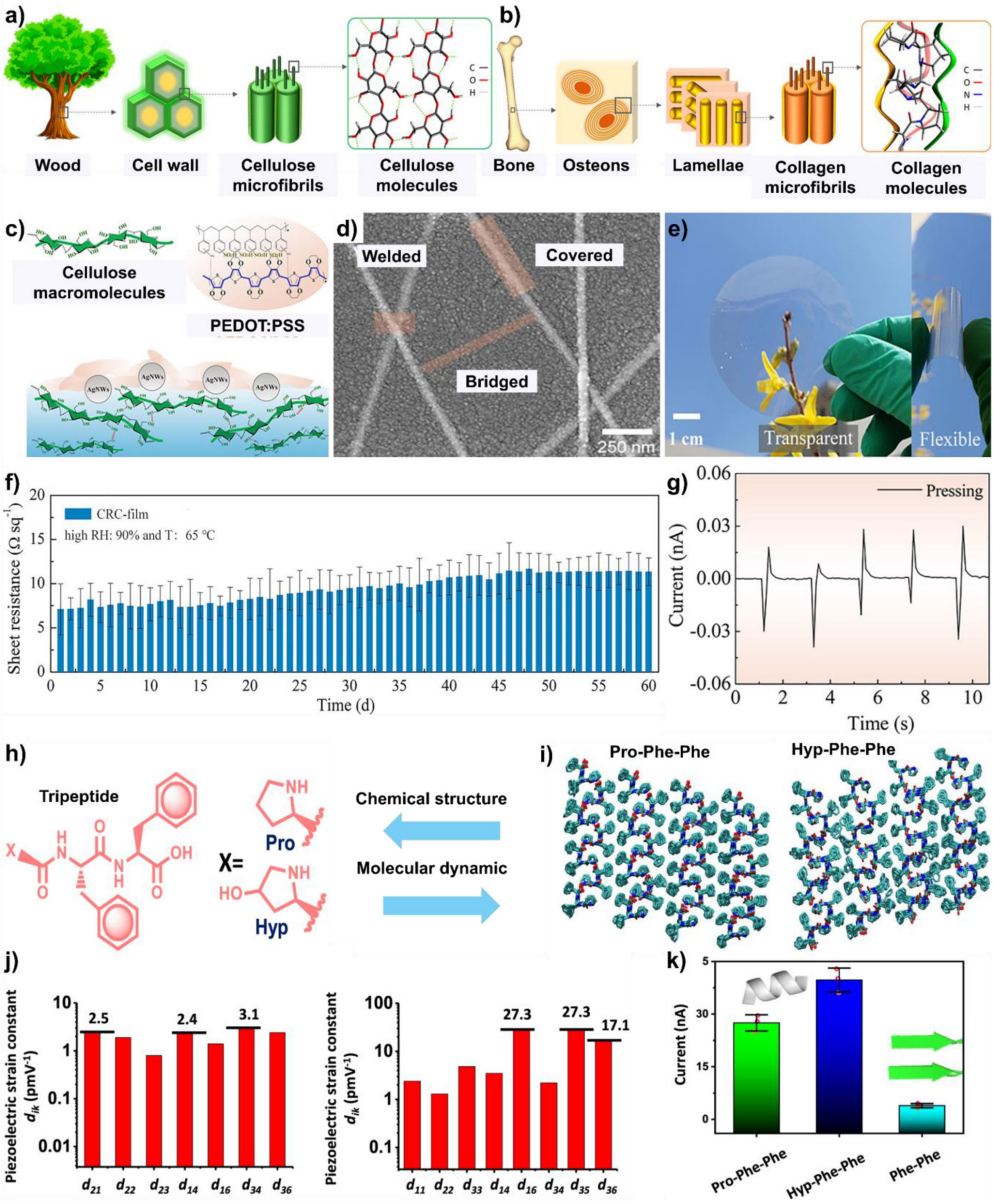
Structural hierarchy of (a) cellulose and (b) collagen. The polar hydrogen bonds in cellulose molecules are represented by the green dashes. For collagen, the polypeptide chains are arranged in three helices. One of them is shown with sticks and a semitransparent ribbon, while the other two are shown with ribbons alone. Reproduced with permission [181]. Copyright 2018, American Chemical Society; CRC‐film's (c) chemical structures and (d) SEM images; (e) Digital photos of the fabricated CRC‐film with high transparency and good flexibility; (f) Sheet resistance of the CRC‐film throughout 60 days at 90% RH and 65°C; (g) Current piezoelectric response of the CRC‐film after being exposed for 30 days. Reproduced with permission [188]. Copyright 2020, Elsevier; (h) Chemical structures of Pro‐Phe‐Phe and Hyp‐Phe‐Phe tripeptides; i) Computed supramolecular structures of Pro‐Phe‐Phe and Hyp‐Phe‐Phe under ambient temperature dynamics after 0.1 µs; (j) Different piezoelectric strain constants (*d_ik_*) Pro‐Phe‐Phe and Hyp‐Phe‐Phe tripeptides; (k) Output comparison among Pro‐Phe‐Phe, Hyp‐Phe‐Phe and helical Phe‐Phe. Reproduced with Creative Commons CC BY license [191]. Copyright 2021, Springer Nature. The non‐centrosymmetric crystalline structures and polarized groups, cellulose, and collagen have piezoelectricity, facilitating the fabrication of biocompatible sensors that can work flawlessly without causing damage to the subjects.

To further exploit the biocompatibility for in vivo experiments, cellulose and collagen, which are essential constituents in the hierarchical architecture of plant cell walls and animal bone tissues (Figure 9a,b) [181]. The dipole moment from hydroxyl groups in cellulose and amino acid groups in collagen indicates that both materials exhibit piezoelectric capabilities [182, 183]. The noncentrosymmetric arrangement of cellulose and collagen molecules in crystals can provide a net dipole moment [184, 185]. Therefore, this part examines why piezoelectricity is derived from cellulose and collagen, as well as some recent findings about them.

About cellulose, it is a linear polysaccharide composed of repeating glucose units, and its inherent crystalline structure with hydrogen bonding among the polysaccharide chains confers piezoelectric capabilities [186]. Notably, besides the polar hydroxyl functional groups, cellulose fibers’ alignment also has an important effect on the piezoelectric response. Under a favorable stress direction, the generated electric signal will be higher [187]. Wang et al. reported an approach for interfacial assembly and encapsulation to create flexible, transparent, and conductivity‐stable films. Silver nanowires (AgNWs) are encased between a regenerated cellulose film, serving as a flexible substrate, and poly(3,4‐ethylenedioxythiophene)‐poly(styrene sulfonate) (PEDOT:PSS) nanosheets, functioning as a sealing coat, due to the synergistic effects of coordination complexation and hydrogen bonding [188]. The resultant conductive regenerated cellulose‐based film (CRC film) possesses a resilient interfacial structure and characteristics (Figure 9c). Throughout the SEM photo, it could be observed that the CRC sample had a rough surface due to the high density of PEDOT:PSS distributed across cellulose and AgNWs (Figure 9d). About the overall morphology, as shown in Figure 9e, the CRC film had extremely high transparency and flexibility. In terms of performance, even when exposed to severe conditions, including a high‐moisture environment of 90% relative humidity (RH) and a temperature of 65°C for up to 60 days, the film maintains a high and stable conductivity of about 11.3 Ω/sq (Figure 9f). From these advantages, the authors applied the materials for a flexible, transparent, and biocompatible strain‐based piezoelectric sensor; although it was exposed to 90% RH for 30 days, the current output was still maintained (Figure 9g) [188]. On the other hand, because of the semicrystalline molecular structure (for instance, the triple‐helix structure), collagen also has piezoelectricity. Under external pressure, the cellulose chains will be oriented and tend to generate a dipole moment. Moreover, these chains contain polar hydroxyl groups and produce electric charge once a mechanical force is applied on them [189, 190]. Understanding these properties, Bera et al. introduced a peptide‐based piezoelectric generator employing an innovative helical configuration of Phe‐Phe‐derived peptides, which were self‐assembling tripeptides Pro‐Phe‐Phe and Hyp‐Phe‐Phe (Figure 9h), only utilizing proteinogenic amino acids [191]. To investigate the assemblies in further depth, molecular dynamics (MD) simulations were conducted under the following conditions: using a nanocrystal of each peptide submerged in an extensive water box to simulate bulk solvation, constant room temperature and air pressure, and maintaining a physiological pH of 7.4. The key discovery is that the constriction of the hydrogen bond network in the Hyp‐ variation correlates with enhanced conformational flexibility in the Phe‐Phe rings, illustrating the equilibrium between electrostatic and π–π interactions in the development of helical assemblies (Figure 9i). With this enhanced characteristic, Hyp‐Phe‐Phe collagen had better piezoelectric coefficients than the Pro‐Phe‐Phe counterpart. In the case of Pro‐Phe‐Phe, low charge tensor components reaching 56 mC/m^2^ yield moderate *d_ij_* values of up to 3.1 pm/V. In Hyp‐Phe‐Phe, hydroxylation reduces the symmetry of the unit cell from monoclinic to triclinic, resulting in an increase in the number of non‐zero piezoelectric constants within each tensor. Hydroxylation results in a large increase in the maximum charge tensor value (*e_33_* = 0.1 C/m^2^). The rise in e*_ij_* values and the decline in c*_ij_* values result in a notable enhancement in the predicted piezoelectric strain constants, specifically *d_35_* = −27.3 pm/V and *d_33_* = 4.8 pm/V (Figure 9j). These piezoelectric coefficients provided similar current output, under the identical applied pressure of 23 N, the Pro‐Phe‐Phe configuration provided approximately 30 nA, whilst the Hyp‐Phe‐Phe counterpart generated nearly 40 nA (Figure 9k). Moreover, the performance and key advances of the common piezoelectric polymers and their composites were listed in Table 1.

**TABLE 1:** Comparison among different piezoelectric polymers, in terms of output and key advantages.

Piezoelectric polymers/composites	Sample size (cm^2^)	*d_33_* (pC/N or pm/V)	Power density (µW cm^−2^)	Applications	Reference
PVDF	5 × 5	12	0.81	Motion sensor	[192]
PVDF‐TrFE	6.3 × 0.3	24	16.4	Energy harvester	[193]
PVDF/BTO	5 × 5	82	3.64	Gait detection	[192]
PVDF‐TrFE/SWCNTs	3 × 1	33.4	1.85	Wearable sensor	[194]
PLLA	2 × 1.5	3	0.07	Bio e‐skin	[195]
PLLA/BaTi_2_O_5_	2 × 2	3.1	15.60	Energy harvester	[196]
PVDF/Cellulose	1.5 × 1.5	31.6	3.51	Pressure sensor	[197]
PVA/CNT@Cellulose	1 × 3	16	7.5	Strain sensor	[198]
Collagen blocks	1.2 × 1.2	4.8	—	Biocompatible NG	[191]

### 4.2 Triboelectric Polymers

Unlike piezoelectric, triboelectric materials are diverse due to their working mechanism, which relies on contact electrification and electrostatic induction between two distinct materials. This phenomenon is not confined to crystal structures or chemical compositions. A wide range of materials, including common polymers, fabrics, metals, and biological materials, can demonstrate triboelectric behavior. A difference in electron affinity between the two materials is the sole requirement, a property present in nearly all substances to varying extents. In a triboelectric couple, one material typically receives electrons while the other tends to give them away. Among the plethora of triboelectric materials, many polymers were either chosen as the electron‐donating or ‐withdrawing layers.

#### 4.2.1 PTFE

**FIGURE 10:**
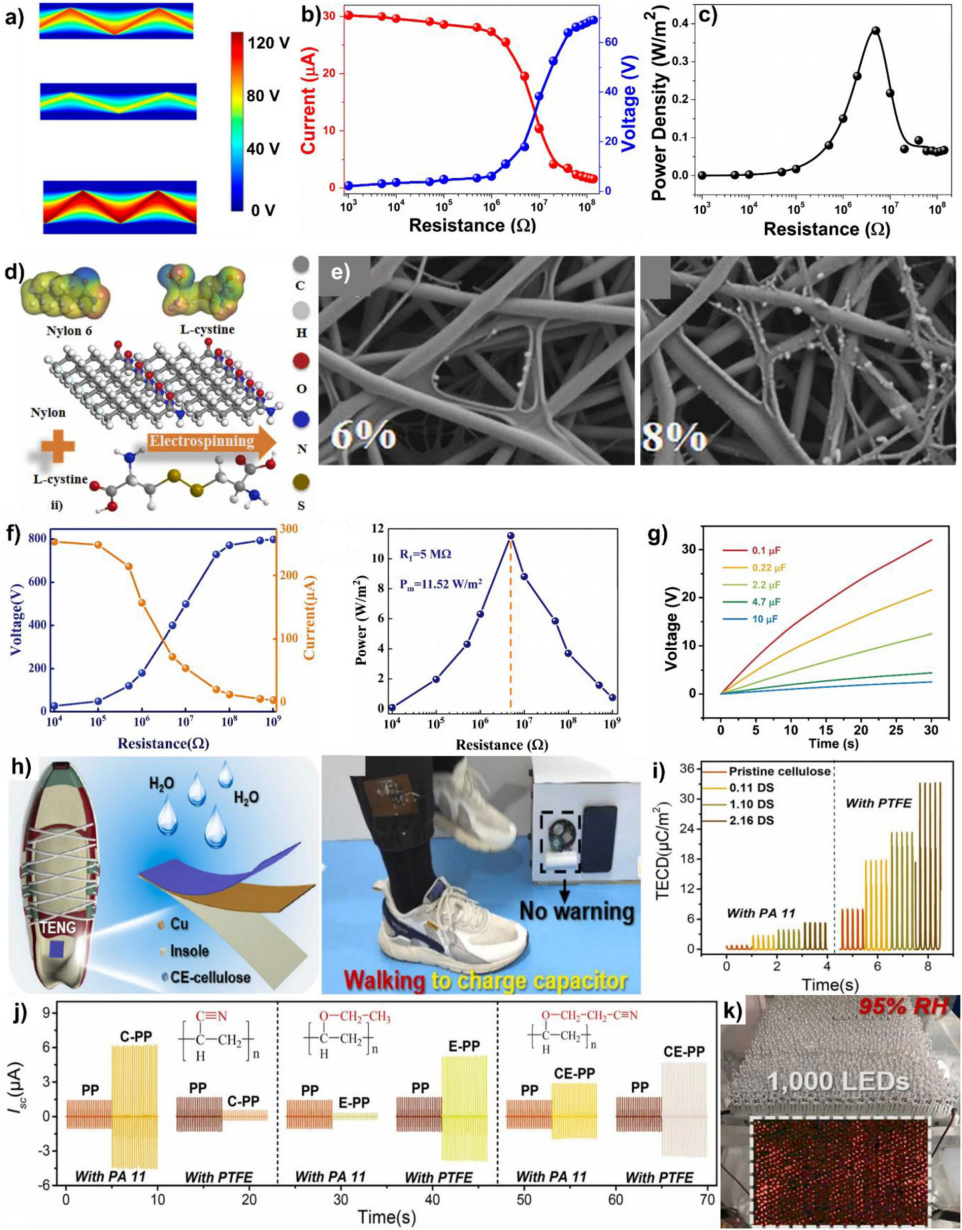
(a) Finite‐element simulation of the TENG composed with nanopillar PTFE during working; (b) Output voltage, current, and (c) power density dependence on load resistance. Reproduced with permission [204]. Copyright 2014, American Chemical Society; (d) Computed chemical structures of nylon 6 and l‐cystine; (e) SEM images of NCNF with 6 wt% and 8 wt% l‐cystine fibers; (f) Analysis of voltage, current, and peak power density in a 5‐layer HM‐TENG across different resistances; (g) The charging performance of the 5‐layer HM‐TENG with different capacitors in 30 s. Reproduced with permission [209]. Copyright 2023, Elsevier; (h) The CE‐cellulose TENG's practical application; (i) TECD comparisons among CE‐TENGs with different DS values; (j) The influence of the cyano (C‐PP), ether (E‐PP), and cyanoethyl (CE‐PP) on the electrical output of TENGs; (k) CE‐cellulose‐based TENG powered 1000 LEDs at 95% RH. Reproduced with permission [214]. Copyright 2022, Elsevier. Polymeric triboelectric materials could be categorized into three groups, which are electron‐withdrawing (PVDF, PTFE, FEP); electron‐donating (nylons, PU), and dual‐polarity (cellulose); most of them could be optimized to achieve highly efficient triboelectric output.

Fluorine exhibits a strong ability to withdraw electrons because it is the most electronegative element (Pauling electronegativity = 4.0). Therefore, it has a significant propensity to attract electrons during the formation of chemical bonds. Fluorinated polymers exhibit these characteristics due to the presence of fluorine along their backbone, which enables them to attract and retain electrons. This results in a superior negative affinity and an increased tendency to collect electrons from other materials. Meanwhile, fluorine is among the smallest atoms (van der Waals radius, *r_w_* = 1.35), resulting in its positive nucleus being in close proximity to the bonding electrons, thereby enhancing its capacity to attract them [199]. Moreover, the bond between fluorine and other atoms exhibits high polarity. This establishes a significant dipole and amplifies the electron‐withdrawing effect. Fluorine's high electronegativity and low polarizability result in a strong retention of attracted electrons, thereby enhancing the stability of its negative charge and creating an electron‐deficient state in the remainder of the molecule [200]. Polytetrafluoroethylene (Teflon/PTFE) is rated as one of the most tribonegative materials in the triboelectric series [81, 201]. Apart from containing fluoride, this polymer is hydrophobic and chemically inert, allowing it to provide a stable triboelectric output during a long duration under harsh environmental conditions [202, 203]. Additionally, it has a high surface charge density, and it could even be improved by many techniques. For instance, the classic article of Wen et al. presented a TENG featuring a wavy‐structured Cu–Kapton–Cu configuration positioned between two flat nanostructured PTFE films. This structural design enabled the TENG to self‐restoratively recover post‐impact without additional springs, while also facilitating the conversion of direct impact into lateral sliding. The nonlinear characteristics of the wavy core's stiffness enabled the TENG to effectively harvest mechanical energy across a wide frequency spectrum. The working principle of this device was illustrated carefully: upon impact, the acrylic substrate compressed the wavy core vertically while extending horizontally, thereby transforming the vertical compressive force into lateral friction between the core and both PTFE surfaces. The contact area between the PTFE and Cu film was increased, resulting in a reduced average distance between the two materials. Upon removal of the impact, the wavy core will retract horizontally and extend vertically, resulting in lateral friction between the core and both PTFE surfaces, generating charges, as simulated in Figure 10a. At a vibrational energy of 100 Hz, this TENG yielded an open‐circuit voltage of up to 72 V, an *I_SC_* reaching 32 µA, and a peak power density of 0.4 W/m^2^ (Figure 10b,c) [204].

#### 4.2.2 Nylon

In opposition to electron‐withdrawing polymers, a large number of electron‐donating counterparts were investigated, including nylon, which has a molecular structure abundant in amide groups, which exhibit a tendency to lose electrons upon interaction with other materials [159, 205]. Especially, Nylon‐11 can develop highly polar crystalline phases, including the δ′‐phase, which increase surface charge density [206]. These phases can be attained through fabrication techniques such as electrospinning or template‐assisted crystallization, thereby enhancing triboelectric output. About the durability, with its innate flexibility and resistance, nylon could provide constant output for a long time, making it suitable for wearable and elastic TENGs [207, 208]. Notably, nylons’ triboelectric performance could still be enhanced by similar methods, such as electrospinning or incorporating chemicals. A high‐output multilayer‐structured triboelectric nanogenerator (HM‐TENG) was developed to harvest human biomechanical energy by Hao et al. Initially, through simulation and electrospinning process optimization, nylon/L‐cystine composite nanofiber films (NCNF) with different l‐cystine concentrations were fabricated, and SEM images pointed out the existence of l‐cystine on nylon's surface (Figure 10d,e). After choosing the optimal ratio of l‐cystine, which was 8 wt% and the best number of layers (5), the HM‐TENG's performance was evaluated by connecting it to different resistance values, at 5 MΩ, the device obtained the maximum power density of 11.52 W/m^2^ (Figure 10f). When charging different capacitors (100 µF, 220 µF, 330 µF, 470 µF, and 680 µF), the rate was inproportional to the capacitance (Figure 10g) [209].

#### 4.2.3 Cellulose

Unlike the majority of triboelectric materials, which have a strong likelihood of only giving away or only attracting electrons, several materials, on the other hand, can act as both charge acceptors and donors simultaneously, such as cellulose, polyethylene terephthalate (PET), or polyvinyl alcohol (PVA), etc. [210, 211, 212]. With cellulose, due to the natural ether bonds, it already possesses electronegativity [213]. Therefore, to employ the electron‐donating ability, it could be simply attained by linking the appropriate functional groups. Wang et al. chose the cyano group, with this combination, dual‐electric‐polarity augmented cellulose materials are developed by introducing cyanoethyl groups to improve the electrical output of cellulose‐based TENGs. The ether bond's electron‐donating capacity, combined with the cyano group's electron‐withdrawing properties, enhances the contact electrification performance of cyanoethyl‐cellulose (CE‐cellulose) when charged in contact with electropositive nylon or electronegative PTFE. The strong electron‐withdrawing capacity of the cyano group increases the electrical output of the modified cellulose‐polyamide 11‐based TENG as the degree of substitution of the cyanoethyl group rises. The device worked well with the highly electropositive PA11 or the highly electronegative PTFE, and it was attached to the shoes’ insoles to track the walking pattern (Figure 10h) [214]. To achieve the maximum output, the triboelectric charge density (TECD) depends on the degree of substitution (DS), and these indices are proportional to each other regardless of the counter triboelectric materials (Figure 10i). The introduction of cyanoethyl groups can complicate contact electrification. To elucidate their electronic effects on the electrical polarity of polymer films, the *I*sc output of the TENGs containing cyano, ethoxy, and cyanoethyl groups in the main chain of polypropylene (PP) was evaluated. The TENG test results indicated that the addition of these three functional groups significantly altered the electrical output. For C‐PP based TENG, *I*sc increased when nylon‐11 was utilized as the counter triboelectric surface. When the counter material was altered to PTFE, the output decreased, suggesting that the incorporation of cyano‐groups solely enhanced the electronegativity of PP. Second, for E‐PP‐based TENG, the *I*sc decreased when the counter friction electrode was nylon‐11, while it increased when the counter friction layer was PTFE. These results indicated that the introduction of ether bonds improved the electrical positiveness of PP. The CE‐PP‐based TENG exhibited a distinct behavior compared to the previous two instances. Regardless of whether the counter electrode is nylon‐11 or PTFE, the *I*sc of TENG consistently rose, demonstrating a dual‐electric‐polarity augmented property like CE‐cellulose (Figure 10j). About the practical output, Figure 10k illustrates that at 95% RH, the device illuminated 1000 LEDs. On the other hand, due to the extremely high output compared to other sensing effects, the TENGs are being researched extensively in different fields, and some of them were listed in Table 2.

**TABLE 2:** TENGs made from different polymers, with output comparison and various applications.

Tribo‐positive materials	Tribo‐negative materials	*V*_OC_ (V)	Power/power density	Application	Reference
Gelatin	PTFE	55	150	Humidity sensor	[215]
Polyamide	Cellulose	28.5	13.5 W/m^2^	Bioenergy harvester	[216]
Silk	Kapton	400	260 W/m^2^	Wind harvester	[217]
Kapton/alumninum	PEDOT/PPy‐Cell	200	0.6 mW	Water sterilization	[218]
Nylon	PANI/NiCo_2_O_4_	400	2.46 mW/m^2^	Ammonia sensor	[219]
Fur	FEP	435	51.8 mW/m^2^	Hydrogen generator	[220]
Copper	PTFE	100	416 mW/m^2^	Sweat sensor	[221]
Pi‐Cu‐PET	Cu‐PET	4.98	33.16 mW/m^2^	Respiratory sensor	[222]
Ni cloth	PTFE	54.8	196 mW/m^2^	Energy harvester	[223]

### 4.3 Photovoltaic Materials

Besides mechanical energy, light is a ubiquitous energy source in daily life. Consequently, extensive research efforts have been devoted to converting light into electricity through the photovoltaic effect. This process primarily involves two categories of materials: inorganic and organic solar cells (ISCs and OSCs) [7, 224, 225, 226, 227]. Compared to their inorganic counterparts, OSCs have several advantages, such as flexibility, transparency, and light weight [228]. Furthermore, organic semiconductors typically possess lower dielectric constants than inorganic photovoltaic materials. As a result, light absorption in OSCs generates tightly bound Frenkel excitons (hole–electron pairs) rather than free charges, necessitating efficient strategies for exciton dissociation [229]. Structurally, OSCs tend to be less crystalline or even amorphous, in contrast to the highly ordered molecular arrangements found in ISCs. This hierarchical morphology significantly influences charge generation, transport, and recombination dynamics [230]. Despite these differences, OSCs are generally classified into donors and acceptors, where each type will be mixed into the other to create an interpenetrating network with the bulk heterojunction (BHJ) structure for effective charge separation and transport [231, 232]. For wearable applications, the flexible photovoltaic blends also have an appropriate crack‐onset strain (COS) value.

#### 4.3.1 Fullerenes Acceptors (FAs)

**FIGURE 11:**
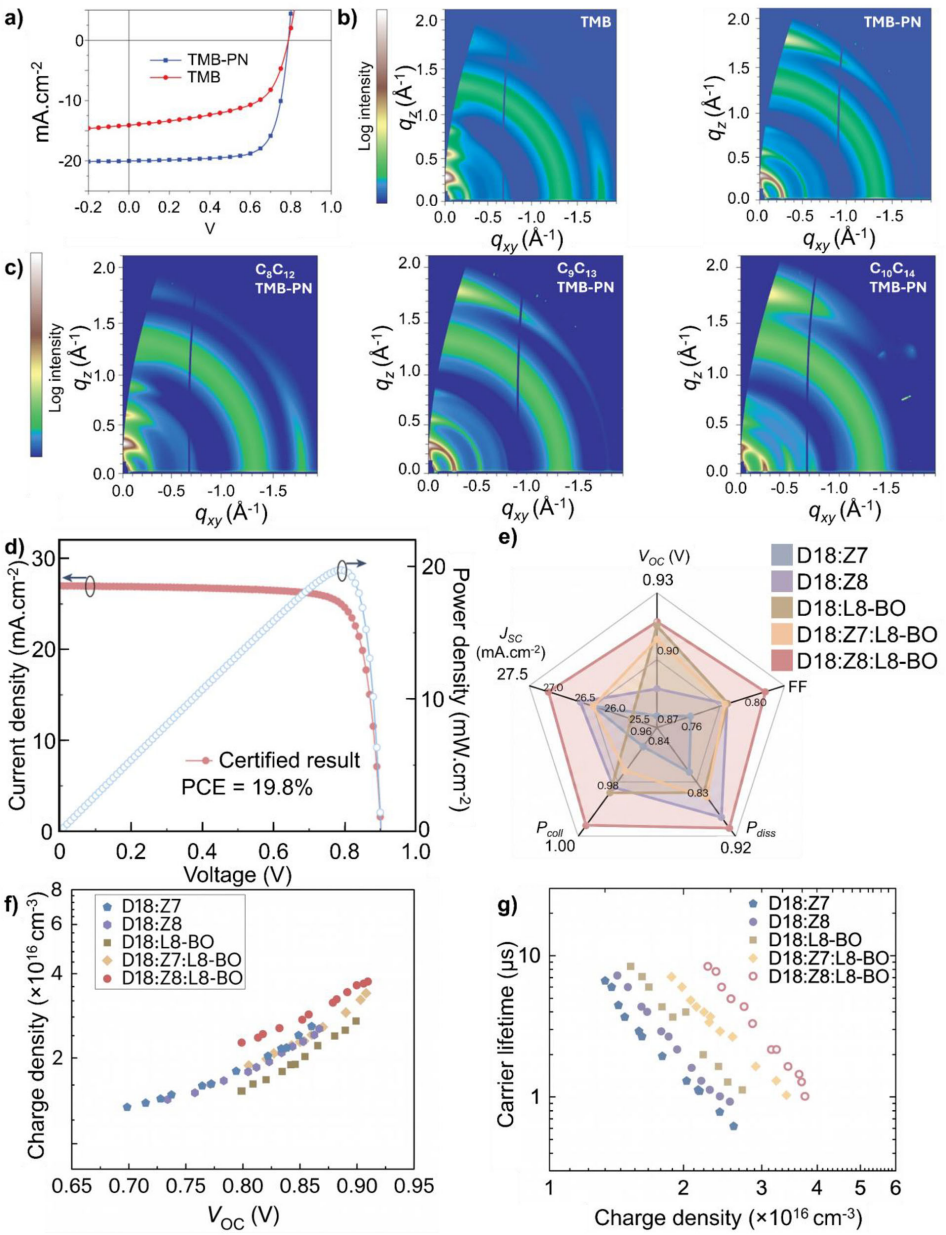
(a) Current density curves of TMB‐PN and TMB; (b) Two‐dimensional GIWAXS images of PffBT4T‐C_9_C_13_:PC_71_BM‐based films with only TMB and with TMB‐PN; (c) From left to right: GIWAXS photos of PffBT4T‐C_8_C_12_:PC_71_BM, PffBT4T‐C_9_C_13_:PC_71_BM and PffBT4T‐C_10_C_14_:PC_71_BM films processed from TMB–PN. Reproduced with permission [234]. Copyright 2016, Springer Nature; (d) The certified 19.8% PCE of the D18:Z8:L8‐BO based on the *J*–*V* and power density curves; (e) Comparison among binary and ternary OSCs in terms of *J_SC_*, *V_OC_*, FF, charge dissociation, and collection probability (*P_diss_* and *P_coll_*); Charge density values against (f) voltage and (g) carrier lifetime of different OSCs. Reproduced with permission [243]. Copyright 2024, Springer Nature. The OSCs use polymer components as active layers or HTLs are having dramatic rise in PCE values, whereas the popular choices are asymmetric NFAs for optimal charge separation efficiency.

The power conversion efficiency (PCE) has significantly improved throughout the development of OSCs. During the early period, fullerene acceptors (Fas) were synthesized and investigated, since they have high electron mobility, favouring charge extraction and lowering recombination loss [233]. And in 2016, Zhao et al. introduced an FA blend, which was poly[(5,6‐difluoro‐2,1,3‐benzothiadiazol‐4,7‐diyl)‐*alt*‐(3,3*^′′′^*‐ di(2‐nonyltridecyl)‐2,2*^′^*;5*^′^*,2*^′′^*;5*^′′^*,2*^′′′^*‐quaterthiophen‐5,5*^′′′^*‐diyl)](PffBT4T‐C_9_C_13_) mixed with PC_71_BM ([6, 6]‐Phenyl‐C71‐butyric acid methyl ester) as the electron donors and acceptors, respectively. During the mixing process, 1,2,4‐trimethylbenzene (TMB) was chosen as the solvent because of its boiling point and fullerenes’ solubility, while 1‐phenylnaphthalene (PN) was selected as the additive due to its low melting temperature but high boiling point. With these chemicals, the integrated current from the external quantum efficiency (EQE) spectrum is 19.9 mA/cm^2^, and PCE reached up to 11.7% under air mass 1.5 global, but without the PN additive, the efficiency dropped to only 6.4% (Figure 11a). To closely evaluate PN's influences on the morphological and photovoltaic properties of PffBT4T‐C_9_C_13_:PC_71_BM, grazing‐incidence wide‐angle X‐ray scattering (GIWAXS) analysis was carried out. Both TMB and TMB–PN exhibited high polymer crystallinity, characterized by pronounced lamellar reflection peaks at (100), (200), (300), and (400), along with clear (010) peaks. Furthermore, both films displayed a bimodal texture that was characterized by the presence of edge‐on and face‐on populations. The (010) π−π stacking and lamellar reflections showed peaks in both in‐plane and out‐of‐plane directions. The GIWAXS results indicated that the isotropic peak at q ≈ 1.33 Å is associated with the aggregates of PC_71_BM. The coherence length of the PC_71_BM peak in the blend film processed from TMB–PN is estimated to be approximately 6.2 nm, which is longer than the 4.7 nm observed in the film processed from only TMB (Figure 11b). The same analytical method was used to reveal the effect of the alkyl chain length on the photovoltaic performance of PffBT4T‐C_8_C_12_, PffBT4T‐C_9_C_13_, or PffBT4T‐C_10_C_14_. As shown in Figure 11c, for the C_8_C_12_ variant, the (010) π−π stacking peak was primarily situated in the in‐plane direction, but for the C_9_C_13_ and C_10_C_14_ samples, the same stacking peak was located in the out‐of‐plane direction, meaning the face‐on orientation is more pronounced with longer alkyl chains. However, as the alkyl chain length increased, the domain purity decreased. Therefore, PffBT4T‐C_9_C_13_ was the optimal selection. This study presented a straightforward and effective method for achieving highly efficient and environmentally friendly organic solar cells (OSCs) from Fas [234].

#### 4.3.2 Non‐Fullerene Acceptors (NFAs)

Besides its advantages, FAs have some main drawbacks, which are poor absorption in the UV‐Vis region and the requirement of energy level adjustment [226]. However, in 2015, NFAs with fused‐ring electron acceptors (FREA), including the star molecule ITIC, were introduced and have become the potential materials for photovoltaics [235, 236, 237]. In this groundbreaking invention, the fused core unit indacenodithieno[3,2‐b]thiophene (IDTT) contained four out‐of‐plane 4‐hexylphenyl side chains, which enhanced the solubility of the acceptor and inhibited excessive aggregation, resulting in improved morphology. The 1,1‐dicyanomethylene‐3‐indanone (IC) end units exhibited efficient π–π stacking, thus benefiting electron transport. The pronounced electron intramolecular push‐pull effect results in a narrow energy gap of ITIC, facilitating robust light absorption in the 500–800 nm range. Consequently, cells utilising ITIC and the polymer donor (PTB7‐Th) achieved power conversion efficiencies of approximately 6.8%. Overall, this compound belongs to the ADA (acceptor‐donor‐acceptor) type. This study laid the foundation for the development of NFAs and made them more popular than the FAs. The inclusion of twisted or out‐of‐plane side chains improved the BHJ morphology. Following this, in 2019, Y6, a new NFA was introduced, it is one of the Y‐series acceptors that possess the ADA'DA structure [238]. In comparison to ADA, the ADA'DA‐type materials have higher interaction among D/A active layers and facilitate the interaction between neighbouring acceptor molecules or conjugated polymer donor backbones. The central fused‐ring core, featuring gradient‐shaped nuclei that are formed by a central A′ unit and adjacent D units on either side, enhances electron transport performance, leading to more effective electron mobility than ADA‐type NFAs. The synergistic interaction of electron‐deficient core A′ units and specific molecular geometry enables ADA'DA‐type NFA molecules to attain both low voltage loss and high current generation. Because Y6 belongs to the ADA'DA category, it resembles the overall chemical structure; in particular, the core has an electron‐deficient benzothiadiazole fragment. The molecule has four side chains, with two linked to the internal pyrrole rings and two connected to the external thiophene rings. The steric repulsion between the internal alkyl chains results in a degree of twist in the molecular plane of Y6, extending the internal alkyl chains out of the plane. Meanwhile, the two external alkyl chains hinder the rotational angles of the DFIC end units, leading to increased conformational rigidity and uniformity of Y6. Acknowledging these characteristics, Liu et al. mixed it with D18 (a polymer donor with a wide bandgap), and the mixed film reached a maximum PCE of 18.22% [239]. Following this, some research also achieved more than 19% PCEs by employing Y6 [240, 241, 242]. Another state‐of‐the‐art NFA is Z8, it is asymmetric and includes tethered phenyl functional groups to form an alloy acceptor. On the one hand, this asymmetric configuration delocalized excitons, thus, minimizing the non‐radiative energy loss and charge recombination. On the other hand, the phenyl‐substituted alkyl side chain influences intermolecular interactions, enhancing film nanomorphology, facilitating efficient exciton dissociation, and decreasing charge recombination. This is the study of Jiang et al., their D18:Z8:L8‐BO ternary blend provided a PCE output of 20.2% (certified 19.2%), because L8‐BO possessed a higher Lowest Unoccupied Molecular Orbital (LUMO); therefore, it was juxtaposed with the Z‐acceptors to obtain higher performance (Figure 11d). This ternary blend created 0.92 V, 27.2 mA/cm^2^, and 80.8%, which outperformed other binary and ternary OSCs in every aspect (Figure 11e) [243]. It also had the highest charge carrier density versus voltage and carrier lifetime, meaning it is the most effective charge‐generation blend compared to the others (Figure 11f,g). In conclusion, the asymmetric Z8 could alleviate charge recombination and simultaneously improve charge transport and extraction. Furthermore, even though most NFAs are small molecules, they are still suitable for flexible photovoltaics because of their stability and processability. There are some strategies to further improve their PCEs, and Table 3 is made to compare some common photovoltaic agents. From the table, many photovoltaic blends used indium tin oxide/Poly(3,4‐ethylenedioxythiophene) polystyrene sulfonate (ITO/PEDOT:PSS) as the transparent conductive layers, whereas PEDOT:PSS is a conductive polymer composite and the hole transporting layer (HTL). It can prevent electrons, and transport holes, thus enhancing the electrodes’ work function and creating effective Ohmic contact between ITO and the active layer. Moreover, PEDOT:PSS can smoothen the ITO surface to decrease the leakage current and modify the active layer's morphology [226, 244]. Generally, the polymers could either be the main active or the HTL layers in OSC systems, demonstrating their versatility in the photovoltaic field.

**TABLE 3:** Performance comparison table of different photovoltaic agents.

Photovoltaic agents (donor:acceptor)	Transparent conductive layers	*V*_OC_ (V)	*J*_SC_ (mA/cm^2^)	FF (%)	PCE (%)	Reference
P3HT:PC_61_BP^F^110	ITO/PEDOT:PSS	0.60	10.26	66	4.08	[245]
P3HT:ICBA	ITO/PEDOT:PSS	0.84	7.20	71	4.50	[246]
P3HT:OQBMF	ITO/PEDOT:PSS	0.95	9.67	70	6.43	[247]
P3HT:IC_70_BA	ITO/PEDOT:PSS	0.87	11.35	75	7.40	[248]
PTB7:PC_61_BM	ITO/PEDOT:PSS	0.76	13.70	57	6.00	[249]
PDTBDT‐BZF:PC_71_BM	ITO/PEDOT:PSS	0.88	12.65	72.6	8.08	[250]
PBDB‐T:HF‐TCIC	ITO/PEDOT:PSS	0.76	20.04	65	9.40	[251]
P3HT:PCE10	ITO/ZnO	1.03	17.30	61	11.00	[252]
PBDB‐TFS1:IT‐4F	ITO/PEDOT:PSS	0.90	20.30	71	13.00	[253]
D18: BTZT‐4Cl/BTZT‐2Cl	ITO/PEDOT:PSS	0.96	20.98	76.2	15.41	[254]
PM6:Y6 with DBCl	ITO/PEDOT:PSS	0.85	25.80	78.5	17.20	[255]
PM6:L8‐BO	ITO/PEDOT:PSS	0.90	26.70	81.9	19.30	[256]
PM6/Y6 with DCBB	ITO/PEDOT:PSS	0.83	28.15	77.49	18.19	[257]
PM6: o‐BTP‐eC9/BTP‐eC9	ITO/PEDOT:PSS	0.86	28.75	80.41	19.50	[258]
D18:L8‐BO/P‐2BTh‐F	ITO/PEDOT:PSS	0.92	26.47	79.09	19.45	[259]

### 4.4 Pyroelectric Polymers

As discussed in the previous section, thermal energy can also be converted into electricity through pyroelectric mechanisms [260, 261]. Although having low pyroelectric efficiency, the pyroelectric polymers have much better flexibility and chemical resistance [262]. And PVDF and its derivations are the most attractive pyroelectric polymers [263]. Therefore, to improve the pyroelectric output of these polymers, additional fillers were added without sacrificing the flexibility. For example, Zou et al. proved the incorporation of barium strontium titanate nanofibers (BST NFs) and (PCBM) into poly(vinylidene fluoride‐*ter*‐trifluoroethylene‐*ter*‐chlorofluoroethylene) (P(VDF‐TrFE‐CFE)) could boost the electrocaloric effect (ECE) of this co‐polymer and reduce energy loss due to Joule heating at elevated fields and temperatures simultaneously. In this ternary composite, the nanofibers played the role of regulating the P(VDF‐TrFE‐CFE)’s crystallization and polarization via the ceramic–polymer interfacial coupling effect, producing higher ECE. Meanwhile, PCBM modified the energy band to increase the electrical resistance value. The composite demonstrated a significant electrocaloric effect, characterized by a temperature change (*ΔT*) of 18°C and an isothermal entropy change (*ΔS*) of 0.17 J/cm^3^.C, across a broad temperature range of 0–80°C. Additionally, it exhibited exceptional cyclability due to the durable performance over 100 000 cycles under an applied field of 150 MV/m at 80°C [264]. Li et al. recently introduced the liquid crystalline elastomer‐lead zirconate titanate composite (LCE‐PZT) as an innovative flexible pyroelectric material. According to the authors, LCE possesses thermal stress, but this elastomer could not solely generate piezoelectric signals. Instead, it supported PZT NPs to produce additional pyroelectricity. Under temperature fluctuations, the thermal stress from LCE was transferred to the PZT fillers. Regarding the PZT adoption concentration, the titanate particles were added in a high amount (10‐60 wt %) by cross‐linking with 3‐(trimethoxysilyl)propyl methacrylate (TMSPMA). The higher loading boosted the pyroelectric performance due to greater density. Moreover, the covalent bonding between PZT NPs and the LCE matrix enhanced interfacial contact, facilitating efficient thermal stress transfer between the two components [265]. As shown in Table 4, PVDF and its co‐polymers are still the common choices for pyroelectric flexible materials, along with innovative candidates like polysiloxanes.

**TABLE 4:** Fabrication techniques, pyroelectric coefficients, and applications of different polymers/composites.

Polymers/composite	Fabrication technique	*P* (µC/m^2^ K)	Figure of merit	Application	Reference
PVDF	Casting	26.4	—	Hybrid nanogenerator	[266]
LiNbO_3_/PVDF	Solvent casting	127	*F_i_* = 104.96×10^−3^ µCm/J	Flexible sensor	[267]
PVDF‐TrFE	Casting	43	*F_i_* = 20.8×10^−6^ µCm/J	Flexible sensor	[268]
LiTaO_3_/PVDF‐TrFE	Solvent casting	327	*F_i_* = 286×10^−3^ µCm/J	Infrared sensor	[269]
MXene/PVDF	Electrospinning	0.97	*F_i_* = 5.25×10^−7^ µCm/J	Infrared detector	[270]
Polysiloxanes‐TiO_2_	Three‐roll‐milled	3.4	—	Pyroelectric sensor	[271]
KNbO_3_/PDMS	Spin‐coating	8	—	Pyro nanogenerator	[272]

### 4.5 Piezo‐Coupling Polymeric Materials

**FIGURE 12:**
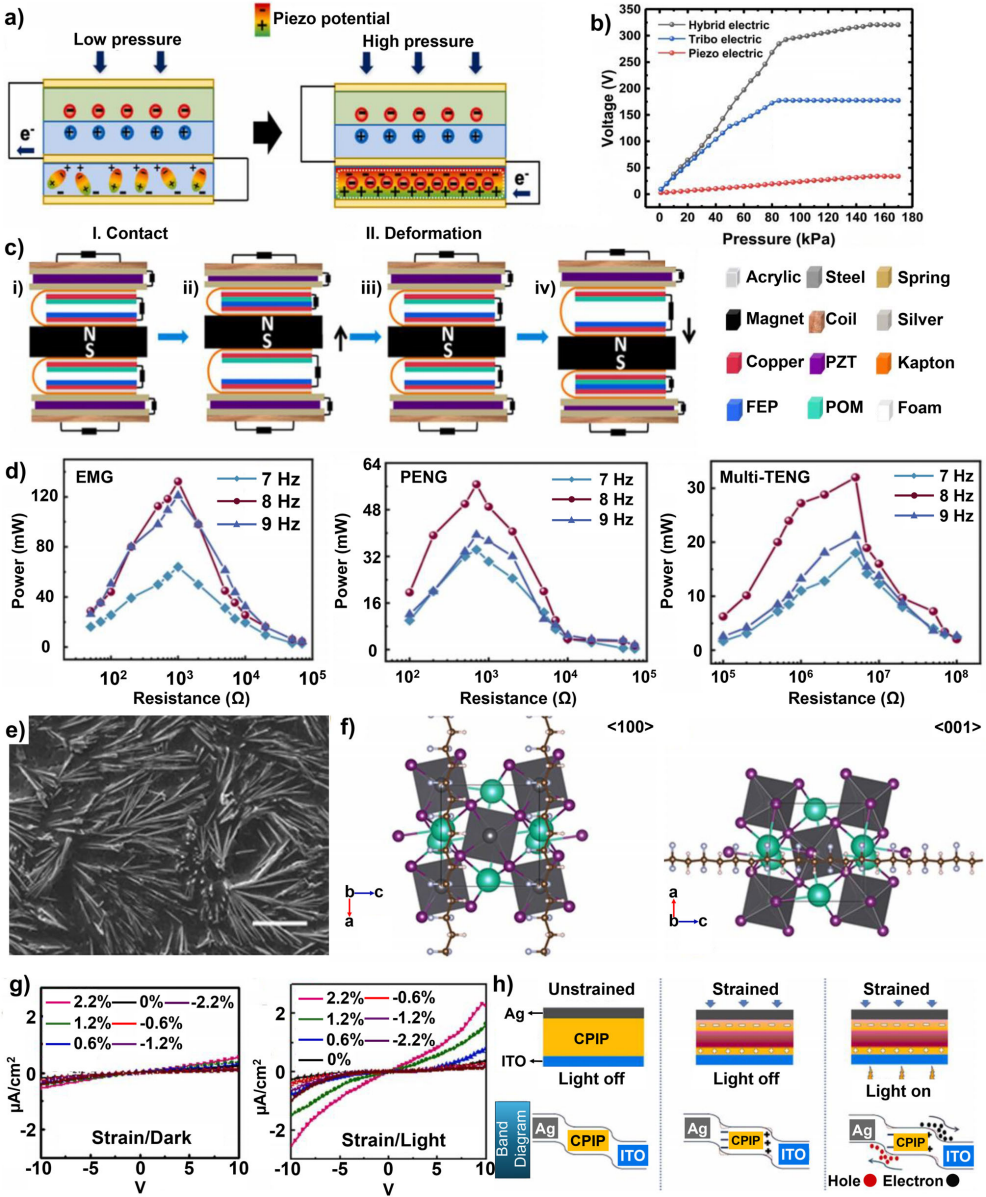
(a) Triboelectric and piezoelectric potential generation principle of the hybrid sensor; (b) Voltage output comparison among the piezo/tribo/hybridelectric pressure sensors at different pressure values. Reproduced with permission [133]. Copyright 2025, Elsevier; (c) Schematic illustration of the HTG's working process; (d) The power productivity from different resistance and frequency of the EMG, PENG, and Multi‐TENG. Reproduced with permission [57]. Copyright 2024, Elsevier; (e) SEM photo of the CPIP film; (f) The DFT calculation of the stretching PVDF along <100> direction with PbI_2_ surface and <001> direction with CsI surface; (g) The *I–V* curves of the CPIP‐based device under various compressive and tensile strains when in the dark and when irradiated by light; (h) Schematic structure and energy band diagrams of the piezo‐phototronic CPIP in different states: unstrained‐light off, strained‐light off, and strained‐light on. Reproduced with permission [275]. Copyright 2022, Elsevier. Incorporating multiple sensing effects, such as piezoelectric, phototronic, electromagnetic, etc., into a single sensing platform could significantly improve the output, laying the groundwork for smart wearable electronics.

Apart from relying solely on one physical sensing effect, considerable research efforts have been directed toward integrating multiple effects into a single sensing platform to enhance overall performance. For instance, both piezoelectric and triboelectric effects are capable of converting mechanical energy into electrical energy, making them suitable for hybrid integration. Recent advancements in these hybrid piezo‐coupling systems have garnered interest for their potential to power various wearable electronic devices, owing to their capability to produce elevated output current and voltage. Suitable structural designs and polarization direction modulations are critical factors for achieving combined piezoelectric and triboelectric effects. The piezoelectric and triboelectric effects can be effectively combined due to their matching impedance, comparable structural characteristics, and their shared ability to convert mechanical energy into electrical energy [273]. Under external force, the piezoelectric materials produce a potential difference across the surface; concurrently, the triboelectric pair creates a charge potential difference on the material surfaces during contact and separation cycles. For instance, Choi et al. reported this method for the rapid‐response hybrid piezo‐triboelectric pressure sensor. Overall, the device contained two triboelectric halves, with the positive part made of nylon‐6,6 and an electrode, while the negative counterpart had four layers in the following order: PMMX/Electrode/PMMX/Electrode (PMMX: PVDF/MoS_2_/MXene), whereas the top PMMX contributed in receiving triboelectric charges from nylon‐6,6, and the bottom one produced piezoelectric potentials (Figure 12a). Besides the structure, it is important to note that the material selection also plays a crucial role in the hybrid TENG‐PENG. In the same article, the PMMX included PVDF, which owned both mechanisms due to its crystal structure and electron affinity, this multi‐functional polymer was linked to Mxene by hydrogen bonds. These linkages improved the PVDF's β‐phase composition for higher piezoelectricity. On the other hand, MoS_2_ monosheets with the 1T‐phase configuration alongside ferroelectric PVDF, which dramatically improved charge transfer within PVDF, consequently boosting the piezoelectric properties of this polymer. The MoS_2_ nanosheets demonstrated interaction with the ─CH_2_ dipole of PVDF, which polarized the polymer chain and enhanced the β‐phase, contributing to the piezoelectric output. The correlation between the applied pressure and the output voltage, the piezoelectric voltage exhibited a linear rise in response to pressure, saturating at around 150 kPa. This linear response aligned with the core principles of the piezoelectric effect, indicating that charge generation is directly proportional to the applied stress, and saturates upon the material's elastic limit. In contrast, the triboelectric voltage exhibited a responsive trend. The voltage increment was notable from 0 to 50 kPa, then reached saturation around 85 kPa. This phenomenon can be explained by the mechanism of triboelectric nanogenerators, where the charge generation is initially very responsive to variations in the contact area, but levels off once complete contact is established. Meanwhile, under the same experimental condition, the hybrid electric pressure sensor inherited benefits from both mechanisms. The voltage gradient showed a reduction from 85 kPa, which corresponded with the saturation point of the triboelectric component, indicating a trend towards saturation at 150 kPa, similar to the piezoelectric response. This composite behavior suggested that the triboelectric and piezoelectric effects were the primary and supporting power sources (Figure 12b) [133].

Beyond coupling effects that share similar energy conversion mechanisms, it is also feasible to integrate multiple phenomena originating from distinct energy sources. For example, the dual piezo‐triboelectric effect can be coupled with electromagnetism to convert energy from mechanical and magnetic forces. Taking advantage of this effect, Wang et al. presented a compact trihybrid sensor, called a hybrid piezo‐tribo‐electromagnetic generator (HTG). Its working principle was illustrated in Figure 12c, where the magnet was the middle layer (Figure 12c(i)) in the resting state. Upon vibration, the magnet ascended towards the upper copper coil, resulting in a magnetic flux increase within the upper coil. Consequently, the upper Multi‐TENG's layers contacted, and the pressure of the upper PENG was augmented (Figure 12c(ii)). Subsequently, influenced by gravitational forces, the magnet reverted to its central position (Figure 12c(iii)). As the magnet approached the lower coil, the magnetic flux through it changed accordingly. At the same time, the bottom Multi‐TENG entered a contact state, compressing the bottom PENG (Figure 12c(iv)). Ultimately, when the vibration returned, the entire device returned to its initial position. With this working procedure, the HTG could persistently transform vibration into electricity. Regarding performance, given the identical working vibrational frequency (8 Hz), the electromagnetic generator (EMG), Multi‐TENG, and PENG generated 126 mW, 32.6 mW, and 57.4 mW, respectively (Figure 12d). With this record, self‐powered wireless motion monitoring systems, utilising HTG, were built for real‐time tracking of various motion parameters, including motion speed, frequency, and displacement, through the collection of vibration energy [57].

The integration of piezoelectric, photovoltaic, and semiconducting properties has led to the development of the piezophototronic effect, which enhances charge separation and optimizes energy conversion efficiency in optoelectronic devices [97, 274]. Piezo‐phototronic polymers represent an advanced class of materials that combine the mechanical flexibility and ease of fabrication inherent to optoelectronic polymers with the multifunctional capabilities of piezo‐phototronic effects. Recently, Maity et al. revealed a piezo‐phototronic CsPbI_3_‐PVDF (CPIP) composite, with CsPbI_3_ as the main phototronic agent with high sensitivity, whilst PVDF provided the piezoelectric signal and enhanced the electroactive phase, and this polymer stabilized and protected CsPbI_3_ from the environment. From the SEM image, it could be observed that the CsPBI_3_ nanowires (NWs) were distributed and embedded uniformly across the transparent PVDF matrix (Figure 12e). Following morphological analysis, the authors carried out density functional theory (DFT) measurements to explore the bonds within CPIP. According to the study, the stretching direction of PVDF tends to favour the bandgap reduction along the <100> direction on the PbI_2_‐terminated surface and along the <001> direction on the CsI‐terminated surface (Figure 12f). Consequently, these elongated directions decreased the band gap considerably and maintained a stable state for the CPIP composite. After validating the composite's interfacial, the CPIP compound's piezo‐phototronic performance was evaluated. Under the same strain and voltage parameters, the light‐irradiated sample exhibited higher current density values, and the output gap correlated to the tensile strain magnitude. However, with the compressed strain, the divergence was minor (Figure 12g). Additionally, Figure 12h elucidated why the piezo‐phototronic effect was better than piezoelectric only [275]. Some other research about polymeric piezo‐coupling devices are listed in Table 5 to provide a broad overview.

**TABLE 5:** Different piezo‐coupling polymeric composites with their key output and potential applications.

Polymers/composite	Piezo‐coupling effects	Key performance	Application	Reference
Cs_2_SnI_6_‐PVDF	Piezo‐phototronic	*V_OC_* ∼ 15 mV; *I_SC_* ∼100 nA	Strain modulated optical sensor	[276]
CsPbBr_3_‐ZnO@PVDF‐HFP	Piezo‐phototronic	R_r_ ∼ 5.7 × 10^−7^ A/W; R_s_ ∼ 3.2 × 10^−6^ A/W	Piezo photodetector	[277]
PEAn‐doped P(VDF‐TrFE)	Piezo‐pyroelectric	*V_piezo‐pyro_* = 188.46 V; *V_piezo_* = 57.83 V	Self‐heating therapeutic pad	[278]
PAN/Zn(Ac)_2_	Piezo‐pyroelectric	*V_25_* ∼14.13V; *V_450_* ∼14.86V	High‐temp sensor	[279]
γ‐GC/chitosan	Piezo‐triboelectric	*V_OC_* ∼ 79 V; *I_SC_* ∼ 64 µA; *P* ∼ 9.59 µW	Hybrid nanogenerator	[280]
C_60_‐P(VDF‐TrFE)	Piezo‐pyro‐phototronic	*p* = 20 µC/m^2^·K; *d_33_* = 25 pC/N	Multifunctional sensor	[281]

## 5 Applications

### 5.1 Multifunctional Wearable Sensors

**FIGURE 13:**
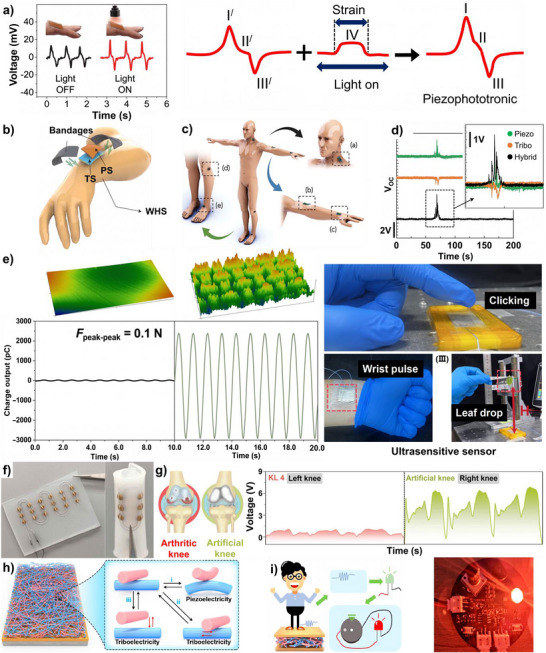
(a) Output signal from the piezo‐phototronic device attached to a finger during bending and releasing at 30° with and without light illumination. Reproduced with permission [283]. Copyright 2025, American Chemical Society; (b) Device structure of the WHS; (c) WHS positions attached to the body for human motion detection; (d) Signal analysis of the WHS's piezoelectric, triboelectric, and hybrid electric. Reproduced with permission [284]. Copyright 2021, John Wiley and Sons; (e) Surface morphology, charge output of the flat and microwrinkles PVDF‐TrFE and the experimental setup of the microwrinkles PVDF‐TrFE film for finger clicking, wrist pulse, and leaf drop detection. Reproduced with Creative Commons CC BY‐NC license [289]. Copyright 2025, American Association for the Advancement of Science; (f) Digital photos of the hierarchical ceramic composite; (g) Voltage response of the device with a knee suffering from KL level 4 (left‐red) and joint replacement knee (right‐green) [290]. Reproduced with Creative Commons CC BY license. Copyright 2024, John Wiley and Sons; (h) Schematic illustration of electrospinning the piezo/triboelectric fibers with coupling mechanism; (i) Illustration and digital images of the hybrid sensor work as a component for the alarm circuit. Reproduced with permission [291]. Copyright 2023, American Chemical Society. The wearable sensors employ various piezo‐coupling effects and device configurations to boost the sensitivity whilst still maintaining the ergonomic for physiological and motion tracking.

By leveraging the unique ability of piezoelectric materials to convert mechanical stimuli into electrical signals and enormous potential in terms of flexibility and performance, the piezo‐coupling effect has attracted substantial research interest, particularly in the field of wearable sensors. Most wearable piezo‐pyroelectric sensors are developed to track physiological signals such as human breath, heartbeat, etc. With the piezo‐phototronic coupling effect, strain‐induced piezoelectric charges could stimulate charge carrier generation, separation, and transportation. For wearable applications, adding semiconductor nanoparticles (phototronic agents) into the flexible piezoelectric polymers is still the widely known method, CeO_2_@PDA or WS_2_/PVDF composites were synthesized and proved to have piezo‐phototronic effect [15, 282]. The flexible polymers allow wearability and repetitive compress‐strain cycles to boost the optoelectronic output. Consequently, piezo‐phototronic devices are capable of detecting motion or environmental light. Recently, Arora et al. introduced an all‐organic, flexible, single‐crystal‐based sensor that could provide strain‐dependent photoresponse. Under the same bending angles, the sample provided higher output under light radiation. However, the photonic peaks were only noticeable when the device was being strained (Figure 13a) [283]. On the other hand, triboelectric is often coupled due to its higher electrical output, broader sensitivity, and wide material selection. Many devices working on the piezo‐triboelectric are self‐powered, and some could power small electronics. Due to the superior output, researchers could focus on other characteristics to make the devices more suitable for practical applications. Moreover, with their renowned robustness, piezo‐triboelectric sensors are usually attached to external body parts to analyze footsteps, limb bending intensity, or even breath patterns. For example, Mariello et al. focus on maximising the device's comfort by developing an ultra‐thin piezo/triboelectric sensor based on aluminium nitride (AlN) and a parylene‐encapsulated polymeric blend. Figure 13b explains the working principle of the wearable hybrid sensor (WHS). The device can be attached to the skin using medical bandages, allowing it to deform according to human movement without limiting skin transpiration (Figure 13c). With these advantages, the WHS was able to be mounted on different body parts. The independent and synergistic effects of the sensor were recorded in Figure 13d [284].

Structural optimization represents an effective strategy for enhancing stimuli‐responsiveness while preserving the simplicity of device architecture. Research efforts have been made to modify the microstructure of the functional sensing layer. There are several main structures, which are micro‐patterned, hierarchical, and porous [285]. Micro‐patterning the sensing layers enhances local stress concentration and increases deformation under small strains, which results in improved piezo‐performance and dipole density, respectively [286]. Many articles reported different micro‐geometric structures, and every kind has its advantages. For example, micro pyramids provide substantially improved sensitivity, while Kirigami designs offer better stretchability [287, 288]. Recently, Li et al. introduced an ultrasensitive piezoelectric sensor with microwrinkle patterns. After modifying the flat PVDF‐TrFE film into a rough surface with a lot of microwrinkles, the device had a significantly higher charge output and an outstanding *d_33_* coefficient of 2.1 × 10^4^ pC/N. By exploiting the advantages of the microstructure, the sensor could detect finger clicks, pulse rates, or leaf drops precisely (Figure 13e) [289]. Interlocking various microgeometric structures results in a device with a combined hierarchical architecture. This combination significantly enhances the contact area between the micro patterns, generating numerous stress‐focused regions for enhanced responsiveness and durability. For instance, Xu et al. reported a piezoelectric sensor from hierarchical droplet‐shaped BZCT ceramics (Figure 13f). The biomimetic device had a soft‐rigid hybrid configuration and spectacular piezo‐responsivity when it could detect different simultaneous deformations. With these advantages, the hierarchical sensor was attached to the knee for knee osteoarthritis diagnosis. The non‐operated left knee exhibited a Kellgren–Lawrence grade 4, with an average voltage measurement of 1 V. The surgically remediated right knee with joint replacement exhibited a voltage value comparable to that of the control group (7 V) (Figure 13g). This study indicated that stretchable ceramic sensors possess significant potential for use in clinical post‐surgery [290]. For maximum comfort, the porous structure is chosen, as it has a larger specific surface area and more air passing through the layer compared to the planar structure. Therefore, a mechanical stress will cause more deformation and localized stress concentration on the porous sensing layer, facilitating better piezo‐coupling output. Textile with fibrous structure is one of the most common types of porous material. For example, Huang et al. applied co‐electrospinning techniques to fabricate two fibrous composites at once; the interwoven fibers had a dual‐coupling effect for sensitive motion recognition and some electronic powering (Figure 13h,i) [291]. The authors successfully interlocked two porous microstructures to fabricate a multilayer hierarchical sensing layer. While the vast majority of polymer‐based piezo‐coupling sensors are partial self‐powered, there are several reports about fully closed‐loop sensing platforms. To get rid of external power sources, these devices integrate power management and storage modules. They mostly operate under a piezo‐triboelectric mechanism, as triboelectricity could provide the highest electrical output compared to other sensing effects. Until now, due to relatively complicated structures, these fully autonomous sensors are mostly applied in traffic detection. Table 6 summarizes some notable piezo‐coupling sensors (both partially and fully closed‐loop subcategories) in terms of working principles, output, and applications. It could be seen that many partial self‐powered devices have high sensitivity and are more suitable for wearable sensing [76, 292, 293, 294, 295, 296, 297, 298, 299, 300, 301, 302, 303, 304]. In contrast, although the fully closed‐loop system could provide extremely high‐power efficiency, its rigid structures are limiting them from wearable applications [56, 305, 306].

**TABLE 6:** Wearable energy‐conversion sensors and their applications.

Wearable devices	Working principle	Key performance	Application	Reference
Self‐healable fibrous TENG	Triboelectric	*V_OC_* = 300 V; *I_SC_* = 30 µA *P_d_* = 4.2 W/m^2^	Wearable energy harvester	[76]
Wireless physio health monitoring system	Triboelectric	*V_OC_* = 266 V; *I_SC_* = 0.49 µA *P_d_* = 0.2 W/m^2^	Wireless sport fitness analytic	[292]
ZnO‐based single electrode TENG	Triboelectric	Sensitivity ∼ 0.2 V/µL Detection limit ∼ 4.8 µL	Sweat sensing	[293]
3D piezoelectric nanoyarn fabric sensor	Piezoelectric	*V_OC_* = 8.45 V; *I_SC_* = 1.08 µA	Patient supervision and wireless alarm	[294]
Heterogeneously hierarchical piezoelectric sensor	Piezoelectric	*d_33_* = 41.67 pC/N Piezo sensitivity = 39.3 mV/kPa Response time = 30.1 ms	Blood pressure and cardiovascular recording	[295]
Ca‐BZT/PDMS hybrid nanogenerator	Triboelectric + Piezoelectric	*V_OC_* = 550 V; *I_SC_* = 34 µA *P_d_* = 23.6 W/m^2^	Biomedical energy harvester	[296]
P‐Bi_2_Te_3_/P(VDF–TrFE) pyroelectric sensor	Pyroelectric	*V_OC_* = 550 V Pyro sensitivity = 0.59 V/°K	Wearable pyro‐detector	[297]
Flexible hybrid nanogenerator	Triboelectric + Piezoelectric + Photovoltaic	*V_OC_* = 14 V; *I_SC_* = 0.119 µA *P_d_* = 0.088 W/m^2^	Self‐powered weather and healthcare sensor	[298]
P(VDF‐TrFE)‐PEDOT:PSS hybrid sensor	Piezoelectric + Pyroelectric	*d_piezoelectric_* = −86 pC/N *d_pyroelectric_* = 95 µC/m^2^.K	Physical motion and physiological signals detection	[299]
Self‐powered flexible antibacterial tactile sensor	Triboelectric + Piezoelectric + Pyroelectric	Piezo sensitivity = 0.092V/kPa Pyro sensitivity = 0.11 V/°C	Self‐powered, antibacterial, wearable sensor	[300]
Thermoplastic:NF‐SMA photovoltaic device	Photovoltaic	PCE ∼ 19%; FF = 61.3% COS = 13.0 ± 1.1% Modulus: 0.28 ± 0.09 GPa	Stretchable and wearable OSC	[301]
All‐polymer photovoltaic blend	Photovoltaic	PCE = 18%; FF = 80.7% COS = 25.5 + 2.3% Modulus = 3.90 Gpa	Stretchable and wearable OSC	[302]
Fiber‐shaped UV photodetector	Piezo‐phototronic	Responsivity = 156 µA/W Detectivity = 0.74×10^9^ Jones *P_d,_* = 0.0325 W/cm^2^	Wearable, self‐powered, fast UV sensor	[303]
Piezo‐optoelectronic energy harvester	Piezo‐phototronic	*V_OC_* = 51.7 V; *I_SC_* = 4.6 µA On photoresponse: 3.22 s; Off photoresponse: 5.48 s	Optical signal‐modulated piezoresponsive wearable sensor	[304]
Self‐sustained autonomous wireless sensor	Triboelectric + Piezoelctric	RMS power = 6.5 mW in 1.0 g acceleratation at 25 Hz; Sensitivity of 15V/g	Self‐sustainable monitoring systems	[306]
Self‐powered RF transmission system	Triboelectric + Piezoelectric	Conversion efficiency ∼ 66.21% Working distance: 28‐100 m	Disaster rescue/relief	[305]
Hybridized nanogenerator	Triboelectric + Piezoelectric + Electromagnetic	*P_TENG_* = 78.4 µW; *P_EMG_* = 38.4 mW; *P_PENG_* = 122 mW	Vibration energy harvesting, IoTs	[56]

### 5.2 Multifunctional Implantable Sensors

**FIGURE 14:**
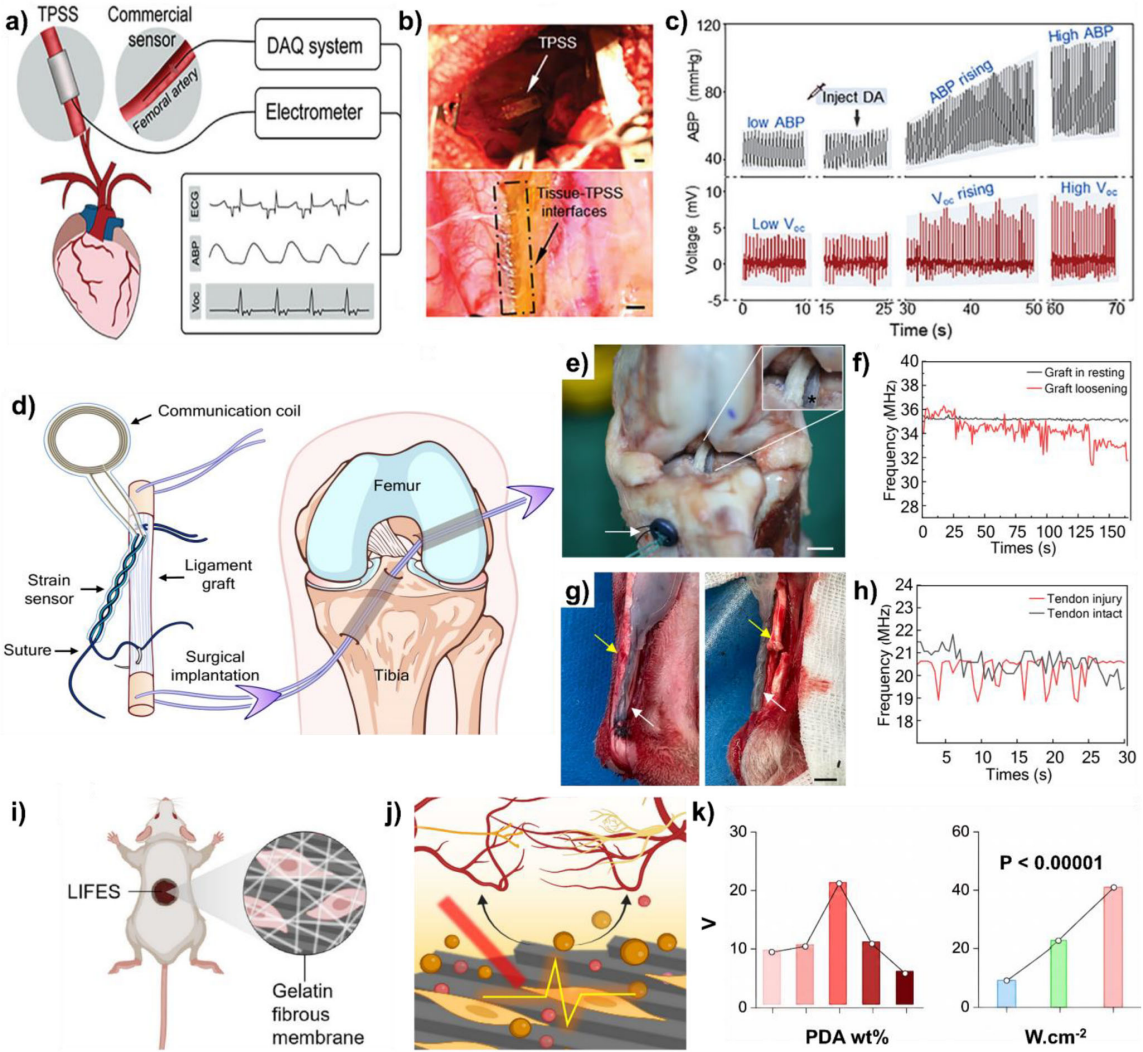
(a) Image of the TPSS closely attached to the surface of the right‐side internal carotid artery. Scale bar: 10 mm; (b) Signal comparisons between the ABP (mmHg) from the commercial sensor and voltage (mV) from the TPSS; (c) The ABP signals’ change was realized by the TPSS. Reproduced with permission [311]. Copyright 2023, John Wiley and Sons; (d) Schematic illustration of how the suture‐based sensor was implanted inside body part (inset image: circuit diagram of the implantation system and the external device); (e) A photograph of the in‐situ graft‐sensor complex following ACL restoration via tibia tunnel, the magnified image revealing the sensor and graft. Scale bar: 10 mm; (f) Frequency responses of the resting and loosen grafts during the Lachman test for the surgical‐induced loosening experiments; (g) The implantation of the suture sensors inside an undamaged and a half‐severed tendon. Scale bar: 5 mm; (h) During ankle joint flexion, the surgically produced Achilles tendon injury model showed a more distorted resonance frequency response than the intact sample, suggesting damage to the tendon's integrity and excessive strain on the surrounding tissues. Reproduced with Creative Commons CC BY‐NC license [312]. Copyright 2025, American Association for the Advancement of Science; (i) Schematic illustration of how LIFES was implanted on a rat's diabetic wound; (j) The working principle of LIFES for the remodel of neurovascular, the device made from PDA/PVDF‐TrFE composite with unique topographical/piezoelectric/photopyroelectric signals, and a cell layer with physiology‐mimicking multi‐target; (k) The relationship between PDA contents and 808 nm NIR radiation. Reproduced with permission [313]. Copyright 2025, Elsevier. The biocompatible piezo‐coupling effect polymers could generate electrical pulses from biomechanical motion, enabling vital signs or joint strain detection, and facilitate neurovascular recovery.

In addition to wearable sensors, piezo‐coupling effects are increasingly being applied to implantable sensors. Within the human body, three primary forms of bioenergy exist: biochemical, biothermal, and biomechanical. Among these, biomechanical energy is the most prevalent, as it arises from continuous micro‐motions generated by internal organs [144, 307, 308, 309]. Consequently, piezoelectric and triboelectric effects are the main approaches for harvesting biomechanical energy. For implantable applications, materials must exhibit biocompatibility to prevent toxicity and adverse reactions within biological tissues. Moreover, the devices must possess high sensitivity to detect subtle physiological movements and be sufficiently miniaturized to enable safe and effective implantation [310]. Among different organs, the heart provides an extremely stable beat rate, and it varies according to the activity intensity; thus, sensors are implanted at this organ for cardiac observation. Wang et al. presented a tissue‐adhesive piezoelectric soft sensor (TPSS) for in vivo analysis, wherein a piezoelectric sensor transformed biomechanical movements into electric pulses, and the adhesive hydrogel (AH) enhanced this output by firmly adhering the sensor to the moist and curved surfaces of biological tissues. The soft sensor is constructed from β‐phase PVDF film, which exhibited stable sensitivity to continuous dynamic bending. Meanwhile, the AH was synthesized from organically sourced mussel adhesive proteins (MAPs) and gelatin. As a pressure sensor, TPSS could be attached to the artery of a grown Yorkshire pig (∼40 kg) to monitor intraoperative vital signs, including blood pressure, heart rate, and respiration rate. In detail, the implantable sensor was mounted to the right internal carotid artery, and the electrodes were connected to an electrometer. This TPSS pressure sensor recorded the biomechanical movements of tissue and converted them into discernible piezoelectric impulses. Simultaneously, a commercial sensor with a probe inserted into the femoral artery was the reference. The data acquisition (DAQ) system of this commercial device was utilized to show the arterial blood pressure (ABP) and electrocardiogram (ECG). The digital images captured the location of the TPSS sensor on the right‐side internal carotid artery (Figure 14a). Under the blood pressure in the internal carotid artery, the TPSS distorted and generated voltage. Following dopamine injection, arterial blood pressure (ABP) gradually ascended from 55 mmHg (low region) to 110 mmHg (high region), corresponding to the TPSS's *V_OC_* increment, and it also repsonded well to the respiration pattern (Figure 14b,c). Interestingly, after operation, the TPSS was simply removed without harming adjacent tissues. The amalgamation of adhesive hydrogel and self‐powered pressure sensors facilitated biocompatible, seamless, and enhanced interactions between biological systems and implantable bioelectronics [311].

Aside from cardiac, implantable sensors for ligament strain are improving swiftly to provide real‐time, accurate monitoring of tissue mechanics for injury diagnosis, surgical repair, and rehabilitation. Yang et al. recently developed an implantable wireless suture sensor for in situ tendon and ligament strain monitoring. The suture‐based strain sensor functionalized with the biocompatible, conductive PEDOT:PSS and encapsulated in medical‐grade silicone. The sensor's substrate is made of medical stitches, which make tissue suturing easier and more biocompatible. The functionalized sutures are woven into a double‐helix structure, resulting in a variable capacitance that responds to mechanical stretching. An inductance coil is integrated to allow wireless and real‐time readout using an inductor and capacitor (LC) circuit (Figure 14d). The sensor's working principle involves producing a change in capacitance based on the magnitude of strain. It interacted wirelessly via a custom‐designed radiofrequency circuit. A passive LCR resonant circuit was formed by incorporating a capacitive sensor (C)s with an inductive coil (L). When the suture sensor is attached to the target tissue, changes in resonant frequency indicate the tendons or ligaments stretching. The device was implanted into the anterior cruciate ligament reconstruction (ACLR) model. As shown in Figure 14e, the autograft containing the sensor was attached to the cortex of the femur and tibia by suture buttons. Once the implantation was complete, the suture‐based sensor's efficiency was investigated by the Lachman test. Conversely, the significant fluctuations in frequency on the loose knee joint revealed compromized and disordered strain shifts (Figure 14f). With in vivo implantation, the author stitched the sensor into 2 rabbit's Achilles tendons: one intact and one half‐broken (Figure 14g). Once the in vivo implantation was finished, the device's sensing ability was carried out, overall, it was clear that the frequency difference between the two specimens was recognized, confirming the suture‐based sensor's excellent monitoring performance in complex anatomical tissues (Figure 14h). The in vivo strain data offered by this sensor system also help surgeons monitor implant‐related problems, develop innovative, more kinematically correct reconstructive approaches, and design tailored rehabilitation programmes [312].

Recently, ferroelectric polymers have been discovered for implantable sensors, neural modulation, and tissue engineering. Du's research group has been investigating different ferroelectric bioelectronics made from PDA, PVDF‐TrFE polymers with other ferroelectric fillers, including BTO, CNT, etc. These devices exhibited various piezo‐coupling effects (piezoelectric, ferroelectric, photopyroelectric), biostability, and flexibility [313, 314, 315]. For instance, a ferroelectric living interface for fine‐tuned exosome secretion (LIFES) with unique multifunctionality properties was reported. The LIFES's interface could be made via electrospinning to improve the interfacial integration with tissues (Figure 14i). Although the classical PVDF‐TrFE/PDA composite was chosen, the authors elevated its piezo‐coupling effect (topographical, piezoelectric, and photo‐pyroelectric), especially with PDA, it possessed high responsivity to NIR irradiation (Figure 14j,k). Upon combination with mesenchymal stem cells, the devices could durably and effectively facilitate vascular neural networks reconstitution and secrete exosomes [313].

Besides sensitivity, there are other factors that contribute to the clinical translation of implantable piezo‐coupling sensors. First, to integrate into the internal organs, the long‐term biocompatibility of these devices needs to be considered, as common polymers like PVDF use toxic solvents and have poor biodegradability, causing immune responses in the organs. To date, many biodegradable polymers and their composites have been developed to address this problem, such as chitosan, celluloses, PCL, etc.; most of them could sustain for several weeks. Yang et al. reported a piezoelectric bio‐organic thin film made from biodegradable γ‐glycine/PVA composite, once implanted under the dorsal region of SD rats, the film did not cause any inflammation or anemia in the test subject throughout 4 weeks [316]. Li et al. fabricated piezoelectric nanofiber mats that were implanted into vital organs, and after 35 days, there were no signs of deformation and lymphatic cell invasion [173]. However, extending the bio‐compability up to months is still a challenge, as the macrophages keep detecting the implanted sensors, leading to foreign body reaction (FBR) and fibrosis, biofouling, thus reducing the sensors and causing infection [317]. Packaging is a common approach to insulate the sensor from body fluids, and packing stability must be balanced between performance and protection. Core/shell nanofibers, adhesive hydrogels, or hydrophobic coatings are recent tactics to improve the packing layers’ reliability. Nevertheless, the main challenges relate to the adverse relationship between mechanical properties and protecting capability [318]. While rigid encapsulants are durable, they could damage the tissue and have a mismatched device‐tissue interface; in contrast, the soft counterparts can fix these problems but have less protection performance. Additionally, moisture/water ingress is unavoidable; leaks will be created and cause delamination, and the barriers are more prone to extreme pH environments, even with chemically resistant polymers [319]. It is also noteworthy that these protection barriers are still foreign materials and would be recognized by FBR eventually.

Understanding the irresistible FBR, researchers have been developing biosafety self‐degradable sensors. This concept relies on the predictable dissolve of the sensors after their service lifetime without removal surgery. However, anticipating the degraded time within the clinical window is extremely hard, as it demands the regulation of various coupled, unpredictable variables. For instance, although the biodegradable piezoelectric polymers degrade by different processes (hydrolysis, oxidation, etc.), one process usually dominates others under certain conditions. Besides, the polymers’ crystallinity or crosslinking degrees can dramatically alter the degradation kinetics, which could shorten or lengthen the materials’ life expectancy. As mentioned above, actual environmental physiological conditions are different from the lab and vary across subjects, which makes predictable in vivo degradation much more complicated than in vitro counterpart [318, 320]. Generally, long‐term biocompatibility, packing stability, and controlled biodegradability are the main bottlenecks for in vivo, implantable sensing devices.

### 5.3 Drug Delivery/Wound Healing Modules

Owing to their mechanical flexibility and biocompatibility, polymeric materials exhibiting piezo‐coupling effects have garnered increasing attention in biomedical applications [321, 322, 323, 324]. As previously discussed, these devices can be either attached to the skin or implanted within targeted internal regions to enable continuous health monitoring. Recent advancements have further expanded their functionality, allowing these systems to harness biomechanical energy not only for sensing but also for on‐demand therapeutic functions, including targeted drug delivery and treatment [325, 326].

#### 5.3.1 On‐Demand Drug Delivery

On‐demand drug delivery system offers a non‐invasive alternative to conventional oral or injectable routes by enabling the controlled release of therapeutic agents through the skin. This approach facilitates direct absorption into the systemic circulation or localized tissues, thereby minimising patient discomfort and reducing adverse effects associated with traditional drug administration approaches [327, 328, 329, 330].

**FIGURE 15:**
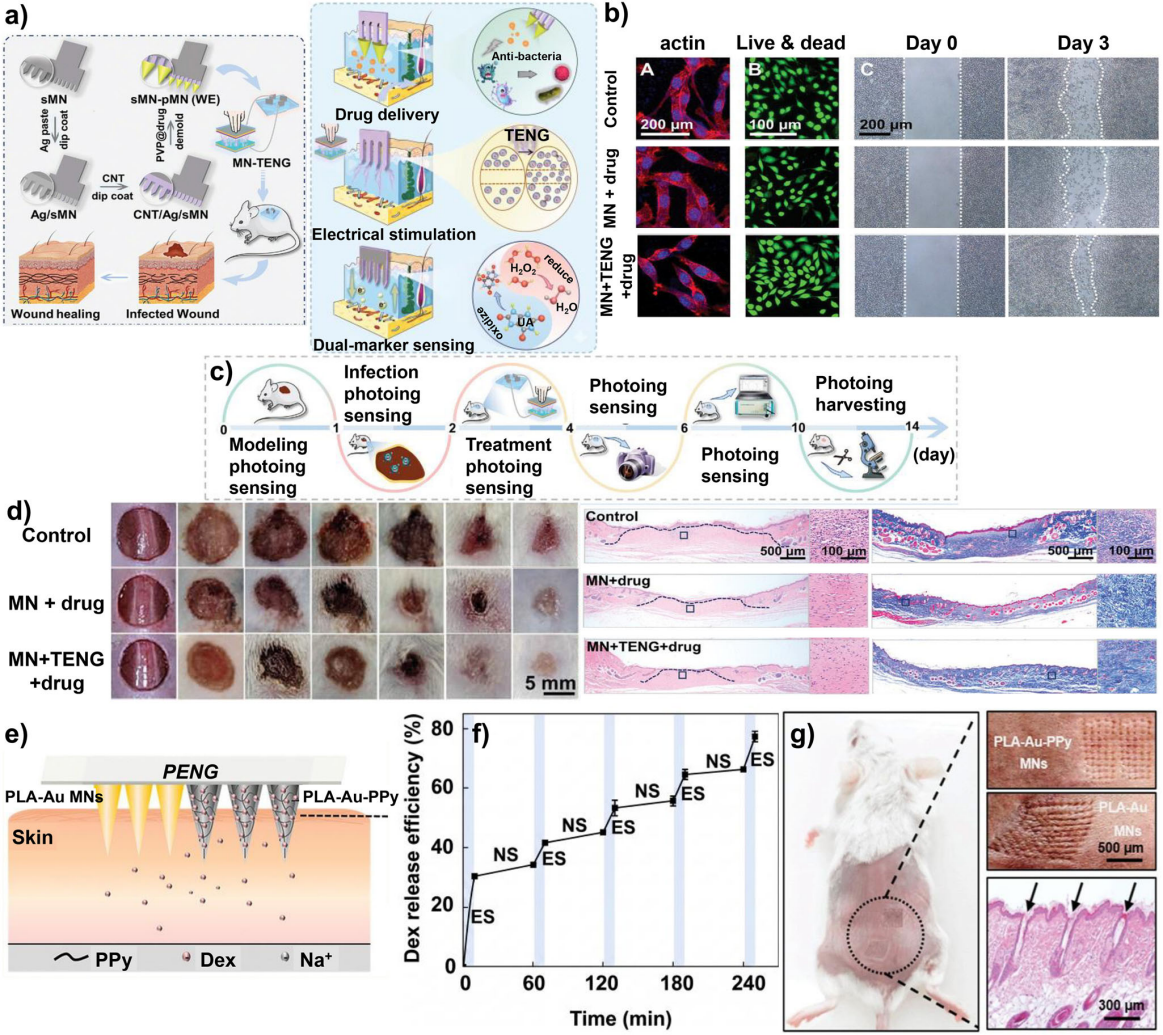
(a) Schematic illustration of the fabrication (left) and operation (right) of the MN‐TENG‐based theranostic platform; (b) Cells proliferation and migration tests: A‐Fluorescence images depicting DAPI and TRITC Phalloidin stained L929 cells; B‐Live/dead photos of L929 cells; C‐Representative images from the scratch tests for the cells migration of different treatments; (c) Schematic diagram of the experimental procedures for establishing and managing *S. aureus* infected wounds utilizing the theranostic platform; (d) Left: representative images and of the wound under three treatment conditions for 14 days; Center: H&E staining images of skin tissue sections obtained from wound areas on day 14 (Black dashed lines denote the boundary between epithelium and dermis), Right: Masson's trichrome staining images of the healed skin wound at day 14. Reproduced with permission [35]. Copyright 2024, John Wiley and Sons; (e) MNP included PLA‐Au MNs and PLA‐Au‐PPy MNs loaded with Dex; (f) Dex release efficiency from the MNP was achieved through intermittent driving of the PENG, utilizing electrical stimulation (ES) for 10 min and no stimulation (NS) for 50 min; (g) Mouse skin post‐treatment with MNP and the associated histological section. Reproduced with permission [333]. Copyright 2021, John Wiley and Sons. The self‐powered transdermal drug delivery system works based on piezo‐coupling effect for drug release and enhanced deep‐tissue penetration, facilitating non‐invasive, highly effective therapeutic.

Piezo‐coupling effects in mechanical self‐powered sensors are widely used in transdermal drug delivery system (TDDS) because physical forces are always available in terms of source variation and force intensity [331]. With much superior flexibility, higher output, and different operational modes, polymeric TENGs have been utilized extensively in this field, whilst other phenomena could play supportive roles to further boost the efficiency. To delivery drugs deeper, TENGs with microneedles were invented. When the microneedle arrays can penetrate the epidermis painlessly, at the same time, the electrical stimulation produced by TENGs enhances skin permeability and facilitates the penetration of drug‐loaded nanoparticles or compounds into deeper tissue layers, which is advantageous for addressing deep‐seated diseases [35, 332]. For instance, Wang et al. reported a microneedle‐integrated TENG (MN‐TENG) as a self‐powered TDDS. Overall, the device consisted of stainless‐steel microneedles (sMN) coated with PVP@drug to make a soluble pMN (polyvinylpyrrolidone microneedles). Here, the metal MN played the role of penetrating skin while the pMN with antibiotics released the drug continuously upon disintegration in the interstitial fluids. Due to the conductive properties of metal, sMN exhibited good conductivity; therefore, it was used as an electrode in a TENG made from PET‐PTFE, which enhanced cell proliferation and migration, ultimately allowing for deeper drug delivery (Figure 15a). The impact of the TENG on cell proliferation was proved by analysing the live and dead photos of L929 cells, with the PET‐PTFE energy harvester, the cell viability was much higher than that without it (Figure 15b‐B). Regarding cell migration, the L929 cells stimulated by the TENG exhibited a significantly faster migration speed (Figure 15b‐C). An in vivo study was done to further elucidate the wound‐healing ability of the MN‐TENG. Skin defects with a diameter of 8 mm were created on the dorsum of mice. All wounds were inoculated with *S. aureus*, then each dorsum was treated with the different methods. The digital images and healed area percentages of the wounds from day 0 to 14 were captured, it was clear that the MN‐TENG+drug treatment had the highest efficiency, followed by MN+drug (Figure 15c). Additionally, H&E and Masson trichrome staining were employed to further validate the wound healing performance; all indicated MN‐TENG+drug was the most effective treatment (Figure 15d) [35].

Piezo‐based TDDS is promising due to its wearability, compactness, and decent electric yield. Yang et al. presented a self‐powered, controllable transdermal drug delivery system (sc‐TDDS) with a good therapeutic effect that is suitable for TDD. The system is applicable on the skin, with the PENG harvesting and converting biomechanical energy into electricity through various motions, while the MN patch released the drug into skin tissue in response to the electrical signal. The patch included two MN arrays: a polylactic acid‐gold‐polypyrrole (PLA‐Au‐PPy) MN array serving as the working electrode (WE) and the PLA‐Au array functioning as the counter electrode (CE). Through the reversible redox properties of the PPy film, the MN patch facilitated drug loading and release. During the oxidation and deposition of the PPy film, the anionic drug Dex was directly incorporated into the PPy backbone to facilitate drug loading (Figure 15e). During the reduction of PPy film at a specific voltage, Dex was de‐doped from PPy to facilitate drug release. Under the piezoelectric stimulation, the Dex‐releasing efficiency was improved over time, and it could be controlled by activating/deactivating the PENG (Figure 15f). To practically validate the performance of the sc‐TDDS, sodium fluorescein (Flu) was loaded in the PPy film and then released by electrical stimulation from PENG (1.5 V and 2 µA). As shown in Figure 15g, MNP established permanent puncturing sites on the skin surface and created penetration cavities (approximately 500 µm) in the epidermis layer of mouse skin, demonstrating that the MNs effectively penetrate and fully release Dex into skin tissue. This device was lightweight, flexible, and had on‐demand drug delivery, which paved the way for self‐powered treatment [333].

Besides microneedles, iontophoresis is also a potential TDDS, it uses electric current for drug delivery by guiding charges onto the stratum corneum [334]. Recently, a self‐powered, wearable iontophoresis system (TENG‐RIS) was developed for interstitial fluid lactate extraction and detection by Zhang et al. The device could work under in vitro and in vivo conditions. For the in vitro test, the TENG‐RIS patch was attached to the lactate gel surface. With reverse iontophoresis (RI), the green value decreased significantly as the lactate concentration rose, pointing out that the lactate moved to the acceptor chamber by the TENG‐RIS. For the in vivo experiment, the TENG‐RIS patch was applied to the mouse skin around its groin, and the device was used to extract lactate from the mice after 15 min of swimming. The extracted lactate was analyzed using a commercial assay kit, and it was clear that, after exercise, the lactate concentration extracted from the TENG‐RIS patch was much higher. Overall, the TENG‐RIS system with scalable materials demonstrated stable output and guaranteed skin safety in many conditions, thereby supporting its potential for future clinical applications and healthcare technologies [335].

#### 5.3.2 Active Wound Healing

**FIGURE 16:**
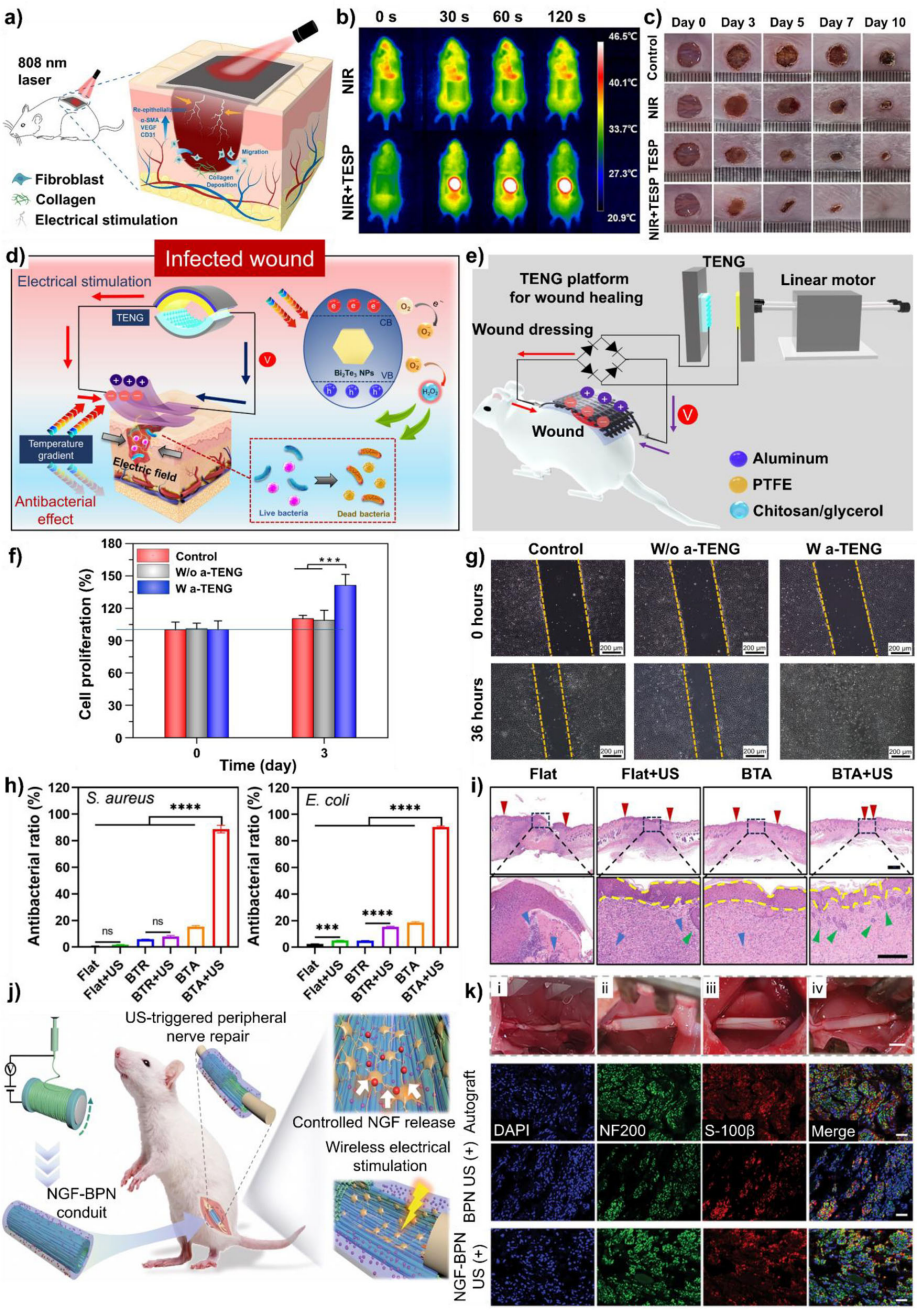
(a) Schematic illustration of how the Mxene TESP heals the wound under NIR; (b) Thermal images depicting temperature variations in mice subjected to treatment with or without TESP and then by 808 nm NIR laser at 0.7 W cm^−2^ for 120 s; (c) Photos of the wounded areas gradually healed after 10 days by different techniques. Reproduced with permission [36]. Copyright 2024, Elsevier; (d) Infected wound dressing mechanism of the self‐powered wound dressing; (e) Working mechanism of how the a‐TENG could stimulate the wound dressing on the mouse; (f) Cell proliferation (%) of fibroblasts in day 0 and day 3 of the Control, W/o a‐TENG, and W a‐TENG samples; (g) Cell migration images of fibroblasts of the Control, W/o a‐TENG and W a‐TENG samples after 36 h. Reproduced with Creative Commons CC BY license [337]. Copyright 2023, American Association for the Advancement of Science; (h) Anti‐bacterial efficiency against *S. aureus* and *E. coli* and via live/dead staining; (i) H&E staining images of skin infected by *S. aureus* during treatment by BaTiO_3_/PLLA electrospun fibrous dressings (red arrows: granulation tissue width; blue arrows: inflammatory cells; dashed yellow lines: epithelium and dermis borders; green arrows: hair follicles) [180]. Reproduced with permission. Copyright 2024, American Chemical Society; (j) Schematic illustration of the ultrasound‐responsive BPN for nerve healing. Reproduced with permission [342]. Copyright 2024, John Wiley and Sons; (k) Top row: Digital photos of different implanted models, from left to right: (i) autograft, (ii) BPN US(−), (iii) BPN US(+), and (iv) NGF‐BPN US(+), remaining rows: Immunofluorescent staining of DAPI, NF200, and S‐100β of, three treatment groups after 8 weeks. Reproduced with permission [342]. Copyright 2024, John Wiley and Sons. These wearable/implantable piezo‐coupling polymeric devices could convert mechanical resonance to electrical signals for the stimulation of tissue regeneration, antibacterial activation, and nerve recovery acceleration without external power supplies.

Wearable and implantable piezo‐coupling polymers can generate electrical power or therapeutic effects without reliance on external batteries. Designed as on‐skin patches or miniaturized modules for internal implantation, these devices can accelerate tissue regeneration and alleviate both physical and psychological discomfort [336]. Mao et al. reported a photothermal single‐electrode TENG for accelerating wound healing. The device (TESP) comprised a MXene/gelatin composite with conductive and photothermal properties surrounded by silicone rubber. The TESP could improve the wound healing under near‐infrared radiation (NIR), as illustrated in Figure 16a. For wound healing application, initially, the photothermal effect of Mxene was proved by thermal pictures, with the conductive agent, the temperature rose rapidly after 120 s (Figure 16b,c). When combined with NIR, TESP displayed a huge wound recovery ability in comparison to other methods while still maintaining the same body weight during the treatment period [36]. Barhman et al. also presented a flexible wound dressing for treating infected wounds. On the one hand, this dressing contained thermocatalytic bismuth telluride nanoplates (Bi_2_Te_3_ NPs), which reacted under temperature change to release hydroperoxide (H_2_O_2_) for antibacterial activity. On the other hand, the reported wound dressing was connected to a wearable arc‐shaped TENG (a‐TENG), as the nanogenerator could create electrical stimulation, thus accelerating the wound recovery speed by improving cell proliferation and migration (Figure 16d,e). Under ideal conditions and after 3 days, the a‐TENG created electric stimulation to promote the cell proliferation, the increase is much higher than the Control and W/o a‐TENG counterparts, as taken in Figure 16f. Moreover, under the same period, this a‐TENG also enhanced the fibroblast cells migration, as the TENG could apply an electric field onto these cells. Meanwhile, the cells in W/o a‐TENG and Control samples migrated in arbitary orientations (Figure 16g), [337]. Besides the film‐based polymeric wound healing system, fiber‐based devices were employed to enhance the surface‐to‐area ratio and crystallization. The BaTiO_3_‐doped PLLA aligned fibers, as mentioned in the previous chapter, could eliminate 88.72% and 90.43% of *Staphylococcus aureus* and *Escherichia coli*, respectively, using only ultrasound vibration without any exogenous drugs (Figure 16h). Additionally, a wound closure rate of 95.5% was observed within a 10‐day period. The in vivo results demonstrated that this dressing significantly reduced wound closure time by approximately 2 days, this was associated with decreased inflammation and enhanced re‐epithelialization and angiogenesis (Figure 16i) [180].

Furthermore, the piezo‐coupling sensors are being studied for nerve healing and generation. These sensors use the piezoelectric effect to transport localized electrical pulses that improve nerve cell proliferation, guide axonal growth, and speed up recovery. By using flexible and biodegradable polymers, the devices have the potential to bring better nerve recovery efficiency. Through body motion or ultrasound, the piezo‐coupling sensors generate localized electrical signals that emulate intrinsic bioelectric signals for nerve regeneration. In other words, the piezoelectric excitation supports Schwann cell proliferation, axonal growth, and myelination [338, 339, 340, 341]. Xu et al. fabricated aligned BaTiO_3_/PVDF‐TrFE nanofibers coated with thermoresponsive poly(N‐isopropylacrylamide) hydrogel conduits for peripheral nerve regeneration via nerve guidance conduits (NGC). Due to the alignment and outstanding piezoelectricity, the aligned fibrous composite could facilitate directional neuronal extension and yield piezoelectricity to enhance neurite growth and cellular differentiation when subjected to ultrasound. Meanwhile, the hydrogel was shrunk because of ultrasound, releasing the nerve growth factor (NGF). Altogether, a hybrid fibrous‐hydrogel composite was synthesized and called BPN (Figure 16j). The nerve healing performance of the hydrogel was evaluated, and the hydrogel samples were transplanted into the sciatic nerve defects in rats. The rat sciatic nerve defect models were categorized into different groups: autografts, BPN NGCs with ultrasound (BPN US(+)), and NGF‐loaded BPN NGCs with ultrasound (NGF‐BPN US(+)). After 8 weeks, the immunofluorescent stainings of DAPI, NF200, and S‐100β were captured; it was clear that the NGF‐BPN US(+) model had a much higher marker density than other two treatments and was comparable to the autograft (Figure 16k). The results illustrated the enhancing effects of US‐induced wireless electrical stimulation and regulated NGF release on nerve regeneration [342].

### 5.4 Human–Machine Interaction and Life Assistance Devices

**FIGURE 17:**
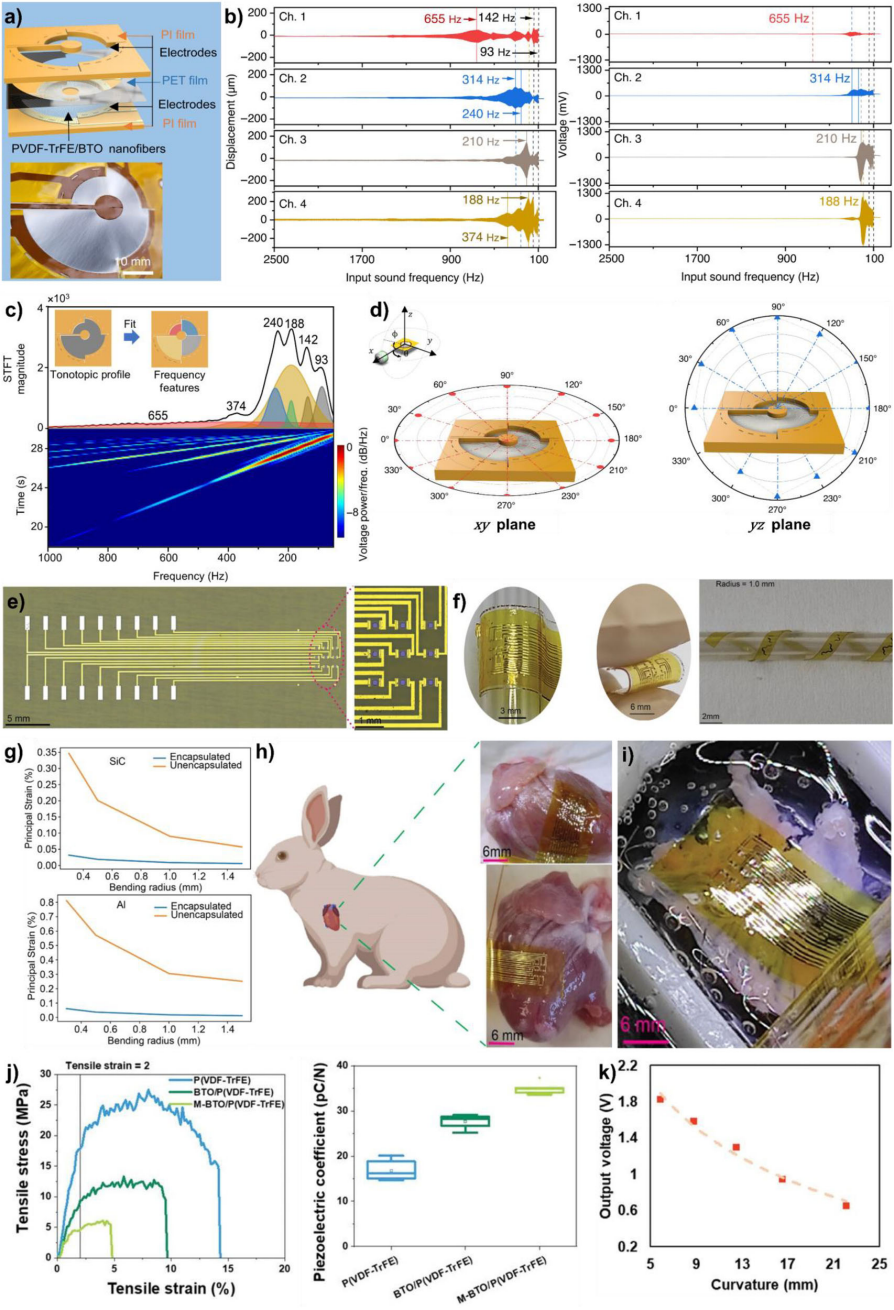
(a) The layer‐by‐layer schematic design of the ST‐PiezoAD; (b) The displacement and voltage of 4 different piezoacoustic channels at the frequency range from 2500 to 100 Hz; (c) Tonotopic profile from the voltage output of the ST‐PiezoAD with four channels connected in series for a cumulative output; (d) Sound recognition precision on the *xy* and *yz* planes. Reproduced with Creative Commons CC BY license [37]. Copyright 2025, American Association for the Advancement of Science; (e) PI‐encapsulated 3 × 3 MEA for sensing and stimulating; (f) The flexible MEA bent around a 3‐mm radius tube, and wrapping around a tube with 1 mm radius; (g) The relationship between principal strain and bending radius of SiC (top) and Al (bottom) with and without the flexible PI; Illustration and real images of the MEA being attached to (h) the rabbit heart and (i) rabbit heart tissue. Reproduced with permission [38]. Copyright 2025, American Chemical Society; (j) The tensile stress–strain test and piezoelectric coefficient comparison tests of different films; (k) The relationship between output voltage and curvature of the BTO/P(VDF‐TrFE) composite. Reproduced with Creative Commons CC BY‐NC license [343]. Copyright 2023, John Wiley and Sons. The piezo‐coupling polymeric materials, along with their flexibility, could extend their application to prosthetic devices and implantable electrodes for internal organs treatment.

The potential applications of piezo‐coupling effects in polymeric materials extend beyond those previously discussed. For instance, researchers have explored this multifunctional capability in the development of advanced prosthetic devices. Chang et al. reported an artificial hearing device from PVDF‐TrFE/BTO fibers. The spiral trampoline‐like piezoelectric acoustic device (ST‐PiezoAD) was designed based on the human cochlea. The outer electrodes are divided into four quarters with different radii, while the inner electrode is in the center and connected to the contour by a cantilever. The PVDF‐TrFE/BTO fibers were electrospun radially between the inner electrode and four outer electrodes, which was similar to the natural basilar membrane of the cochlea. An upper electrode and a polyethylene terephthalate (PET) film were combined with the PVDF‐TrFE/BTO nanofiber‐coated bottom electrode to create the ST‐PiezoAD device (Figure 17a). The vibration displacements and voltage of four channels (corresponding to for quarters with different radii) were shown in Figure 17b. Channel 1,2, 3, and 4 resonated at frequencies of 655, 310, 210, and 190 Hz, respectively, and the voltage was proportional to the displacement. The frequency characteristics of the ST‐PiezoAD were further examined utilising Short‐Time Fourier Transform (STFT) and peak analysis (Figure 17c). Voltage peaks were detected at frequencies of 655, 374, 240, 188, 142, and 93 Hz. The peak frequencies underwent additional analysis using Gaussian fitting algorithms. The piezoacoustic peak of the single‐channel output of the ST‐PiezoAD coincides with the resonant peaks detected in each individual channel. This experiment proved that the ST‐PiezoAD can transduce and summarize intricate sound information by recording features across an extensive frequency range through all four channels. This exceptional ability was due to the use of very sensitive aligned piezoelectric nanofibers and the creation of multiresonant coupling circumstances. Moreover, the ST‐PiezoAD module demonstrated remarkable precision in directional recognition along the polar axis, including a solid angle of 4π. Figure 17d illustrated the classification‐based recognition accuracy on the *xy* and *yz* planes, respectively, utilizing an azimuthal step of 30°. The impact of channel number on recognition accuracy was examined by collecting training data from the ST‐PiezoAD with one, two, and four channels. The ST‐PiezoAD provides several auditory functions, enhancing possibilities to emulate the human auditory system's capabilities [37].

Apart from playing the main functioning roles, the polymer could simply be the flexible, biocompatible substrate for implantable systems. Truong et al. selected polyimide (PI) as the flexible matrix surrounding the multielectrode arrays (MEA); it significantly contributed to the mechanical flexibility of the 3D‐structured (Figure 17e,f). The PI encapsulation layer prevented the SiC and Al from fracture strain (Figure 17g). With this advantage, the encapsulated MEA was attached to the rabbit heart or even the heart tissue for cardiac pacing (Figure 17h,i). The flexible device was able to be attached to the organs and then used for in/ex vivo medical applications [38].

Polymers could act as the piezoelectric‐sensitive polymer matrix in a composite. When combined with additives, the composites have sensitivity whilst maintaining the flexibility for wearable/implantable applications. For instance, Zhou et al. employed the synclastic effect from an auxetic structure to create a stretchable composite (BTO/P(VDF‐TrFE)). P(VDF‐TrFE) significantly improved the tensile strain of the device yet had a decent piezoelectric coefficient (Figure 17j). When bent with different curvatures, the composite provided good sensitivity (Figure 17k) [343].

## 6 Outlooks

### 6.1 Conclusion

This article outlines key stimuli‐responsive sensing mechanisms. Piezoelectric and pyroelectric effects, though triggered by mechanical and thermal stimuli respectively, both arise from spontaneous polarization in non‐centrosymmetric materials. The triboelectric effect, also mechanically driven, results from electron transfer between materials with differing affinities. The photovoltaic effect involves photon‐induced excitation of electrons across the bandgap. Advanced piezo‐coupling systems, such as piezo‐triboelectric, piezo‐pyroelectric, and piezo‐pyro‐phototronic, combine multiple effects to enhance sensitivity and energy harvesting capability. These effects are ubiquitous, which is favorable for ultrasensitive sensing and self‐powered wearable electronic applications.

Polymers are among the most promising materials for wearable and implantable bioelectronics due to their superior flexibility, durability, and biocompatibility. Each physical sensing effect—piezoelectric, triboelectric, pyroelectric, and photovoltaic—requires specific polymeric materials. PVDF and PLLA are effective for piezoelectric sensing, while PTFE and nylon are widely used in triboelectric systems due to their strong electron affinity characteristics. Natural polymers like cellulose offer biodegradability and functional efficiency. In photovoltaics, non‐fullerene acceptors outperform fullerene‐based materials, with PEDOT:PSS serving as a flexible and efficient hole transport layer. PVDF also exhibits strong pyroelectric properties and is commonly used in hybrid systems such as piezo‐phototronic, pyro‐phototronic, and piezo‐triboelectric. Smart polymer composites further enable piezo coupling of multiple sensing effects. Beyond active sensing roles, polymers also serve as flexible substrates to improve device conformability. These smart polymeric materials support applications ranging from motion tracking to respiratory monitoring and implantable biomedical sensors, offering high sensitivity, minimal discomfort, and precise diagnostics. Additionally, stimuli‐responsive piezo‐coupling systems have demonstrated potential in controlled drug delivery, enhancing therapeutic outcomes through uniform release and improved wound healing efficiency.

The article emphasizes the valuable advantages of polymers with piezo‐coupling effects, which are ubiquitous energy sources (mechanical, thermal, light); multimodal principles based on bi‐ or tri‐coupling effects; facile solution‐processing techniques (coatings, castings, electrohydrodynamics, printings); a plethora of polymers and their composites/ceramics for different applications; flexibility/conformality and biocompatibility for wearable/implantable applications. Despite these benefits, there are several bottlenecks for piezo‐coupling polymers that need to be dealt with. In performance terms, they have lower sensing coefficients compared to the rigid inorganics/semiconductors, limiting them from becoming fully closed‐loop sensing platforms. Signal crosstalk and noise are typical problems of piezo‐coupling devices. The output stability is also easily affected by various environmental conditions. Moreover, the performance could be further reduced due to charge dissipation and poor interaction with the electrode layers. About the materials, the synthetic polymers have higher efficiency but lack biocompatibility, as they are often dissolved by toxic solvents. In contrast, the natural counterparts are compatible with organs and could work inside the body for longer periods of time, but the trade‐offs are lower sensitivity and more susceptibility to the environment. Nevertheless, for biomedical applications, both polymers’ subcategories need to face long‐term biocompatibility, packing stability, and in vivo degradation. More detailed challenges and the possible solutions in the future are discussed in the next section.

**FIGURE 18:**
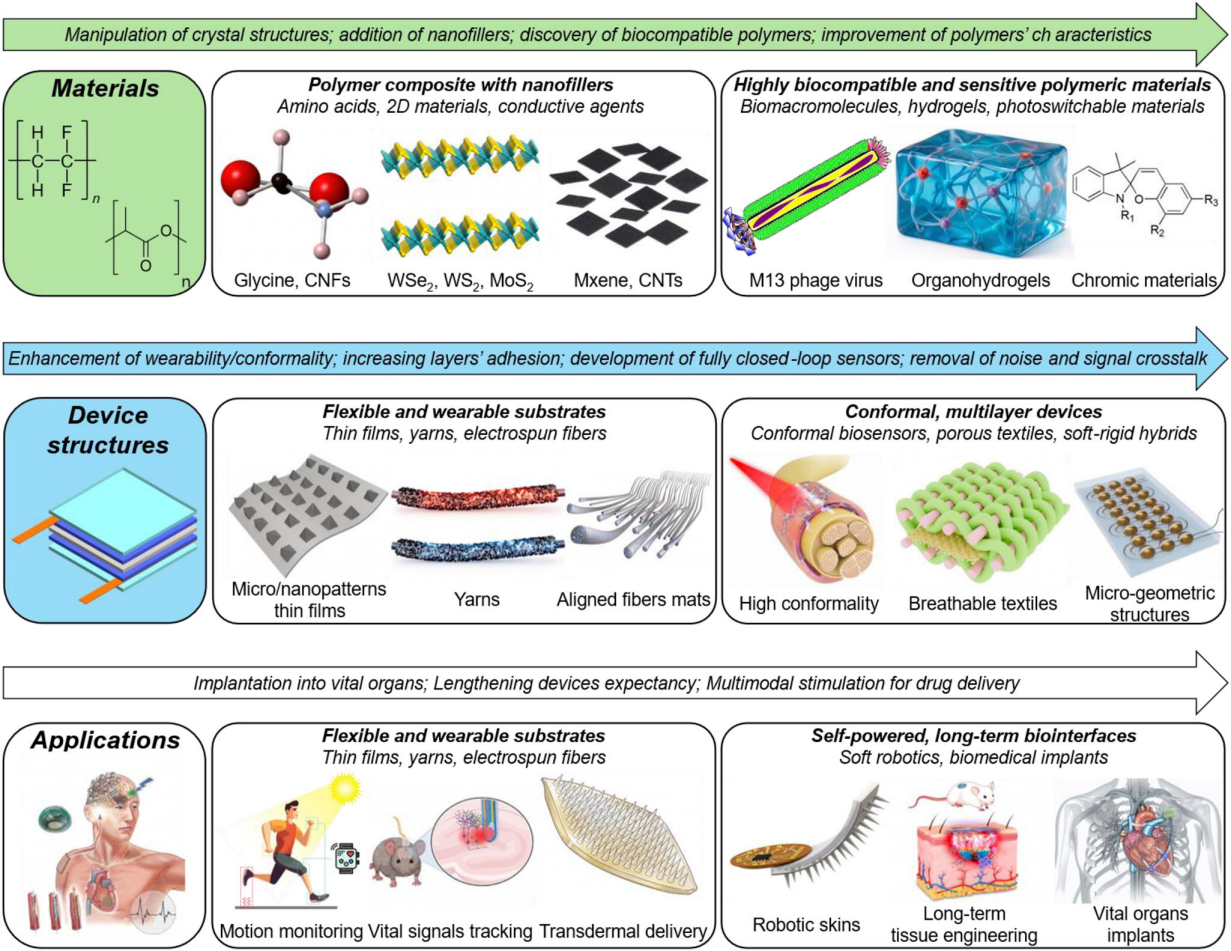
Research trend of piezo‐coupling polymeric devices in three perspectives: materials, structures, and applications. All factors witnessed significant achievements and are still attracting research in the following fields. Reproduced with permission [344]. Copyright 2019, Springer Nature. Reproduced with Creative Commons CC BY‐NC‐ND license [345]. Copyright 2015, Elsevier. Reproduced with Creative Commons CC BY‐NC‐ND license [346]. Copyright 2022, American Chemical Society. Reproduced with permission [314]. Copyright 2025, John Wiley and Sons. Reproduced with Creative Commons CC BY license [347]. Copyright 2026, John Wiley and Sons. Reproduced with Creative Commons CC BY‐NC license [348]. Copyright 2023, American Association for the Advancement of Science. Reproduced with permission [315]. Copyright 2026, John Wiley and Sons. Reproduced with permission [349]. Copyright 2025, John Wiley and Sons. Reproduced with Creative Commons CC BY license [290]. Copyright 2024, John Wiley and Sons. Reproduced with permission [350]. Copyright 2023, Springer Nature. Reproduced with Creative Commons CC BY license [351]. Copyright 2024, Springer Nature. Reproduced with permission [352]. Copyright 2026, John Wiley and Sons. Reproduced with Creative Commons CC BY license [353]. Copyright 2020, John Wiley and Sons. Reproduced with permission [354]. Copyright 2022, American Chemical Society. Reproduced with Creative Commons CC BY‐NC‐ND license [355]. Copyright 2025, Springer Nature.

The development of piezo‐coupling polymeric materials has three main focuses, which are materials, devices' structures, and applications. Pristine polymers with inherent piezoelectricity were fabricated into thin films with decent flexibility and piezoelectric output. Thereafter, other sensing mechanisms were integrated with piezoelectricity, and various composites were synthesized to enhance the electrical conversion efficiency. To improve the performance, various fillers were added into the polymers’ matrices, such as amino acids, 2D materials, conductive agents, etc. The materials' properties were also investigated for better resistance, biocompatibility, and biodegradability. In the future, materials with higher biocompatibility will be examined, and photoswitchable materials would also be a potential direction [344, 345, 346]. Regarding devices' structures, the facile thin films were equipped with micro/nanopatterning for enhanced signal output. Fibers and yarns were fabricated to maximize dipole orientation, polarization, and conformality. Increasing conformality, breathability, and minimizing piezo‐coupling signal crosstalk and noise will be the research direction [290, 314, 315, 347, 348, 349]. For applications, polymeric sensors working under piezo‐coupling effects have achieved many milestones, from physiological signal tracking to wound‐healing patches. So far, soft robotics and non‐infectious biointerfaces continue to attract research efforts (Figure 18) [350, 351, 352, 353, 354, 355].

### 6.2 Challenges and Future Perspectives

Despite the immense potential of piezo‐coupling effects in smart polymeric materials, numerous challenges need to be tackled. In the theoretical aspect, first, the crystallinity mechanisms and amorphous regions of the semicrystalline polymers remain ambiguous [39, 41]. Most research are heavily concerned with the crystalline phase of the polymers but neglect the oriented amorphous fraction. Therefore, future research should deal with a deep explanation of piezoelectric mechanisms, polymer structures, phases, or polymorphs, etc.

Second, for piezo‐coupling effect, due to the complicated interaction between the effects, there is still no combined equation to precisely calculate the combined output. These signals are difficult to capture and besides comparison, there are no reliable ways to realize the impact of each effect in a multi‐physics coupling system. Thus, developing solid models for piezo‐coupling effects would be a potential research field [356, 357]. Another challenge is signal crosstalk, which arises when a multimodal sensor receives simultaneous input sources (e.g., strain deformation under light irradiation of piezo‐phototronic), causing inaccurate measurements. To address cross‐sensitivity, several decoupling strategies have been reported, such as employing polymer composites with multi‐sensing mechanisms (PEDOT:PSS‐PU) or with different response/recovery times (PVDF and its variants). Moreover, unwanted signal crosstalk could be further diminished when integrating suitable materials with proper device structures. The piezo‐coupling wearable sensors also suffered from noise, including environments (motion, temperature, humidity) and materials (viscoelastic damping, dielectric loss). Various approaches are being developed to mitigate the interference, which involves fabricating devices with EMI shields, micropatterns, or choosing conformal polymers (PDMS, Ecoflex) to minimize Young's modulus mismatch against human skin.

From materials perspective, much effort is put into exploring more biocompatible piezo‐coupling polymers. Organic biological piezoelectric polymers, such as amino acids, proteins, and polysaccharides, are fully biodegradable materials, whereas glycine, an amino acid, is reported to have piezo‐ and ferroelectricity, which facilitates the piezo‐pyroelectric effect [358]. Another innovative natural piezoelectric material class is biomacromolecules, particularly M13 phage virus. Due to their filamentous structure, which is clad with helical proteins, M13 phage viruses resemble human collagen in terms of piezoelectric properties. These viruses could create highly oriented crystal structures. However, most of these natural polymers only own piezoelectric properties, and their performance is inferior compared to the synthetic ones. Thus, improving the piezo output and stability should be a potential research direction to achieve highly biodegradable, implantable sensors. Concurrently, for the well‐known piezo‐coupling materials like PVDF, PLLA, and their composites, the future targets should be increasing biocompatibility with vital organs. The incorporated nanofillers inside these polymers should also be considered, as there are specific dopants for specific piezo‐coupling effects, such as graphene for piezo‐triboelectricity, trimethylamine borane for piezo‐pyroelectricity, or narrow‐bandgap BaTiO_3_ for piezo‐phototronic. Their crystallinity, mixing ratios, and nanostructures might play significant roles in the overall performance and flexibility of the fabricated sensing layer; therefore, optimizing these conditions might bring more capable sensing platforms. Furthermore, because of the high mechanical matching with tissues, scalable manufacturability, etc., different hydrogels are emerging candidates for implantable piezo‐coupling sensors. Notably, the conductive or semiconductive hydrogels are being used as the main sensing layers since they could dramatically improve the devices’ overall biocompatibility, stretching ability, and curability. For wearable/implantable electronics, these materials could be further improved by enhancing the sensitivity, adhesiveness or self‐healing ability.

Optimizing the fabrication process is critical for enhancing the efficiency of piezo‐coupling ultrasensitive sensors. Regarding fabrication techniques, although many methods (aligned electrospinning, annealing, poling) successfully produced polymers with high piezoelectric coefficients, the fabrication conditions are inconsistence among research, and there is yet any optimal fabrication standards for these polymers. This challenge becomes even more obvious when combining the polymers with other components. Furthermore, to reach industrial scale, other struggles still exist. For instance, electrospinning could create highly aligned fibers, but it lacks alignment consistency when making a large, thick fibrous mat. Reproducibility is another concern when slight changes in the environment can cause huge differences [359, 360]. Moreover, these fabrication challenges become bigger with the composites or polymeric‐based ceramics. The polymer matrices have different dielectric coefficients and Young modulus values from the additives, causing a mismatch between the component thus lowering the output and wearability. Accordingly, the optimization of polarization and stress transfer between the polymers and additives should be solved. And due to the limited selection of materials, fabricating biocompatible piezo‐coupling devices is difficult. Strategic material arrangement is essential for multifunctional sensor performance. Furthermore, the synthesis of OPV components in hybrid optoelectronic systems remains complex and resource‐intensive, involving multiple steps and costly materials. Streamlining these fabrication processes is vital to facilitate broader adoption and further research in this field.

About the performance, the biggest problem of piezo‐coupling polymers is inferior performance compared to semiconductors and single‐crystal counterparts. Doping nanofillers will enhance the output but sacrifice flexibility and elasticity. Moreover, output stability should also be addressed since there are many factors that could affect the devices’ protracted performance, such as mechanical fatigue, interfacial charge trapping, humidity, and temperature. Another emerging problem is the trade‐offs between sensing performance and mechanical robustness. As mentioned above, one of the conventional ways to improve sensitivity is the addition of nanofillers, which reduces the elasticity and flexibility of the sensors.

High‐impedance output is another challenge, where most piezo‐coupling devices could produce high voltage but low current, preventing them from being fully closed‐loop gadgets. This problem is being addressed by connecting the device to a power‐management circuit for piezo‐coupling electricity storage; however, these circuits are often bulky and complicated. Consequently, size reduction and simplifying every component of the fully autonomous sensing systems are interesting research directions.

For application, there is a lot of potential for wearable devices based on piezo‐coupling effects. As mentioned above, achieving stable performance for these systems needs to be dealt with before considering the wearability factor. On the one hand, wearable device packaging needs to take into consideration to preserve the performance and user's comfort. On the other hand, implantable gadgets require more attention to biocompatibility, interactions with organs, and suitable stimulation thresholds.

In the current era, the role of artificial intelligence (AI) is becoming increasingly significant. It can contribute in every part to the fabrication of polymeric piezo‐coupling sensors. Developing innovative polymers or composites requires thorough chemical and materials knowledge, and AI can help predict the electrical output, mechanical, and biocompatible properties by summarizing previous research and fundamental concepts. Next, the polarization, charge generation, and migration across the sensors can be simulated faster with the help of AI, which is extremely useful for researchers who are not specialized in chemical computing. However, at these initial development stages, AI suggestions should be considered carefully, as its knowledge in heavily scientific content is still limited, both in quantity and quality. Regarding data processing, AI can be integrated with machine learning (ML) to classify signal groups. Understanding the rapid learning speed from extensive data collections, many researchers built and integrated ML+AI algorithms to learn and then instantaneously detect different motions with very high accuracy. For piezo‐coupling wearable/implantable sensors, this combined technology could deal with filtering signal noise and signal crosstalking. The most common functions include detecting baseline drift caused by environmental factors and recognizing signal patterns. Nevertheless, the detailed coding methods should be shared widely so that researchers can find out the best algorithms for their devices or improve the ML+AI efficiency. In summary, AI could assist hugely in brainstorming for innovative devices, structure optimization, and data interpretation, but it should be used with extra care, as its knowledge in highly scientific content is still modest.

## Author Contributions

**Tuan Sang Tran**: conceptualization, writing – review and editing, funding acquisition. **Duc Khanh Tran**: conceptualization, writing – original draft, writing – review and editing, methodology. **Van Thanh Dau**: conceptualization, funding acquisition, writing – review and editing. **Dzung Viet Dao**: writing – review and editing, funding acquisition. **Thanh‐An Truong**: writing – review and editing. **Trung‐Hieu Vu**: writing – review and editing. **That Buu Ton**: writing – review and editing.

## Conflicts of Interest

The authors declare no conflicts of interest.

## Data Availability

The data that support the findings of this study are available from the corresponding author upon reasonable request.
